# Burden and management of venous thromboembolism in children and adolescents (2004–2023): a Swiss nationwide epidemiological study

**DOI:** 10.1007/s00431-025-06598-4

**Published:** 2025-11-17

**Authors:** Simon Wolf, Manuela Albisetti, Alessandra Bosch, Suzanne C. Cannegieter, Frederikus A. Klok, Alice Trinchero, Luca Valerio, Nils Kucher, Stefano Barco

**Affiliations:** 1 Department of Vascular Medicine, University Hospital Zurich, University of Zurich, Zurich, Switzerland; 2 Department of Medicine, Thrombosis and Hemostasis, Leiden University Medical Center, Zuid-Holland, Leiden, the Netherlands; 3 Division of Hematology and Children’s Research, Center, University Children’s Hospital Zurich, Zurich, Switzerland; 4 Department of Medical Oncology and Hematology, University Hospital Zurich, Zurich, Switzerland; 5 Center for Thrombosis and Hemostasis, University Medical Centerof the, Johannes Gutenberg University, Mainz, Germany; 6 Department of Clinical Epidemiology, Leiden University Medical Center, Leiden, Netherlands; 7 Department of Angiology, University Hospital Zurich, Raemistasse 100, Zurich, 8091 Switzerland

**Keywords:** Pediatric patients, Deep vein thrombosis (DVT), Length of stay (LOS)

## Abstract

Epidemiological data on venous thromboembolism (VTE) in children and adolescents is sparse and available primarily for the USA. In Europe, the burden and management of VTE have rarely been subjects of nationwide analyses.

We conducted a nationwide, retrospective, patient-level analysis of the Swiss medical statistics an administrative data collection including 1961 hospitalizations (1653 incident events) with VTE aged 19 or younger between 2004 and 2023. We estimated the disease-specific incidence rate, in-hospital case fatality rates, length of stay (LOS), and related admission to the intensive care unit (ICU). Furthermore, we evaluated therapeutic procedures.

The incidence rate of incident VTE-related hospitalizations was 4.9 (95%CI 4.7; 5.2) per 100,000 children and adolescents per year. The in-hospital case fatality rate was 4.0% (95%CI 3.2; 5.0); the median LOS was 8 (Q1–Q3 4–24) days. Systemic thrombolysis, catheter-directed treatment, and inferior vena cava filters were used in less than 15% of patients. Among patients receiving systemic thrombolysis, the in-hospital case fatality rate was 19.4% (95%CI 12.6; 28.5), whereas patients managed with catheter-directed treatment had a rate of 8.0% (95%CI 3.2; 18.8). 39.3% of patients were admitted to ICU. Age- and sex-specific differences in incidence rate, proportion of hospital admissions, and in-hospital case fatality rate were observed, particularly among infants aged < 1 year and adolescents aged 15–19 years.

*Conclusion*:VTE among children and adolescents is rare with age- and sex-specific differences in incidence. Patients who underwent advanced treatment strategies presented with a substantial risk of in-hospital death despite treatment, especially evident among patients aged 0–9 years.

**Table:** 

**What is Known:** • *Venous thromboembolism and in extension pulmonary embolism and deep vein thrombosis are rare diseases among children and adolescents.*
**What is New:** • *We provided national estimates for measures of occurrence of venous thromboembolism from a European country.* • *We analyzed the management of these patients with a focus on advanced treatment strategies, providing estimates on the in-hospital fatality rate.*

**Supplementary Information:**

The online version contains supplementary material available at 10.1007/s00431-025-06598-4.

## Introduction

Venous thromboembolism (VTE) and its two most severe manifestations—acute pulmonary embolism (PE) and deep vein thrombosis (DVT)—are rare in children and adolescents [1–3]. Recently, a study provided data on the epidemiology of acute PE among children and adolescents in the USA. Overall, the incidence rate was found to be 3.5 PE-related hospital admissions per 100,000 children and adolescents per year, corresponding to a proportion of 13 PE events per 10,000 hospital admissions [1]. Concerning DVT, little contemporary data are available. In the past, an incidence rate of 0.7 to 4.2 DVT diagnoses per 100,000 children and adolescents per year and a proportion of 5 to 50 DVT-related hospital admissions per 10,000 hospitalizations have been estimated [3–6]. Both for VTE overall and for PE specifically, a notable increase in the proportion of hospital admissions has been described [7, 8]. Nevertheless, the epidemiological burden of VTE in pediatric patients remains insufficiently studied on a global scale.

Management algorithms for VTE, PE, and DVT in pediatric patients, particularly concerning the role of reperfusion and interventional (catheter-directed) treatment, are poorly established. The 2019 European guideline on the management of acute PE does not address the treatment of PE in children and adolescents [9]. The 2018 guidelines on the treatment of pediatric venous thromboembolism by the American Society of Hematology recommended against interventional treatment with the lowest level of evidence [10], whereas the guidelines updated in 2024 do not provide recommendations [11]. Nevertheless, catheter-directed treatment in selected pediatric patients with PE has been proposed as a possible alternative to systemic thrombolysis or surgical embolectomy [12]. In light of heterogeneous recommendations and the lack of data on the optimal interventional treatment [13], the most suitable treatment option for pediatric patients with high-risk PE remains uncertain.

The aim of the present study was to provide an overview of measures of occurrence and clinical outcomes including incidence rate, proportion of hospital admissions, in-hospital case fatality rate, and length of hospitalization (length of stay; LOS) of VTE in Switzerland among 0–19 years old infants, children, and adolescents, distinguishing between acute PE and DVT. Furthermore, we aimed to describe the management, particularly regarding reperfusion and interventional treatment on a nationwide level.

## Methods

### Data source

We conducted a patient-level retrospective analysis of Swiss healthcare data collected by the Swiss Federal Statistical Office, including all patients 19 years or younger hospitalized in Switzerland from 2004 to 2023. This age group was chosen to ensure comparability with previous studies [1]. Additionally, the epidemiology of acute PE in Switzerland for patients 15 years or older has already been described [14].

The Swiss Medical statistics is a data collection of all hospital discharge data in Switzerland at the individual patient level [15]. By law, healthcare institutions are required to collect discharge data and to report it annually to the Swiss Federal Statistical Office. Due to the mandatory nature of the collection, data are assumed to be missing for less than 1% of hospitalizations. Data are anonymized to comply with personal data protection regulations. Diagnoses are collected in accordance with the International Classification of Disease, 10th revision with German modifications (ICD-10-GM). Performed procedures are collected using the Swiss classification of surgical interventions (CHOP) codes. Hospitals undertake local measures to verify and amend discharge ICD-10-GM and CHOP codes based on actual diagnoses and procedures performed during each admission. At a national level, code review is performed by independent reviewers and must be completed by May 31 of the year X + 2 [16].

Patients were selected based on the mention of I26 and O88.2 the ICD-10-GM codes for PE, and of I80 (excluding. I80.0 and I80.9) and I82.2 the ICD-10-GM code for DVT of the lower extremity and the vena cava (Table S1). VTE was defined as any mention of the previously stated codes. Other codes were not included in the analysis due to limited sensitivity (e.g., the code for thrombosis of the upper extremity does not discern between the superficial and deep venous system). All these codes capture both patients admitted with or for VTE and patients that developed VTE during the hospitalization without discriminability of these two groups. Patients with PE and concomitant DVT diagnosis were classified as PE patients and excluded from the DVT group. Table S2 presents ICD-10-GM codes for clinically selected comorbidities. ICD-10-GM codes and CHOP codes for the definition of high-risk features (cardiopulmonary resuscitation, use of vasopressor or thrombolytic substances, extracorporeal membrane oxygenation [ECMO], mechanical ventilation, surgical embolectomy, shock, cardiac arrest) are presented in Table S3. Table S4 presents the procedure codes considered for the definition of therapeutic procedures. A unique code for catheter-directed interventional treatment was introduced in 2008. At the start of data analysis, data up to 2023 was available.

In Switzerland, healthcare facilities are categorized into general hospitals and specialized clinics, based on the number of medical specialities, teaching competencies across medical specialities, and the reported inpatient volume. In total, three hospitals are categorized as specialized pediatric hospitals, including two university hospitals [17]. For the purpose of this study, we categorized general hospitals into three groups: (1) university hospitals, (2) major hospitals, and (3) regional hospitals. Specialized clinics were categorized into (4) specialized pediatric hospitals, and (5) other specialized clinics (“Others”).

This study was conducted in accordance with the Reporting of studies Conducted using Observational Routinely collected Data (RECORD) standards [18]. This database is accurately representative of all hospitalizations in Switzerland and has been used in the past for similar purposes [19]. As it provides fully anonymized information, written informed consent by patients and protocol approval by the institutional ethics committee and ethical approval are not necessary.

### Statistical analysis

The primary analysis was performed for all VTE-related, PE-related, and DVT-related cases, defined as the hospitalizations in which VTE, PE, and DVT cases were reported as either primary or concomitant diagnoses. The primary analysis was not restricted to the cases with these conditions stated as primary diagnoses only because prior studies showed that using concomitant diagnoses in the identification of PE increased the sensitivity without severely reducing the positive predictive value [20].

We calculated age- and sex-specific incidence rates for all disease-related hospitalizations and separately for the first disease-related hospitalization as the number of cases per 100,000 children and adolescents per year and the proportion of hospital admissions per 10,000 hospital admissions. We calculated age-specific in-hospital case fatality rates and frequency of in-hospital death stratified by the presence and type of high-risk features as the number of disease-related deaths per 100 disease-related hospital admissions. For the primary analyses, patients were divided into 5-year age groups according to the European standard population with a further subdivision of the age group between 0 and 4 years into patients aged 0 years and 1–4 years. For the calculation of rates, patients were additionally grouped into clinically selected age groups (0 year, 1–5 years, 6–12 years, and 13–17 years). Due to the small number of cases per calendar year, time trend analyses were omitted. Instead, analyses were stratified into the first (2004–2013) and second (2014–2023) half of the study period. As a sensitivity analysis, we calculated the incidence rate and proportion of disease-related hospital admissions for VTE, PE, and DVT as the primary discharge diagnosis. No further sensitivity analyses were performed due to low patient numbers, especially in infants and children aged 0–9 years.

In the overall cohort, we performed univariable and multivariable logistic regression to study the impact of different advanced treatment options and mention of PE (vs. DVT) on death, intracranial hemorrhage, a more general definition of hemorrhage (not including intracranial hemorrhage), and stay in an intensive care unit (ICU). Adjustment was only performed for sex; we abstained from performing further adjustment because of the low numbers of outcome events.

Data is presented either as count and percent or, for continuous variables, as median and interquartile range. Continuous variables were visually inspected for data distribution. All rates are presented as crude nationwide rates. Rates and frequencies are presented with appropriate 95% confidence intervals (CI). The statistical analysis was performed using R version 4.4.1.

### Role of the funding source

The authors are solely responsible for the content of this work. No external funding was obtained for this study. The corresponding author had full access to all the data and the responsibility for submission for publication.

## Results

### Population

During the study period (2004–2023), the mean mid-year population in Switzerland aged 19 or younger was 1,674,266. The annual average number of hospital admissions in the same age group was 192,350. A total of 1961 (52.9% female) VTE-related hospital admissions were recorded, corresponding to 1653 individual patients. Among all hospital admissions, a total of 1146 (47.1% female) were DVT-related and 815 (61.0% female) were PE-related. A concomitant DVT diagnosis was present in 172 (20.5% of patients with PE) hospital admissions with PE (Table 1, Table S5-S7).

**Table 1:** Overview of venous thromboembolism cases, and venous thromboembolism-related deaths per age group, stratified by sex

Characteristic	Venous thromboembolism			Pulmonary embolism (with or without deep vein thrombosis)			Deep vein thrombosis (excluding cases with concomitant pulmonary embolism diagnosis)		
	**Overall,** *N* = 1961	**Female,** *N* = 1037	**Male,** *N* = 924	**Overall,** *N* = 815	**Female,** *N* = 497	**Male,** *N* = 318	**Overall,** *N* = 1146	**Female,** *N* = 540	**Male,** *N* = 606
Hospitalizations for or with the disease, *n* (%)									
0 years old	393 (20.0%)	148 (14.3%)	245 (26.5%)	29 (3.6%)	15 (3.0%)	14 (4.4%)	364 (31.8%)	133 (24.6%)	231 (38.1%)
1 to 4 years old	166 (8.5%)	70 (6.8%)	96 (10.4%)	25 (3.1%)	10 (2.0%)	15 (4.7%)	141 (12.3%)	60 (11.1%)	81 (13.4%)
5 to 9 years old	120 (6.1%)	58 (5.6%)	62 (6.7%)	27 (3.3%)	10 (2.0%)	17 (5.3%)	93 (8.1%)	48 (8.9%)	45 (7.4%)
10 to 14 years old	200 (10.2%)	88 (8.5%)	112 (12.1%)	68 (8.3%)	32 (6.4%)	36 (11.3%)	132 (11.5%)	56 (10.4%)	76 (12.5%)
15 to 19 years old	1082 (55.2%)	673 (64.9%)	409 (44.3%)	666 (81.7%)	430 (86.5%)	236 (74.2%)	416 (36.3%)	243 (45.0%)	173 (28.5%)
Median age of patients at hospital admission, years (Q1–Q3)	15 (2, 18)	16 (8, 18)	13 (0, 17)	17 (16, 18)	17 (16, 18)	17 (14, 18)	8 (0, 16)	13 (1, 17)	4 (0, 15)
First decile (D1), years	0	0	0	10	13	6	0	0	0
Death with any mention of the disease, *n* (%)	79 (4.0%)	41 (4.0%)	38 (4.1%)	35 (4.3%)	21 (4.2%)	14 (4.4%)	44 (3.8%)	20 (3.7%)	24 (4.0%)
0 years old	43 (2.2%)	19 (1.8%)	24 (2.6%)	13 (1.6%)	8 (1.6%)	5 (1.6%)	30 (2.6%)	11 (2.0%)	19 (3.1%)
1 to 4 years old	6 (0.3%)	2 (0.2%)	4 (0.4%)	1 (0.1%)	0	1 (0.3%)	5 (0.4%)	2 (0.4%)	3 (0.5%)
5 to 9 years old	3 (0.2%)	3 (0.3%)	0	1 (0.1%)	1 (0.2%)	0	2 (0.2%)	2 (0.4%)	0
10 to 14 years old	7 (0.4%)	4 (0.4%)	3 (0.3%)	3 (0.4%)	2 (0.4%)	1 (0.3%)	4 (0.3%)	2 (0.4%)	2 (0.3%)
15 to 19 years old	20 (1.0%)	13 (1.3%)	7 (0.8%)	17 (2.1%)	10 (2.0%)	7 (2.2%)	3 (0.3%)	3 (0.6%)	0
Median age at disease related death, years (Q1–Q3)	0 (0, 16)	4 (0, 17)	0 (0, 12)	14 (0, 18)	13 (0, 18)	15 (0, 17)	0 (0, 2.5)	0 (0, 10)	0 (0, 0)

Overall, 79 (51.9% female) in-hospital deaths were related to VTE; of these, 22.8% were patients aged 17 years or older. DVT-related in-hospital death was recorded in 44 (45.5% female) cases, 35 (60% female) were PE-related in-hospital deaths. A total of 1647 (84.0%) hospital admissions were in university hospitals, major hospitals, or specialized pediatric hospitals. This was particularly the case for infants and children aged 0–9 years (*n* = 675; 99.4%). The frequency of VTE-related hospitalization was higher in the second half (vs. first half) of the study period, and for male (vs. female) patients (Table S8-S11).

The median age at VTE-related hospital admission was 15 (Q1–Q3 2–18) years. Patients with PE-related hospital admission were older than those with DVT-related hospital admission. The median age at VTE-related in-hospital death was 0 (Q1–Q3 0–16) years. Patients with PE-related in-hospital death were older compared to patients with DVT-related in-hospital death. The median age at DVT-related hospital admission and at PE-related in-hospital death were lower in the second half of the study period compared to the first half. Male patients with DVT-related hospital admission had a lower median age than female patients (Table 1, Table S5-S7).

### Disease-specific incidence rate and proportion of hospital admissions

**Fig. 1:**
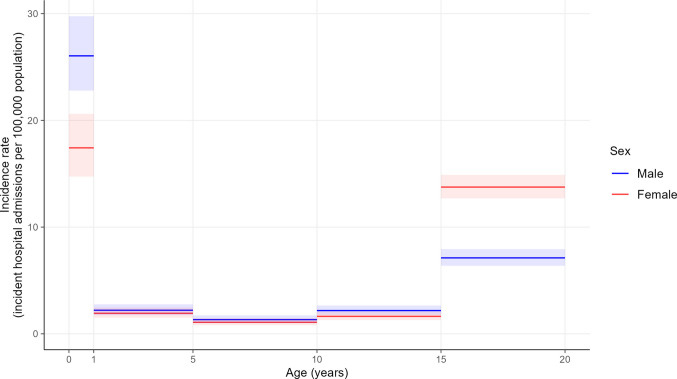
Venous thromboembolism (VTE)-related incidence rate (VTE cases per 100,000 children and adolescents per year) across age groups stratified by sex. The shaded area depicts the 95% confidence interval

**Fig. 2:**
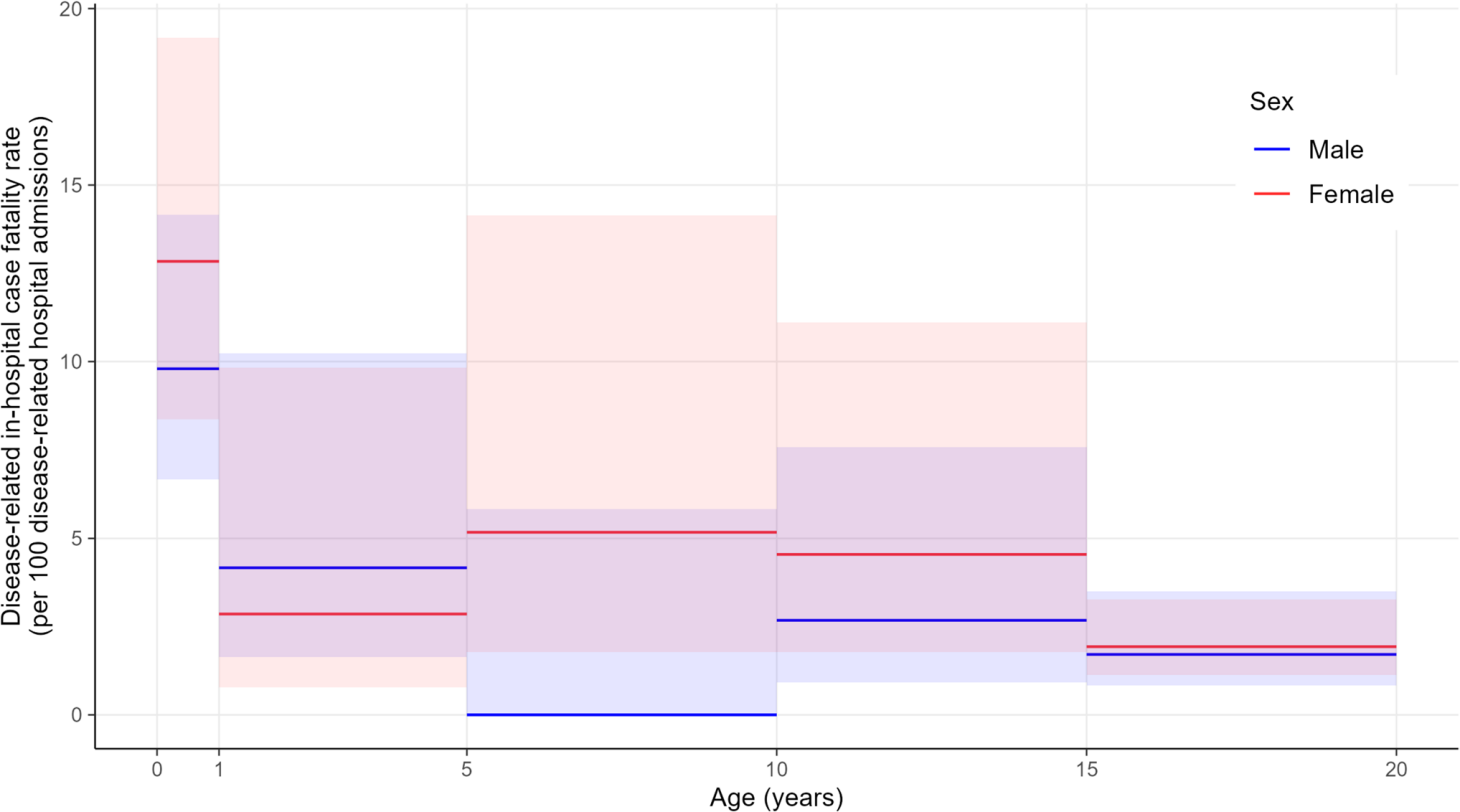
Venous thromboembolism (VTE)-related in-hospital case fatality rate (VTE-related deaths per 100 VTE-related hospital admissions) across age groups. The shaded area depicts the 95% confidence interval

The overall incidence rate of incident VTE events was 4.9 (95% CI: 4.7; 5.2) hospital admissions per 100,000 children and adolescents per year. While the VTE-related incidence rate was 5.9 (95% CI: 5.6; 6.1) VTE-related hospital admissions per 100,000 children and adolescents per year, corresponding to a proportion of 5.1 (95% CI: 4.9; 5.3) VTE-related hospital admissions per 10,000 hospital admissions. The incidence rate showed a bimodal age distribution, peaking in infants < 1 year and adolescents aged 15–19 years. Compared to PE, the incidence rate for DVT was more than 10 times higher among infants < 1 year and half as high among adolescents aged 15–19 years. Among infants aged < 1 year, the incidence rate for VTE and for DVT was higher among male patients. Among adolescents aged 15–19 years, female patients presented with a higher incidence rate for VTE, DVT, and PE (Figs. 1 and 2, Table S12-S14, and Figure S1-S3).

The proportion of VTE-related and PE-related hospital admissions increased quasi-exponentially across age groups. For DVT, the proportion increased linearly across age groups. The proportion of VTE-, PE-, and DVT-related hospital admissions was higher among female adolescents aged 15–19 years compared to males (Table S14 and Figure S3).

Only considering patients < 18 years, we found an incidence rate of incident VTE events of 3.9 (95% CI: 3.7; 4.1). The VTE-related incidence rate was 4.6 (95% CI: 4.4; 4.9) per 100,000 children and adolescents per year, corresponding to a proportion of 3.9 (95% CI: 3.7; 4.1) VTE-related hospital admissions per 10,000 hospital admissions. Age and sex trends were similar as in the primary analysis (Table S15-S17, Figure S4–S6).

### Disease-specific in-hospital case fatality rate

The overall VTE-related in-hospital case fatality rate was 4.0 (95% CI: 3.2; 5.0) deaths per 100 VTE-related hospitalizations. Infants < 1 year with PE presented with the highest rate of 42.9 (95% CI: 26.5; 60.9) deaths per 100 PE-related hospitalizations, with a decrease across older age groups. In contrast, the fatality rate among infants < 1 year with DVT was 8.2 (95% CI: 5.8; 11.5) deaths per 100 DVT-related hospitalizations, more than 4 times lower compared to patients with PE and decreased to fewer than 1 death per 100 DVT-related hospitalizations across older age groups (Fig. 2, Table S18, and Figure S7). Only considering patients < 18 years, the VTE-related in-hospital case fatality rate was 4.9 (95% CI: 3.9; 6.2) deaths per 100 VTE-related hospitalizations (Table S19, Figure S8).

High-risk features were identifiable in 592 (30.2% of all VTE-related hospital admissions) patients with VTE: 427 (37.3% of all DVT-related hospital admissions) patients with DVT and 165 (20.2% of all PE-related hospital admissions) patients with PE. Shock, cardiac arrest or resuscitation, and surgical thrombectomy were more prevalent among patients with PE compared to patients with DVT. Other high-risk features were more prevalent among patients with DVT compared to patients with PE. The prevalence was higher among infants and children aged 0–9 years for overall VTE, and both DVT and PE compared to adolescents aged 10–19 years. High-risk features were more prevalent among female patients with VTE and DVT compared to male patients, while no major differences were apparent in patients with PE (Table 2, Table S8–S11, and Table S20).

**Table 2:** In-hospital case fatality rate (CFR) stratified by age and presence of high-risk features

**Venous thromboembolism**									
	Overall (*N* = 1961)			0–9 years (*N* = 679)			10–19 years (*N* = 1282)		
	**Deaths**	**Prevalence**	**Case fatality rate**	**Deaths**	**Prevalence**	**Case fatality rate**	**Deaths**	**Prevalence**	**Case fatality rate**
Invasive ventilation	52	18.7%	18% (14.0; 22.8)	41	42.9%	14.1% (10.6; 18.6)	11	5.9%	14.5% (8.3; 24.1)
Shock	33	7.2%	41.3% (31.1; 52.2)	17	12.2%	20.5% (13.2; 30.4)	16	4.5%	27.6% (17.8; 40.2)
Cardiac arrest or resuscitation	34	4.7%	56.7% (44.1; 68.4)	23	9.7%	34.8% (24.5; 46.9)	11	2.1%	40.7% (24.5; 59.3)
Use of systemic thrombolysis	18	7.6%	19.4% (12.6; 28.5)	13	7.2%	26.5% (16.2; 40.3)	5	7.9%	5.0% (2.1; 11.1)
Use of vasopressors	34	10.3%	22.8% (16.8; 30.2)	26	22.8%	16.8% (11.7; 23.4)	8	3.7%	17.0% (8.9; 30.1)
ECMO	26	3.5%	57.8% (43.3; 71.0)	13	6.2%	31.0% (19.1; 46.0)	13	2.0%	50.0% (32.1; 67.9)
Surgical thrombectomy	2	0.7%	28.6% (8.2; 64.1)	1	1.0%	14.3% (2.6; 51.3)	1	0.5%	16.7% (3.0; 56.4)
Any of the above	70	30.2%	16.4% (13.2; 20.2)	49	54.9%	13.1% (10.1; 16.9)	21	17.1%	9.6% (6.4; 14.2)
None of the above	9	69.8%	0.7% (0.3; 1.2)	3	45.1%	1.0% (0.3; 2.8)	6	82.9%	0.6% (0.3; 1.2)
Catheter-directed treatment	4	3.9%	8.0% (3.2; 18.8)	2	1.3%	22.2% (6.3; 54.7)	2	5.3%	2.9% (0.8; 10.1)
Placement of an inferior vena cava filter	0	1.6%	0	0	0.1%	0	0	2.4%	0
**Pulmonary embolism (with or without deep vein thrombosis)**									
	Overall (*N* = 815)			0–9 years (*N* = 81)			10–19 years (*N* = 734)		
	Deaths	Prevalence	Case fatality rate	Deaths	Prevalence	Case fatality rate	Deaths	Prevalence	Case fatality rate
Invasive ventilation	20	4.0%	25.6% (17.3; 36.3)	13	18.5%	44.8% (28.4; 11.7)	7	2.5%	14.3% (7.1; 7.3)
Shock	17	7.5%	27.9% (18.2; 40.2)	6	19.8%	37.5% (18.5; 19.4)	11	6.1%	24.4% (14.2; 7.9)
Cardiac arrest or resuscitation	15	6.5%	45.5% (29.8; 62.0)	6	23.5%	40.0% (19.8; 20.4)	9	4.6%	50.0% (29.0; 17.6)
Use of systemic thrombolysis	13	7.0%	22.8% (13.8; 35.2)	8	13.6%	72.7% (43.4; 25.9)	5	6.3%	10.9% (4.7; 7.7)
Use of vasopressors	13	9.6%	24.5% (14.9; 37.6)	7	35.8%	36.8% (19.1; 16.8)	6	6.7%	17.6% (8.3; 10.2)
ECMO	11	2.8%	47.8% (29.2; 67.0)	3	9.9%	37.5% (13.7; 32.4)	8	2.0%	53.3% (30.1; 20.4)
Surgical thrombectomy	2	0.7%	33.3% (9.7; 70.0)	1	2.5%	50.0% (9.5; 65.8)	1	0.5%	25.0% (4.6; 49.0)
Any of the above	30	20.2%	18.2% (13.0; 24.8)	15	53.1%	34.9% (22.4; 8.2)	15	16.6%	12.3% (7.6; 3.1)
None of the above	5	79.8%	0.8% (0.3; 1.8)	0	46.9%	0	5	83.4%	0.8% (0.3; 0.6)
Catheter-directed treatment	4	3.3%	14.8% (5.9; 32.5)	2	6.2%	40.0% (11.8; 76.9)	2	3.0%	9.1% (2.5; 27.8)
Placement of an inferior vena cava filter	0	2.7%	0	0	0	0	0	3.0%	0
**Deep vein thrombosis (excluding cases with concomitant pulmonary embolism diagnosis)**									
	Overall (*N* = 1146)			0–9 years (*N* = 598)			10–19 years (*N* = 548)		
	Deaths	Prevalence	Case fatality rate	Deaths	Prevalence	Case fatality rate	Deaths	Prevalence	Case fatality rate
Invasive ventilation	32	25.2%	11.1% (8.0; 15.2)	28	43.8%	10.7% (7.5; 15.0)	4	4.9%	14.8% (5.9; 32.5)
Shock	16	7.0%	20% (12.7; 30.0)	11	11.2%	16.4% (9.4; 27.1)	5	2.4%	38.5% (17.7; 64.5)
Cardiac arrest or resuscitation	19	5.2%	31.7% (21.3; 44.2)	17	8.5%	33.3% (22.0; 47)	2	1.6%	22.2% (6.3; 54.7)
Use of systemic thrombolysis	5	8.1%	5.4% (2.3; 12.0)	5	6.4%	13.2% (5.8; 27.3)	0	10.0%	0
Use of vasopressors	21	13.0%	14.1% (9.4; 20.6)	19	22.7%	14.0% (9.1; 20.8)	2	2.4%	15.4% (4.3; 42.2)
ECMO	15	3.9%	33.3% (21.4; 47.9)	10	5.7%	29.4% (16.8; 46.2)	5	2.0%	45.5% (21.3; 72.0)
Surgical thrombectomy	0	0.6%	0	0	0.8%	0	0	0.4%	0
Any of the above	40	37.3%	9.4% (7.0; 12.5)	34	55.2%	10.3% (7.5; 14.1)	6	17.7%	6.2% (2.9; 12.8)
None of the above	4	62.7%	0.6% (0.2; 1.4)	3	44.8%	1.1% (0.4; 3.2)	1	82.3%	0.2% (0; 1.2)
Catheter-directed treatment	0	4.4%	0	0	0.7%	0	0	8.4%	0
Placement of an inferior vena cava filter	0	0.9%	0	0	0.2%	0	0	1.6%	0

*ECMO* extracorporeal membrane oxygenation

The in-hospital case fatality rate was 16.4% (95% CI: 13.2; 20.2) among patients with VTE with high-risk features compared to 0.7% (95% CI: 0.3; 1.2) among VTE patients without high-risk features. For patients with PE the rates were 17.1% (95% CI: 12.0–23.7) and 0.7% (95% CI: 0.3–1.8), respectively. For patients with DVT the rates were lower: 9.4% (95% CI: 7.0; 12.5) and 0.6% (95% CI: 0.2; 1.4), respectively. For most high-risk features, fatality rates were higher in infants and children aged 0–9 years compared to adolescents aged 10–19 years. Sex differences in patients with VTE were present for all high-risk features: In patients with PE, such differences were observed for invasive ventilation and cardiac arrest or resuscitation. In patients with DVT, differences were observed for shock, use of systemic thrombolysis, and ECMO (Table 2 and Table S20)*.*

### Therapeutic procedures

The most common therapeutic procedures were blood product transfusions (*n* = 515), enteral or parenteral nutrition (*n* = 462), and invasive ventilation (*n* = 367). Central-venous catheters were mentioned in 312 (15.9%) patients. Systemic thrombolysis (*n* = 150; 7.6%), catheter-directed treatment (*n* = 77; 3.9%), and inferior vena cava filters (*n* = 32; 1.6%) were less frequently used. No major differences between patients with PE and DVT were observed. The absolute number and prevalence of therapeutic procedures increased from the first to the second half of the study period (Table 3). No major sex differences in the frequency of usage of advanced treatment options were observed (Table S21-S24)*.*

**Table 3:** Therapeutic procedures performed in patients with venous thromboembolism stratified by age

Characteristic	Venous thromboembolism			Pulmonary embolism (with or without deep vein thrombosis)			Deep vein thrombosis (excluding cases with concomitant pulmonary embolism diagnosis)		
	Overall, *N* = 1961	0 to 9 years old, *N* = 679	10 to 19 years old, *N* = 1282	Overall, *N* = 815	0 to 9 years old, *N* = 81	10–19 years old, *N* = 734	Overall, *N* = 1146	0 to 9 years old, *N* = 598	10–19 years old, *N* = 548
Blood product transfusion, *n* (%)	515 (26.3%)	353 (52.0%)	162 (12.6%)	150 (18.4%)	48 (59.3%)	102 (13.9%)	365 (31.8%)	305 (51.0%)	60 (10.9%)
Transfusion of erythrocytes	473 (24.1%)	326 (48.0%)	147 (11.5%)	139 (17.1%)	46 (56.8%)	93 (12.7%)	334 (29.1%)	280 (46.8%)	54 (9.9%)
Transfusion of plasma	275 (14.0%)	214 (31.5%)	61 (4.8%)	73 (9.0%)	36 (44.4%)	37 (5.0%)	202 (17.6%)	178 (29.8%)	24 (4.4%)
Transfusion of thrombocytes	238 (12.1%)	183 (27.0%)	55 (4.3%)	64 (7.9%)	30 (37.0%)	34 (4.6%)	174 (15.2%)	153 (25.6%)	21 (3.8%)
Transfusion of coagulation factors	183 (9.3%)	142 (20.9%)	41 (3.2%)	42 (5.2%)	20 (24.7%)	22 (3.0%)	141 (12.3%)	122 (20.4%)	19 (3.5%)
Invasive ventilation, *n* (%)	367 (18.7%)	291 (42.9%)	76 (5.9%)	78 (9.6%)	29 (35.8%)	49 (6.7%)	289 (25.2%)	262 (43.8%)	27 (4.9%)
Central venous catheter, *n*/*N* (%)	312 (15.9%)	231 (34.0%)	81 (6.3%)	82 (10.1%)	27 (33.3%)	55 (7.5%)	230 (20.1%)	204 (34.1%)	26 (4.7%)
Usage of a heart–lung machine (excl. ECMO), *n* (%)	198 (10.1%)	163 (24.0%)	35 (2.7%)	54 (6.6%)	30 (37.0%)	24 (3.3%)	144 (12.6%)	133 (22.2%)	11 (2.0%)
Use of systemic thrombolysis, *n* (%)	150 (7.6%)	49 (7.2%)	101 (7.9%)	57 (7.0%)	11 (13.6%)	46 (6.3%)	93 (8.1%)	38 (6.4%)	55 (10.0%)
Right-heart catheter, *n* (%)	103 (5.3%)	88 (13.0%)	15 (1.2%)	23 (2.8%)	18 (22.2%)	5 (0.7%)	80 (7.0%)	70 (11.7%)	10 (1.8%)
Catheter-directed treatment, *n* (%)	77 (3.9%)	9 (1.3%)	68 (5.3%)	27 (3.3%)	5 (6.2%)	22 (3.0%)	50 (4.4%)	4 (0.7%)	46 (8.4%)
Intervention at abdominal veins, *n* (%)	40 (2.0%)	22 (3.2%)	18 (1.4%)	18 (2.2%)	2 (2.5%)	16 (2.2%)	22 (1.9%)	20 (3.3%)	2 (0.4%)
Extracorporeal membrane oxygenation, *n* (%)	68 (3.5%)	42 (6.2%)	26 (2.0%)	23 (2.8%)	8 (9.9%)	15 (2.0%)	45 (3.9%)	34 (5.7%)	11 (2.0%)
Hemodialysis, *n* (%)	64 (3.3%)	35 (5.2%)	29 (2.3%)	22 (2.7%)	5 (6.2%)	17 (2.3%)	42 (3.7%)	30 (5.0%)	12 (2.2%)
Intervention at pulmonary vessels, *n* (%)	46 (2.3%)	41 (6.0%)	5 (0.4%)	14 (1.7%)	11 (13.6%)	3 (0.4%)	32 (2.8%)	30 (5.0%)	2 (0.4%)
Inferior vena cava filter placement, *n* (%)	32 (1.6%)	1 (0.1%)	31 (2.4%)	22 (2.7%)	0	22 (3.0%)	10 (0.9%)	1 (0.2%)	9 (1.6%)
Surgical thrombectomy, *n* (%)	13 (0.7%)	7 (1.0%)	6 (0.5%)	6 (0.7%)	2 (2.5%)	4 (0.5%)	7 (0.6%)	5 (0.8%)	2 (0.4%)

Catheter-directed treatment was performed in five (9.6%) infants and children with PE aged 0–9 years. Of those, four were catheter-directed thrombectomy, and one was local thrombolysis. All of them presented with severe comorbidities and were admitted to an ICU, and two (40%) died during the hospital stay. Four (0.7%) infants and children with DVT aged 0–9 years were treated with catheter-directed treatment: three with local thrombolysis and two with catheter-directed recanalization. Of these four, two were admitted to ICU, and none died (Table 2).

Among children aged 10–19 years, catheter-directed treatment was performed in 46 (8.4%) patients with DVT and in 22 (3.0%) patients with PE. High-risk features were present in 33 (71.7%) of the patients with DVT and catheter-directed treatment and in 17 (77.3%) of the patients with PE and catheter-directed treatment, respectively. ICU admission was recorded in 4 (8.7%) of the patients with DVT and catheter-directed treatment and in 9 (40.1%) of the patients with PE and catheter-directed treatment, respectively. Of the patients with PE, 2 (9.1%) died; none of the patients with DVT died (Table 2).

### Comorbidities

The most prevalent comorbidities were injury or trauma (*n* = 762), respiratory diseases (*n* = 652), and infectious diseases (*n* = 535). Coagulation disturbances or thrombophilia (*n* = 278) were less prevalent. Comorbidities were generally more prevalent in patients with DVT compared to PE, and in the second half (vs. first half) of the study period. The prevalence of comorbidities was higher among infants and children aged 0–9 years than adolescents aged 10–19 years. No major sex differences were observed (Table S25-S28)*.*

### Length of hospitalization and stay in an intensive care unit

The median LOS was 8 (Q1–Q3: 3–24) days for all VTE-related hospitalizations. Patients with PE were hospitalized for a shorter median duration of 6 (Q1–Q3: 3–15) days compared to 11 (Q1–Q3: 3–32) days for those with DVT. The LOS decreased across age groups (Table S29, Figure S9). No substantial sex and time differences were evident across age groups for both PE and DVT (Table S8–S11)*.* Only considering patients < 18 years, the median LOS was 11 (Q1–Q3: 4–32) days for all VTE-related hospitalizations (Table S30, Figure S10).

**Fig. 3:**
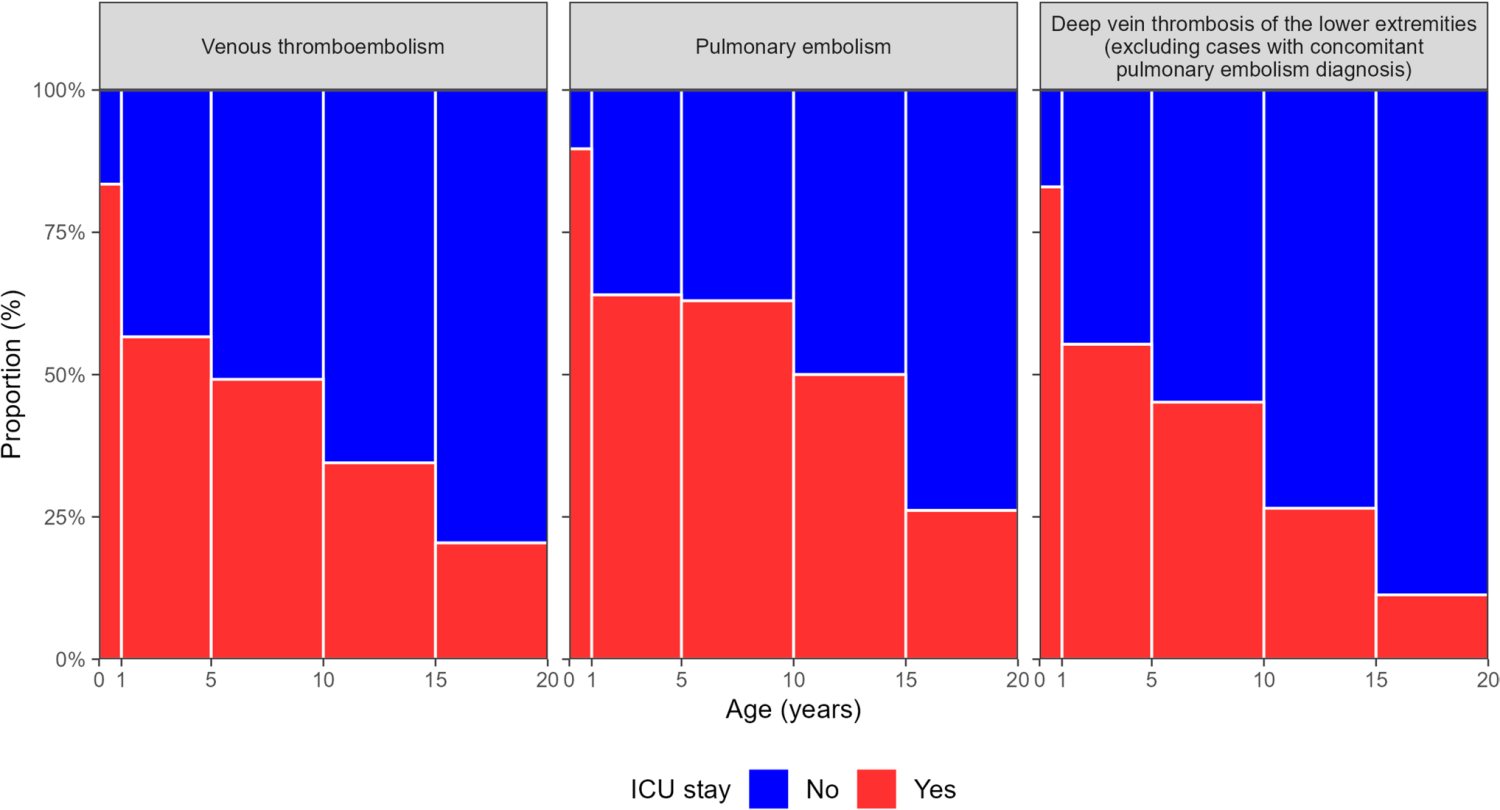
Proportion of intensive care unit (ICU) admissions for patients with venous thromboembolism, pulmonary embolism, and deep vein thrombosis across age groups

Overall, 771 (39.3%) patients were admitted to an ICU. The proportion was higher among patients with DVT (vs. PE). Infants and children aged 0–9 years were more frequently admitted to the ICU compared to adolescents aged 10–19 years. Notably, the frequency of ICU admission in the second half of the study period was almost double the frequency in the first half. The median duration of ICU stay was 210 (Q1–Q3: 59–696) hours. LOS in the ICU was longer among patients with DVT than among patients with PE and decreased with increasing age. In the second half (vs. first half) of the study period, the duration was consistently longer. The duration was shorter for males (vs. females) with PE and females (vs. males) with DVT (Table S8–S11, Fig. 3, and Figure S11)*.*

### Clinical outcomes

In sex-adjusted logistical regression models, mechanical ventilation, use of vasopressors, use of systemic thrombolysis, ECMO, surgical thrombectomy, and presence of any high-risk features were associated with death, intracranial hemorrhage, non-intracranial hemorrhage, and admission to an ICU. No outcome variables were found to be associated with catheter-directed treatment or PE (reference: patients with DVT). Placement of an inferior vena cava filter was associated with hemorrhage (not including intracranial hemorrhage) and admission to an ICU. The association persisted after stratification into infants and children aged 0–9 years and adolescents aged 10–19 years. Among adolescents, PE (vs. DVT) was associated with admission to an ICU (Table S31-S3)*.*

### Sensitivity analysis

Only considering patients with acute PE as the primary diagnosis, we found similar age trends for the incidence rate as for PE as the primary or concomitant diagnosis. For DVT as the primary diagnosis, the peak among infants < 1 year was not apparent (Figure S12). Similarly, the trends across age groups persisted in the proportion of disease-related hospital admissions (Figure S13).

## Discussion

This study presents a comprehensive overview of measures of occurrence of VTE, acute PE, and DVT of the lower extremity-related hospital admissions in Switzerland in children and adolescents covering the period 2004 to 2023. Additionally, we provided an analysis of the most frequently performed therapeutic procedures. We showed that advanced treatment options presented with a substantial risk of in-hospital death. Catheter-directed treatment was increasingly used over time.

Overall, we found an incidence rate of 4.9 incident hospital admissions per 100,000 children and adolescents per year for VTE, of 2.1 incident hospital admissions per 100,000 children and adolescents per year for PE, and of 2.8 incident admissions per 100,000 children and adolescents per year for DVT. In the USA, for the years 2016 and 2019, a slightly higher annualized incidence rate of acute PE-related hospitalizations of 3.5 hospital admissions per 100,000 children and adolescents has been described [1]. In Canada, from 1990 through 1992, the incidence rate of DVT has been estimated at a lower rate of 0.7 per 100,000 children and adolescents, while in the USA, from 1979 through 2001, the rate was 4.2 per 100,000 children and adolescents [3, 4]. For VTE overall and for PE specifically, an increasing time trend has been described among all age groups, including children and adolescents[7, 8, 14]. Time trends for DVT in this age group are unknown and should be the topic of future research. Interestingly, the incidence rate of DVT was higher than that of PE among infants and children aged 0–9 years, but lower than that of PE among adolescents aged 15–19 years. This might partly be explained by our dataset only including hospitalized patients. Physicians may perceive DVT events in this age group as less severe and less prone to severe complications than PE events and thus not admit patients to a hospital [21, 22]. In contrast, we found a higher risk of ICU admission among patients with DVT than among patients with PE and a longer LOS in ICU. This might be linked to DVT being diagnosed in patients with prolonged hospitalization, a known risk factor [23], but also due to a paucity of evidence on the optimal approach to thromboprophylaxis. Additionally, placement of central venous catheters—a risk factor for hospital-acquired VTE especially among infants aged 0 years—was common [4]. Further studies including outpatient and inpatient diagnoses of DVT are needed to generate robust estimates of the incidence rate over the whole spectrum of disease severity.

Regarding the proportion of hospital admissions, we found 2.1 PE-related hospital admissions per 10,000 hospital admissions and 3.0 DVT-related hospital admissions per 10,000 hospital admissions. For acute PE, we found a fivefold lower proportion compared to the USA [1]. In regard to DVT, we found a 4- to fivefold lower proportion of hospital admissions compared to Canada [4]. Considering the comparable incidence rates, this difference may be due to a lower threshold of hospitalization in Switzerland compared to the USA and Canada but also due to changes in medical practice over the past 20–30 years with improved PE diagnosis. This is in line with findings from studies in adults, indicating a progressive increase in PE incidence over the past decades [24]. Furthermore, our study only included patients with DVT of the lower extremities whereas the Canadian study additionally included DVT of the upper extremities [4].

Systemic thrombolysis was performed more frequently than catheter-directed interventions in this age group and more than twice as frequently as in adult patients with acute PE in Switzerland [14]. Interestingly, the frequency of placement of an inferior vena cava filter was substantially higher among children and adolescents compared to adults with PE [25]. Evidence on interventional treatment among children and adolescents is sparse. In fact, most trials on interventional treatment strategies for PE exclude patients younger than 18 [26, 27]. The scarcity of evidence is highlighted by the European guidelines not addressing the topic [9]. Therefore, future trials on the efficacy and safety of catheter-directed treatment should include children and adolescents to identify and establish optimal treatment regimens in this age group.

Catheter-directed treatment compared to systemic thrombolysis presented with a lower in-hospital mortality rate for the overall cohort. This higher rate found for systemic thrombolysis might be due to sicker patients being considered for this treatment option. For patients with PE, the difference was mainly apparent among adolescents aged 10–19 years. Furthermore, catheter-directed treatment was not associated with death, hemorrhage, and ICU admission in logistic regression analyses. At the same time, the use of systemic thrombolysis was associated with all clinical outcomes studied. Additionally, no patients with DVT treated with catheter-directed treatment died during the index hospitalization. Therefore, our results suggest that catheter-directed treatment is increasingly used particularly in children and adolescents aged 0–19 years with DVT and patients with acute PE aged 10–19 years, with no evidence of safety or efficacy issues. However, departments of interventional radiology are not generally available in Swiss children’s hospitals. Nevertheless, further research is needed to investigate the safety and feasibility of catheter-directed treatment in this patient population.

Patients presenting with high-risk features showed a substantially higher in-hospital case fatality rate compared to patients without high-risk features. This is most likely because these may indicate severe disease, represent indications for systemic thrombolysis, and thus are associated with a higher mortality [9].

Overall, 79 deaths were related to VTE (44 to DVT, 35 to PE) with higher fatalities recorded in female patients. For PE, the fatality rate was highest among infants < 1 year and among adolescents aged 15 to 19 years. For DVT, the fatality was highest among infants < 1 year. This high rate is likely explained by severe underlying disorders requiring hospitalization, a possible risk factor for acute PE. In our prior study, however, our data suggested that (severe) acute PE may be independently associated with a higher death rate which may depend on the limited treatment options of acute PE in this age group of patients [1]. In Switzerland, the most frequent causes of death among the population aged 0–24 years are accidents and cancer [28]. All in all, VTE remains a rare condition and a rare cause of death among children and adolescents in Switzerland.

### Limitations

This study carries the typical limitations of research conducted with administrative and/or survival registration data, and due to its observational design, no causal inferences can be made, particularly regarding treatment efficacy and effectiveness. First, the Swiss medical statistics only includes in-patient hospitalizations; thus patients discharged without formally being admitted (usually at least one overnight stay) were not included in our analysis. This could have led to an underestimation of the incidence rate observed and to a higher case fatality rate. In particular, this might explain that the incidence rate of DVT among adolescents aged 15–19 years was lower than the rate of PE [29]. Regarding the case fatality rate, higher rates may have been expected if deaths after discharge (e.g., 7 days or 30 days) had been included. However, the vast majority of PE-related deaths occur within the first 5 days after diagnosis, and thus likely occur within the index hospitalization [30]. Second, the ICD-10-GM coding is subject to inaccuracies and misclassification, particularly regarding underreporting of less severe cases or, vice versa, reporting of prevalent (and not incident) cases. Currently, no studies on the reliability of ICD-10-GM coding in Switzerland are available; in other countries, validation studies for ICD-10 codes of DVT and PE are available [20, 31]. Additionally, as the data were anonymized, no manual accuracy checks of ICD-10-GM and CHOP codes were possible. However, Switzerland employs a structured multi-level validation system as both ICD-10-GM and CHOP codes are relevant for reimbursement, including physician coding oversight, institutional audits, and insurance reviews, which support the reliability of both coding systems. The direction of potential error cannot be determined, as it depends on both the measure of occurrence under study and whether misclassification or underreporting is predominant. Third, due to the nature of the dataset, no analysis of the anticoagulant treatment was feasible.

## Conclusion

In conclusion, VTE among children and adolescents is a rare disease, with an incidence rate of 4.9 incident hospitalizations per 100,000 children and adolescents per year in Switzerland, corresponding to a proportion of 5.1 VTE-related hospitalizations per 10,000 hospital admissions in this age group. Age-sex differences in the incidence rate, proportion of hospital admissions, and in-hospital case fatality rate were observed. Catheter-directed interventional treatment strategies and systemic thrombolysis presented a risk of in-hospital death of 8.0% and 19.4%, respectively.

## Supplementary Information

Below is the link to the electronic supplementary material.

Supplementary file 1 (DOCX 3.86 MB)

## Data Availability

No datasets were generated or analysed during the current study.

## Abbreviations

CHOP: Swiss classification of surgical interventions
DVT: Deep vein thrombosis
ICD-10-GM: International Classification of Disease, 10th revision with German modifications
ICU: Intensive care unit
LOS: Length of stay
PE: Pulmonary embolism
VTE: Venous thromboembolism
