# Supplementary material for: Burden and management of venous thromboembolism in children and adolescents (2004–2023): a Swiss nationwide epidemiological study
Source: Eur J Pediatr. 2025 Nov 17;184(12):768. doi: 10.1007/s00431-025-06598-4 (PMC12628491; doi:10.1007/s00431-025-06598-4)
Supplement: Supplementary file 1 — (DOCX 3.86 MB) [file 431_2025_6598_MOESM1_ESM.docx]

Online Data Supplement
__________________________________________________________________________________________

**Table of Contents**

[Table S1. Definition of pulmonary embolism and deep vein thrombosis based on ICD-10-GM codes. 5](#_Toc211586045)

[Table S2. Definition of comorbidities based on ICD-10-GM codes. 6](#_Toc211586046)

[Table S3. Definition of high-risk features based on ICD-10-GM codes and the Swiss classification of surgical procedures (CHOP). 9](#_Toc211586047)

[Table S4. Definition of therapeutic procedures based on Swiss classification of surgical procedures (CHOP) codes. 11](#_Toc211586048)

[Table S5. Overview of incident venous thromboembolism cases, and venous thromboembolism related deaths per age group, stratified by sex. 14](#_Toc211586049)

[Table S6. Overview of venous thromboembolism (VTE) cases, and VTE-related deaths per age group, stratified by sex and time period. 15](#_Toc211586050)

[Table S7. Overview of the pulmonary embolism (PE) and deep vein thrombosis (DVT) of the lower extremity cases, and PE-related and DVT-related deaths per age group, stratified by sex and time period. 16](#_Toc211586051)

[Table S8. Overview of high-risk features mentioned in venous thromboembolism cases, intensive care unit (ICU) stay, and length of hospitalization, stratified by age and time period. 17](#_Toc211586052)

[Table S9. Overview of high-risk features mentioned in pulmonary embolism (PE) and deep vein thrombosis (DVT) cases, intensive care unit (ICU) stay, and length of hospitalization, stratified by age and time period. 18](#_Toc211586053)

[Table S10. Overview of high-risk features mentioned in venous thromboembolism cases, intensive care unit (ICU) stay, and length of hospitalization, stratified by age and sex. 19](#_Toc211586054)

[Table S11. Overview of high-risk features mentioned in pulmonary embolism and deep vein thrombosis cases, intensive care unit (ICU) stay, and length of hospitalization, stratified by age and sex. 20](#_Toc211586055)

[Table S12. Incidence rate (IR) of incident hospital admissions, per 100,000 children and adolescents per year stratified by age and sex. 21](#_Toc211586056)

[Table S13. Incidence rate (IR) of disease-related hospital admissions, per 100,000 children and adolescents per year stratified by age and sex. 22](#_Toc211586057)

[Table S14. Proportion of hospitalizations (PH) of disease-related hospital admissions, per 10,000 hospital admissions stratified by age and sex. 23](#_Toc211586058)

[Table S15. Incidence rate (IR) of incident hospital admissions, per 100,000 children and adolescents stratified by clinically selected age groups and sex. 24](#_Toc211586059)

[Table S16. Incidence rate (IR) of disease-related hospital admissions, per 100,000 children and adolescents stratified by clinically selected age groups and sex. 25](#_Toc211586060)

[Table S17. Proportion of hospitalizations (PH) of disease-related hospital admissions, per 10,000 hospital admissions stratified by clinically selected age groups and sex. 26](#_Toc211586061)

[Table S18. In-hospital case fatality rate (CFR), per 100 disease-related hospital admissions stratified by age. 27](#_Toc211586062)

[Table S19. In-hospital case fatality rate (CFR), per 100 disease-related hospital admissions stratified by clinically selected age groups. 28](#_Toc211586063)

[Table S20. In-hospital case fatality rate (CFR) stratified by sex and presence of high-risk features. 29](#_Toc211586064)

[Table S21. Therapeutic procedures performed in patients with venous thromboembolism stratified by age and time period. 31](#_Toc211586065)

[Table S22. Therapeutic procedures performed in patients with pulmonary embolism and deep vein thrombosis stratified by age and time period. 32](#_Toc211586066)

[Table S23. Therapeutic procedures performed in patients with venous thromboembolism stratified by age and sex. 33](#_Toc211586067)

[Table S24. Therapeutic procedures performed in patients with pulmonary embolism stratified by age and sex. 34](#_Toc211586068)

[Table S25. Comorbidities in patients with venous thromboembolism stratified by age and time period. 35](#_Toc211586069)

[Table S26. Comorbidities in patients with pulmonary embolism and deep vein thrombosis stratified by age and time period. 37](#_Toc211586070)

[Table S27. Comorbidities in patients with venous thromboembolism stratified by age and sex. 40](#_Toc211586071)

[Table S28. Comorbidities in patients with pulmonary embolism and deep vein thrombosis stratified by age and sex. 42](#_Toc211586072)

[Table S29. Length of hospitalization ([Q1-Q3] days) stratified by age and sex. 45](#_Toc211586073)

[Table S30. Length of hospitalization ([Q1-Q3] days) stratified by clinically selected age groups and sex. 46](#_Toc211586074)

[Table S31. Univariable logistical regression models for selected clinical outcomes. 47](#_Toc211586075)

[Table S32. Multivariable logistical regression models adjusted for sex for selected clinical outcomes. 49](#_Toc211586076)

[Figure S1. Pulmonary embolism (PE)-related and deep vein thrombosis (DVT)-related incidence rate (PE / DVT incident hospital admissions per 100,000 children and adolescents per year) across age groups stratified by sex. 51](#_Toc211586077)

[Figure S2. Venous thromboembolism (VTE)-related, pulmonary embolism (PE)-related and deep vein thrombosis (DVT)-related incidence rate (VTE / PE / DVT-related hospital admissions per 100,000 children and adolescents per year) across age groups stratified by sex. 52](#_Toc211586078)

[Figure S3. Proportion of venous thromboembolism (VTE)-related, pulmonary embolism (PE)-related, and deep vein thrombosis (DVT)-related hospitalizations out of all hospitalizations (VTE / PE / DVT-related hospital admissions per 10,000 hospital admissions) across age groups in male and female patients. 53](#_Toc211586079)

[Figure S4. Venous thromboembolism (VTE)-related, pulmonary embolism (PE)-related and deep vein thrombosis (DVT)-related incidence rate (VTE / PE / DVT incident hospital admissions per 100,000 children and adolescents per year) across clinically selected age groups stratified by sex. 54](#_Toc211586080)

[Figure S5. Venous thromboembolism (VTE)-related, pulmonary embolism (PE)-related and deep vein thrombosis (DVT)-related incidence rate (VTE / PE / DVT-related hospital admissions per 100,000 children and adolescents per year) across clinically selected age groups stratified by sex. 55](#_Toc211586081)

[Figure S6. Proportion of venous thromboembolism (VTE)-related, pulmonary embolism (PE)-related, and deep vein thrombosis (DVT)-related hospitalizations out of all hospitalizations (VTE / PE / DVT-related hospital admissions per 10,000 hospital admissions) across clinically selected age groups in male and female patients. 56](#_Toc211586082)

[Figure S7. Pulmonary embolism (PE)-related and deep vein thrombosis (DVT)-related in-hospital case fatality rate (PE / DVT-related deaths per 100 PE / DVT-related hospital admissions) across age groups. 57](#_Toc211586083)

[Figure S8. Venous thromboembolism (VTE)-related, pulmonary embolism (PE)-related and deep vein thrombosis (DVT)-related in-hospital case fatality rate (PE / DVT-related deaths per 100 PE / DVT-related hospital admissions) across clinically selected age groups. 58](#_Toc211586084)

[Figure S9. Median length of hospitalization (days) for patients with venous thromboembolism, pulmonary embolism, and deep vein thrombosis across age groups stratified by sex. 59](#_Toc211586085)

[Figure S10. Median length of hospitalization (days) for patients with venous thromboembolism, pulmonary embolism, and deep vein thrombosis across clinically selected age groups stratified by sex. 60](#_Toc211586086)

[Figure S11. Proportion of intensive care unit (ICU) admissions for patients with venous thromboembolism, pulmonary embolism, and deep vein thrombosis across clinically selected age groups. 61](#_Toc211586087)

[Figure S12. Venous thromboembolism (VTE)-related, pulmonary embolism (PE)-related, and deep vein thrombosis (DVT)-related incidence rate (VTE / PE / DVT incident hospital admission per 100,000 children and adolescents per year) for VTE / PE / DVT as primary diagnosis at discharge across age groups stratified by sex. 62](#_Toc211586088)

[Figure S13. Proportion of venous thromboembolism (VTE)-related, pulmonary embolism (PE)-related, and deep vein thrombosis (DVT)-related hospitalizations (VTE / PE / DVT-related hospital admissions per 10,000 hospital admissions) for VTE / PE / DVT as primary diagnosis at discharge across age groups in male and female patients. 63](#_Toc211586089)

# Table S1. Definition of pulmonary embolism and deep vein thrombosis based on ICD-10-GM codes.

| **ICD-10-GM Code** | **Description** | **Primary diagnosis** | **Primary or concomitant diagnosis** | **Underlying or secondary cause of death** |
| --- | --- | --- | --- | --- |
| I26.x | Pulmonary embolism | 426 | 804 | 35 |
| O88.2 | Obstetric thromboembolism | 4 | 11 | 0 |
| I80.1 | Phlebitis and thrombophlebitis of femoral vein | 48 | 349 | 21 |
|  | Excluding cases without concomitant PE diagnosis | 41 | 301 | 19 |
| I80.2 | Phlebitis and thrombophlebitis of other deep vessels of lower extremities | 259 | 714 | 20 |
|  | Excluding cases without concomitant PE diagnosis | 231 | 589 | 14 |
| I80.3 | Phlebitis and thrombophlebitis of lower extremities, unspecified | 16 | 79 | 1 |
|  | Excluding cases without concomitant PE diagnosis | 15 | 71 | 1 |
| I82.2 | Thrombosis of the vena cava | 44 | 407 | 23 |
|  | Thrombosis of the vena cava without concomitant PE diagnosis | 35 | 366 | 17 |

# Table S2. Definition of comorbidities based on ICD-10-GM codes.

| **Condition** | **ICD-10-GM Codes** | **Definition** |
| --- | --- | --- |
| Infectious diseases | A.x, B.x | Certain infectious and parasitic diseases |
|  | R65.x | Symptoms and signs specifically associated with systemic inflammation and infection |
| Sepsis | A40.x | Streptococcal sepsis |
|  | A41.x | Other sepsis |
| Cancer | C.x | Malignant neoplasms |
|  | D00.x to D09.x | Non-benign in situ neoplasms |
|  | D37.x to D45.x | Neoplasms of uncertain behavior, polycythemia vera |
|  | Z85.x | Personal history of malignant neoplasm* |
| Hemorrhage | D62.x | Acute posthemorrhagic anemia |
|  | D68.3 | Hemorrhagic disorder due to circulating anticoagulants |
|  | D69.9 | Hemorrhagic condition, unspecified |
|  | H11.3x | Conjunctival hemorrhage |
|  | H21.0 | Hyphema |
|  | H31.3 | Choroidal hemorrhage and rupture |
|  | H35.6x | Retinal hemorrhage |
|  | H43.1x | Vitreous hemorrhage |
|  | H45.0x | Vitreous hemorrhage in diseases classified elsewhere |
|  | I31.2 | Hemopericardium, not elsewhere classified |
|  | I60.x to I62.x | Subarachnoid or intracerebral bleeding |
|  | J94.2 | Hemothorax |
|  | K25.x to K28.x | Gastrointestinal ulcer with hemorrhage or hemorrhagic gastritis |
|  | K62.5 | Hemorrhage of anus and rectum |
|  | K66.1 | Hemoperitoneum |
|  | K92.0x | Hematemesis |
|  | K92.1x | Melena |
|  | K92.2x | Gastrointestinal hemorrhage, unspecified |
|  | M25.0 | Hemarthrosis |
|  | N02.x | Hematuria |
|  | N93.9 | Abnormal uterine and vaginal bleeding, unspecified |
|  | N95.0 | Postmenopausal bleeding |
|  | R04.x | Hemorrhage from respiratory passages |
|  | R31.x | Unspecified hematuria |
|  | R58.x | Hemorrhage, not elsewhere classified |
|  | S06.x | Epidural hemorrhage; Traumatic subdural or subarachnoid hemorrhage |
|  | S27.1 | Traumatic hemothorax |
|  | T79.2 | Traumatic secondary and recurrent hemorrhage |
| Coagulation disturbances or thrombophilia | D68 | Coagulation disturbances or thrombophilia |
|  | D68.1, D68.2 | Hereditary deficiency of clotting factors |
|  | D68.4 | Secondary thrombophilia |
|  | D68.5 | Primary thrombophilia |
| Hyposplenism | D73.0 |  |
| Diabetes mellitus | E10 | Type 1 diabetes mellitus |
|  | E11 | Type 2 diabetes mellitus |
|  | E12 | Malnutrition-related diabetes mellitus |
|  | E13 | Other specified diabetes mellitus |
|  | E14 | Unspecified diabetes mellitus |
| Malnutrition | E40.x to E46.x |  |
| Obesity | E66.x |  |
| Metabolic disorders | E70.x to E90.x |  |
| Cardiovascular diseases | I05.x to I09.x | Chronic rheumatic heart disease |
|  | I10.x to I15.x | Arterial hypertension |
|  | I20.x to I25.x | Ischemic heart diseases |
|  | I27.x | Other pulmonary heart diseases |
|  | I31.x, to I32.x | Other diseases of pericardium; Pericarditis in diseases classified elsewhere |
|  | I33.x | Acute and subacute endocarditis |
|  | I40.x to I41.x | Acute myocarditis; Myocarditis in diseases classified elsewhere |
|  | I42.x to I43.x | Cardiomyopathy; Cardiomyopathy in diseases classified elsewhere |
|  | I44.x to I49.x | Atrioventricular and left bundle-branch block; Other conduction disorders; Cardiac arrest; Paroxysmal tachycardia; Atrial fibrillation and flutter; Other cardiac arrhythmias |
|  | I50.x | Heart failure |
|  | I63.x to I67.x | Cerebral infarction; Occlusion and stenosis of precerebral arteries, not resulting in cerebral infarction; Occlusion and stenosis of cerebral arteries, not resulting in cerebral infarction; Other cerebrovascular diseases |
|  | I7.x | Diseases of arteries, arterioles and capillaries |
| Intracranial haemorrhage | I60.x | Subarachnoid haemorrhage |
|  | I61.x | Intracerebral haemorrhage |
|  | I62.x | Other nontraumatic intracranial haemorrhage |
| Venous thrombosis of the deep veins of the upper extremities | I80.81 | Venous thrombosis or embolism of the subclavian or axillary veins |
|  | I82.81 | Venous thrombosis or embolism of the jugular vein |
| Venous thrombosis not including deep vein thrombosis | I82.0, I82.1, I82.8, I82.9 |  |
| Respiratory diseases | J.x | Diseases of the respiratory system |
| Respiratory failure | J96.x |  |
| Pneumonia | J12.x to J15.x | Viral pneumonia; pneumonia due to streptococcus pneumonia; pneumonia due to haemophilus influenzae; other bacterial pneumonia; pneumonia with other organisms |
|  | J18.x | Unspecified bronchopneumonia |
|  | J20.x | Acute bronchitis |
|  | J21.x | Acute bronchiolitis |
|  | J45.x | Asthma |
| Peritonitis | K65.x |  |
| Osteomyelitis | M86.x |  |
| Renal failure | N17.x to N19.x |  |
| Glomerular diseases | N00.x to N08.x |  |
|  | N04.x | Nephrotic syndrome |
| Congenital heart diseases | Q20.x to Q24.x |  |
| Injury and trauma | S.x, | Injuries |
|  | T.x | Injuries involving multiple body areas, burns and corrosions, poisoning, and sequelae of injuries and poisoning |
|  | V.x | Transport accidents |
|  | W.x | Other external causes of accidental injury |
|  | X.x | Intentional self-harm and assault |
|  | Y.x | Event of undetermined intent; legal intervention and operations of war; complications of medical and surgical care |
| Corona virus disease 2019 | U07.1 | COVID-19, virus identified |
|  | U07.2 | COVID-19, virus not identified |
|  | U10.9 | Multisystem inflammatory syndrome associated with COVID-19, unspecified |

# Table S3. Definition of high-risk features based on ICD-10-GM codes and the Swiss classification of surgical procedures (CHOP).

| **Procedure** | **Year** | **CHOP Code** | **Definition** |
| --- | --- | --- | --- |
| Resuscitation | 2004-2023 | 99.60 | Cardiopulmonary resuscitation |
|  | 2004-2023 | 99.62 | Electric shock to the heart. |
|  | 2004-2023 | 99.63 | External cardiac massage |
|  | 2004-2015 | 93.93 | Non-mechanical methods of resuscitation |
| Drug use | 2004-2023 | 00.17 | Use of vasopressor substances |
|  | 2004-2023 | 99.10 | Use of thrombolytic substances |
| Extracorporeal membrane oxygenation | 2016-2023 | 37.69.8x | Duration of treatment with a cardiovascular and lung support system, with pump, with oxygenator (including CO2 removal), extracorporeal, veno-venous |
|  | 2016-2023 | 37.69.Ax | Duration of treatment with a cardiovascular and lung support system, with pump, with oxygenator (including CO2 removal), extracorporeal, veno-arterial or veno-venoarterial |
|  | 2016-2023 | 37.6A.61 | Implantation of a cardiovascular and lung support system, with pump, with oxygenator (including CO2 removal), extracorporeal, veno-venous, open surgical extra-thoracic |
|  | 2016-2023 | 37.6A.62 | Implantation of a cardiovascular and lung support system, with pump, with oxygenator (including CO2 removal), extracorporeal, veno-venous, percutaneous |
|  | 2016-2023 | 37.6A.71 | Implantation of a cardiovascular and lung support system, with pump, with oxygenator (including CO2 removal), extracorporeal, veno-arterial or veno-venoarterial, open surgical thoracic |
|  | 2016-2023 | 37.6A.72 | Implantation of a cardiovascular and lung support system, with pump, with oxygenator (including CO2 removal), extracorporeal, veno-arterial or veno-venoarterial, open surgical extrathoracic |
|  | 2016-2023 | 37.6A.73 | Implantation eines herzkreislauf- und lungenunterstützenden Systems, mit Pumpe, mit Oxygenator (inkl. CO2-removal), extrakorporal, veno-arteriell oder venovenoarteriell, perkutan |
|  | 2016-2023 | 37.6B.61 | Implantation of a cardiovascular and lung support system, with pump, with oxygenator (including CO2 removal), extracorporeal, veno-arterial or veno-venoarterial, percutaneous |
|  | 2016-2023 | 37.6B.62 | Removal of a cardiovascular and lung support system, with pump, with oxygenator (including CO2 removal), extracorporeal, veno-venous, percutaneous. |
|  | 2016-2023 | 37.6B.71 | Removal of a cardiovascular and lung support system, with pump, with oxygenator (including CO2 removal), extracorporeal, veno-arterial or veno-venoarterial, open surgical thoracic |
|  | 2016-2023 | 37.6B.72 | Removal of a cardiovascular and lung support system, with pump, with oxygenator (including CO2 removal), extracorporeal, veno-arterial or veno-venoarterial, open surgical extrathoracic |
|  | 2016-2023 | 37.6B.73 | Removal of a cardiovascular and lung support system, with pump, with oxygenator (including CO2 removal), extracorporeal, veno-arterial or veno-venoarterial, percutaneously. |
|  | 2016-2023 | 37.6C.61 | Revision without replacement of a cardiovascular and lung support system, with pump, with oxygenator (including CO2 removal), extracorporeal, veno-venous, open surgical extrathoracic |
|  | 2016-2023 | 37.6C.62 | Revision without replacement of a cardiovascular and lung support system, with pump, with oxygenator (including CO2 removal), extracorporeal, veno-venous, percutaneous. |
|  | 2016-2023 | 37.6C.71 | Revision without replacement of a cardiovascular and lung support system, with pump, with oxygenator (including CO2 removal), extracorporeal, veno-arterial or veno-venoarterial. |
|  | 2016-2023 | 37.6D.31 | Ersatz einer patientenfernen Teilkomponente eines herzkreislauf- und lungenunterstützenden Systems, mit Pumpe, mit Oxygenator (inkl. CO2- removal), extrakorporal, veno-venös |
|  | 2016-2023 | 37.6D.41 | Replacement of a non-patient-contacting component of a cardiovascular and lung support system, with pump, with oxygenator (including CO2 removal), extracorporeal, veno-arterial or veno-venoarterial. |
|  | 2004-2015 | 39.65 | ECMO |
| Invasive ventilation | 2016-2023 | 93.9E.xx | Non-invasive ventilation outside the intensive care unit, duration of treatment in days. |
|  | 2016-2023 | 93.9F.xx | Mechanical ventilation and respiratory support |
|  | 2016-2023 | 93.9G.5x | Invasive ventilation, initial setting, duration |
|  | 2016-2023 | 93.9G.6x | Invasive ventilation, control and optimization of an existing setting, duration |
|  | 2016-2023 | 93.9G.7x | Invasive ventilation treatment duration |
|  | 2016-2023 | 93.9B | Measures to secure the upper airway for ventilation |
|  | 2012-2015 | 96.Ax | Mechanical ventilation via mask and endotracheal tube |
|  | 2004-2015 | 93.90.x | Non-invasive mechanical ventilation |
|  | 2004-2015 | 93.91 | Intermittent positive pressure ventilation (IPPB) |
|  | 2004-2015 | 93.95.x | Hyperbaric oxygenation |
|  | 2004-2015 | 96.7 | Other continuous mechanical ventilation |
| Surgical embolectomy | 2004-2023 | 38.05 | Surgical embolectomy of thoracal vessels (excl. aorta) |
|  |  |  |  |
|  |  | **ICD-10-GM Code** | **Definition** |
|  |  |  |  |
|  |  | R57 | Shock |
|  |  | I46 | Cardiac arrest |

# Table S4. Definition of therapeutic procedures based on Swiss classification of surgical procedures (CHOP) codes.

| **Procedure** | **Year** | **CHOP Code** | **Definition** |
| --- | --- | --- | --- |
| Therapeutic procedures | | | |
| Intervention at pulmonary vessels | 2011-2023 | 00.4B.13 |  |
| Intervention at abdominal veins | 2011-2023 | 00.4B.26 |  |
| Catheter-directed treatment of the pulmonary arteries | 2018-2023 | 00.4F | Usage of a thrombus retrieval system |
|  | 2008-2023 | 39.75 | Percutaneous vascular intervention |
| Inferior vena cava filter placement | 2004-2011 | 38.7 | Interruption of the vena cava |
|  | 2012-2023 | 38.7X.21 | Implantation of a vena cava filter |
| Right-heart catheter | 2004-2023 | 37.21 | Right heart catheter |
|  | 2004-2023 | 37.23 | Combined right and left heart catheter |
| Extracorporeal membrane oxygenation | 2016-2023 | 37.69.8x | Duration of treatment with a cardiovascular and lung support system, with pump, with oxygenator (including CO2 removal), extracorporeal, veno-venous |
|  | 2016-2023 | 37.69.Ax | Duration of treatment with a cardiovascular and lung support system, with pump, with oxygenator (including CO2 removal), extracorporeal, veno-arterial or veno-venoarterial |
|  | 2016-2023 | 37.6A.61 | Implantation of a cardiovascular and lung support system, with pump, with oxygenator (including CO2 removal), extracorporeal, veno-venous, open surgical extra-thoracic |
|  | 2016-2023 | 37.6A.62 | Implantation of a cardiovascular and lung support system, with pump, with oxygenator (including CO2 removal), extracorporeal, veno-venous, percutaneous |
|  | 2016-2023 | 37.6A.71 | Implantation of a cardiovascular and lung support system, with pump, with oxygenator (including CO2 removal), extracorporeal, veno-arterial or veno-venoarterial, open surgical thoracic |
|  | 2016-2023 | 37.6A.72 | Implantation of a cardiovascular and lung support system, with pump, with oxygenator (including CO2 removal), extracorporeal, veno-arterial or veno-venoarterial, open surgical extrathoracic |
|  | 2016-2023 | 37.6A.73 | Implantation of a cardiovascular and lung support system with a pump and oxygenator (including CO₂ removal), extracorporeal, veno-arterial or veno-veno-arterial, percutaneous. |
|  | 2016-2023 | 37.6B.61 | Implantation of a cardiovascular and lung support system, with pump, with oxygenator (including CO2 removal), extracorporeal, veno-arterial or veno-venoarterial, percutaneous |
|  | 2016-2023 | 37.6B.62 | Removal of a cardiovascular and lung support system, with pump, with oxygenator (including CO2 removal), extracorporeal, veno-venous, percutaneous. |
|  | 2016-2023 | 37.6B.71 | Removal of a cardiovascular and lung support system, with pump, with oxygenator (including CO2 removal), extracorporeal, veno-arterial or veno-venoarterial, open surgical thoracic |
|  | 2016-2023 | 37.6B.72 | Removal of a cardiovascular and lung support system, with pump, with oxygenator (including CO2 removal), extracorporeal, veno-arterial or veno-venoarterial, open surgical extrathoracic |
|  | 2016-2023 | 37.6B.73 | Removal of a cardiovascular and lung support system, with pump, with oxygenator (including CO2 removal), extracorporeal, veno-arterial or veno-venoarterial, percutaneously. |
|  | 2016-2023 | 37.6C.61 | Revision without replacement of a cardiovascular and lung support system, with pump, with oxygenator (including CO2 removal), extracorporeal, veno-venous, open surgical extrathoracic |
|  | 2016-2023 | 37.6C.62 | Revision without replacement of a cardiovascular and lung support system, with pump, with oxygenator (including CO2 removal), extracorporeal, veno-venous, percutaneous. |
|  | 2016-2023 | 37.6C.71 | Revision without replacement of a cardiovascular and lung support system, with pump, with oxygenator (including CO2 removal), extracorporeal, veno-arterial or veno-venoarterial. |
|  | 2016-2023 | 37.6D.31 | Replacement of a remote component of a cardiovascular and lung support system with a pump and oxygenator (including CO₂ removal), extracorporeal, veno-venous. |
|  | 2016-2023 | 37.6D.41 | Replacement of a non-patient-contacting component of a cardiovascular and lung support system, with pump, with oxygenator (including CO2 removal), extracorporeal, veno-arterial or veno-venoarterial. |
|  | 2004-2015 | 39.65 | ECMO |
| Surgical embolectomy | 2004-2023 | 38.05 | Surgical embolectomy of thoracal vessels (excl. aorta) |
| Central venous catheter | 2004-2023 | 38.93 |  |
| Usage of a heart-lung machine (excl. ECMO) | 2004-2023 | 39.61 |  |
| Haemodialysis | 2004-2023 | 39.95 |  |
| Invasive ventilation | 2016-2023 | 93.9E.xx | Non-invasive ventilation outside the intensive care unit, duration of treatment in days. |
|  | 2016-2023 | 93.9F.xx | Mechanical ventilation and respiratory support |
|  | 2016-2023 | 93.9G.5x | Invasive ventilation, initial setting, duration |
|  | 2016-2023 | 93.9G.6x | Invasive ventilation, control and optimization of an existing setting, duration |
|  | 2016-2023 | 93.9G.7x | Invasive ventilation treatment duration |
|  | 2016-2023 | 93.9B | Measures to secure the upper airway for ventilation |
|  | 2012-2015 | 96.Ax | Mechanical ventilation via mask and endotracheal tube |
|  | 2004-2015 | 93.90.x | Non-invasive mechanical ventilation |
|  | 2004-2015 | 93.91 | Intermittent positive pressure ventilation (IPPB) |
|  | 2004-2015 | 93.95.x | Hyperbaric oxygenation |
|  | 2004-2015 | 96.7 | Other continuous mechanical ventilation |
| Transfusions of blood or blood components | 2004-2023 | 99.0 | Any transfusion |
|  | 2004-2023 | 99.00 | Transfusion of erythrozytes |
|  | 2004-2023 | 99.05 | Transfusion of thrombozytes |
|  | 2004-2023 | 99.07 | Transfusion of plasma |
|  | 2017-2023 | 99.0A | Transfusion of coagulatin factors |
| Use of thrombolytic substances | 2004-2023 | 99.10 |  |
| Enteral/parenteral nutrition | 2004-2023 | 96.6 | Enteral infusion of concentrated nutrient solution |
|  | 2004-2023 | 99.15 | Parenteral infusion of concentrated nutrient solution |

# Table S5. Overview of incident venous thromboembolism cases, and venous thromboembolism related deaths per age group, stratified by sex.

| **Characteristic** | **Venous thromboembolism** | | | **Pulmonary embolism (with or without deep vein thrombosis)** | | | **Deep vein thrombosis (excluding cases with concomitant pulmonary embolism diagnosis)** | | |
| --- | --- | --- | --- | --- | --- | --- | --- | --- | --- |
|  | **Overall,** N = 1,961 | **Female,** N = 1,037 | **Male,** N = 924 | **Overall,** N = 815 | **Female,** N = 497 | **Male,** N = 318 | **Overall,** N = 1,146 | **Female,** N = 540 | **Male,** N = 606 |
| Hospitalizations for or with the disease, n (%) |  |  |  |  |  |  |  |  |  |
| 0 years old | 351 (21.2%) | 136 (15.2%) | 215 (28.3%) | 28 (3.9%) | 15 (3.4%) | 13 (4.9%) | 323 (34.3%) | 121 (27.0%) | 202 (40.9%) |
| 1 to 4 years old | 135 (8.2%) | 61 (6.8%) | 74 (9.7%) | 18 (2.5%) | 9 (2.0%) | 9 (3.4%) | 117 (12.4%) | 52 (11.6%) | 65 (13.2%) |
| 5 to 9 years old | 99 (6.0%) | 43 (4.8%) | 56 (7.4%) | 24 (3.4%) | 9 (2.0%) | 15 (5.6%) | 75 (8.0%) | 34 (7.6%) | 41 (8.3%) |
| 10 to 14 years old | 161 (9.7%) | 67 (7.5%) | 94 (12.4%) | 60 (8.4%) | 26 (5.8%) | 34 (12.8%) | 101 (10.7%) | 41 (9.2%) | 60 (12.1%) |
| 15 to 19 years old | 907 (54.9%) | 586 (65.6%) | 321 (42.2%) | 581 (81.7%) | 386 (86.7%) | 195 (73.3%) | 326 (34.6%) | 200 (44.6%) | 126 (25.5%) |
| Median age of patients at hospital admission, years (Q1-Q3) | 15 (2, 18) | 17 (8, 18) | 12 (0, 17) | 17 (16, 18) | 17 (16, 18) | 17 (14, 18) | 7 (0, 16) | 13 (0, 17) | 3 (0, 15) |
| First decile (D1), years | 0 | 0 | 0 | 10 | 13 | 7 | 0 | 0 | 0 |
| Death with any mention of the disease, n (%) | 78 (4.7%) | 40 (4.5%) | 38 (5.0%) | 35 (4.9%) | 21 (4.7%) | 14 (5.3%) | 43 (4.6%) | 19 (4.2%) | 24 (4.9%) |
| 0 years old | 43 (2.6%) | 19 (2.1%) | 24 (3.2%) | 13 (1.8%) | 8 (1.8%) | 5 (1.9%) | 30 (3.2%) | 11 (2.5%) | 19 (3.8%) |
| 1 to 4 years old | 6 (0.4%) | 2 (0.2%) | 4 (0.5%) | 1 (0.1%) | 0 (0.0%) | 1 (0.4%) | 5 (0.5%) | 2 (0.4%) | 3 (0.6%) |
| 5 to 9 years old | 3 (0.2%) | 3 (0.3%) | 0 (0.0%) | 1 (0.1%) | 1 (0.2%) | 0 (0.0%) | 2 (0.2%) | 2 (0.4%) | 0 (0.0%) |
| 10 to 14 years old | 6 (0.4%) | 3 (0.3%) | 3 (0.4%) | 3 (0.4%) | 2 (0.4%) | 1 (0.4%) | 3 (0.3%) | 1 (0.2%) | 2 (0.4%) |
| 15 to 19 years old | 20 (1.2%) | 13 (1.5%) | 7 (0.9%) | 17 (2.4%) | 10 (2.2%) | 7 (2.6%) | 3 (0.3%) | 3 (0.7%) | 0 (0.0%) |
| Median age at disease related death, years (Q1-Q3) | 0 (0, 16) | 3.5 (0, 17) | 0 (0, 12) | 14 (0, 18) | 13 (0, 18) | 15 (0, 17) | 0 (0, 2) | 0 (0, 9) | 0 (0, 0) |

# Table S6. Overview of venous thromboembolism (VTE) cases, and VTE-related deaths per age group, stratified by sex and time period.

| **Characteristic** | **Overall,** N = 1,961 | **2004-2013** | | | **2014-2023** | | |
| --- | --- | --- | --- | --- | --- | --- | --- |
|  |  | **Overall**, N = 908 | **Female**, N = 559 | **Male**, N = 349 | **Overall**, N = 1,053 | **Female**, N = 478 | **Male**, N = 575 |
| Hospitalizations for or with the disease, n (%) |  |  |  |  |  |  |  |
| 0 years old | 393 (20.0%) | 122 (13.4%) | 54 (9.7%) | 68 (19.5%) | 271 (25.7%) | 94 (19.7%) | 177 (30.8%) |
| 1 to 4 years old | 166 (8.5%) | 55 (6.1%) | 24 (4.3%) | 31 (8.9%) | 111 (10.5%) | 46 (9.6%) | 65 (11.3%) |
| 5 to 9 years old | 120 (6.1%) | 48 (5.3%) | 20 (3.6%) | 28 (8.0%) | 72 (6.8%) | 38 (7.9%) | 34 (5.9%) |
| 10 to 14 years old | 200 (10.2%) | 91 (10.0%) | 49 (8.8%) | 42 (12.0%) | 109 (10.4%) | 39 (8.2%) | 70 (12.2%) |
| 15 to 19 years old | 1,082 (55.2%) | 592 (65.2%) | 412 (73.7%) | 180 (51.6%) | 490 (46.5%) | 261 (54.6%) | 229 (39.8%) |
| Median age of patients at hospital admission, years (Q1-Q3) | 15 (2, 18) | 16 (10, 18) | 17 (14, 18) | 15 (3, 18) | 14 (0, 18) | 15 (2, 18) | 11 (0, 17) |
| First decile | 0 | 0 | 1 | 0 | 0 | 0 | 0 |
| Death with any mention of the disease, n (%) | 79 (4.0%) | 26 (2.9%) | 17 (3.0%) | 9 (2.6%) | 53 (5.0%) | 24 (5.0%) | 29 (5.0%) |
| 0 years old | 43 (2.2%) | 13 (1.4%) | 8 (1.4%) | 5 (1.4%) | 30 (2.8%) | 11 (2.3%) | 19 (3.3%) |
| 1 to 4 years old | 6 (0.3%) | 1 (0.1%) | 0 | 1 (0.3%) | 5 (0.5%) | 2 (0.4%) | 3 (0.5%) |
| 5 to 9 years old | 3 (0.2%) | 2 (0.2%) | 2 (0.4%) | 0 | 1 (0.1%) | 1 (0.2%) | 0 |
| 10 to 14 years old | 7 (0.4%) | 2 (0.2%) | 2 (0.4%) | 0 | 5 (0.5%) | 2 (0.4%) | 3 (0.5%) |
| 15 to 19 years old | 20 (1.0%) | 8 (0.9%) | 5 (0.9%) | 3 (0.9%) | 12 (1.1%) | 8 (1.7%) | 4 (0.7%) |
| Median age at disease related death, years (Q1-Q3) | 0.0 (0.0, 16.0) | 1.0 (0.0, 17.0) | 7.0 (0.0, 17.0) | 0.0 (0.0, 17.0) | 0.0 (0.0, 14.0) | 3.5 (0.0, 17.0) | 0.0 (0.0, 1.0) |

# Table S7. Overview of the pulmonary embolism (PE) and deep vein thrombosis (DVT) of the lower extremity cases, and PE-related and DVT-related deaths per age group, stratified by sex and time period.

| **Characteristic** | **Both diseases,** N = 1,961 | **Pulmonary embolism (with or without concomitant deep vein thrombosis)** | | | | | | | **Deep vein thrombosis (excluding cases with concomitant pulmonary embolism diagnosis)** | | | | | | |
| --- | --- | --- | --- | --- | --- | --- | --- | --- | --- | --- | --- | --- | --- | --- | --- |
|  |  | **Overall**, N = 815 | **2004-2013** | | | **2014-2023** | | | **Overall**, N = 1,146 | **2004-2013** | | | **2014-2023** | | |
|  |  |  | **Overall**, N = 410 | **Female**, N = 293 | **Male**, N = 117 | **Overall**, N = 405 | **Female**, N = 204 | **Male**, N = 201 |  | **Overall**, N = 498 | **Female**, N = 266 | **Male**, N = 232 | **Overall**, N = 648 | **Female**, N = 274 | **Male**, N = 374 |
| Hospitalizations for or with the disease, n (%) |  |  |  |  |  |  |  |  |  |  |  |  |  |  |  |
| 0 years old | 393 (20.0%) | 29 (3.6%) | 8 (2.0%) | 5 (1.7%) | 3 (2.6%) | 21 (5.2%) | 10 (4.9%) | 11 (5.5%) | 364 (31.8%) | 114 (22.9%) | 49 (18.4%) | 65 (28.0%) | 250 (38.6%) | 84 (30.7%) | 166 (44.4%) |
| 1 to 4 years old | 166 (8.5%) | 25 (3.1%) | 7 (1.7%) | 4 (1.4%) | 3 (2.6%) | 18 (4.4%) | 6 (2.9%) | 12 (6.0%) | 141 (12.3%) | 48 (9.6%) | 20 (7.5%) | 28 (12.1%) | 93 (14.4%) | 40 (14.6%) | 53 (14.2%) |
| 5 to 9 years old | 120 (6.1%) | 27 (3.3%) | 14 (3.4%) | 4 (1.4%) | 10 (8.5%) | 13 (3.2%) | 6 (2.9%) | 7 (3.5%) | 93 (8.1%) | 34 (6.8%) | 16 (6.0%) | 18 (7.8%) | 59 (9.1%) | 32 (11.7%) | 27 (7.2%) |
| 10 to 14 years old | 200 (10.2%) | 68 (8.3%) | 23 (5.6%) | 15 (5.1%) | 8 (6.8%) | 45 (11.1%) | 17 (8.3%) | 28 (13.9%) | 132 (11.5%) | 68 (13.7%) | 34 (12.8%) | 34 (14.7%) | 64 (9.9%) | 22 (8.0%) | 42 (11.2%) |
| 15 to 19 years old | 1,082 (55.2%) | 666 (81.7%) | 358 (87.3%) | 265 (90.4%) | 93 (79.5%) | 308 (76.0%) | 165 (80.9%) | 143 (71.1%) | 416 (36.3%) | 234 (47.0%) | 147 (55.3%) | 87 (37.5%) | 182 (28.1%) | 96 (35.0%) | 86 (23.0%) |
| Median age of patients at hospital admission, years (Q1-Q3) | 15 (2, 18) | 17 (16, 18) | 17 (16, 18) | 17 (16, 18) | 17 (15, 18) | 17 (15, 18) | 17 (15, 18) | 17 (14, 18) | 8 (0, 16) | 14 (1, 17) | 15 (4, 18) | 11 (0, 17) | 3 (0, 15) | 7 (0, 16) | 2 (0, 14) |
| First decile, years | 0 | 10 | 13 | 15 | 8 | 6 | 9 | 2 | 0 | 0 | 0 | 0 | 0 | 0 | 0 |
| Death with any mention of the disease, n (%) | 79 (4.0%) | 35 (4.3%) | 15 (3.7%) | 10 (3.4%) | 5 (4.3%) | 20 (4.9%) | 11 (5.4%) | 9 (4.5%) | 44 (3.8%) | 11 (2.2%) | 7 (2.6%) | 4 (1.7%) | 33 (5.1%) | 13 (4.7%) | 20 (5.3%) |
| 0 years old | 43 (2.2%) | 13 (1.6%) | 5 (1.2%) | 3 (1.0%) | 2 (1.7%) | 8 (2.0%) | 5 (2.5%) | 3 (1.5%) | 30 (2.6%) | 8 (1.6%) | 5 (1.9%) | 3 (1.3%) | 22 (3.4%) | 6 (2.2%) | 16 (4.3%) |
| 1 to 4 years old | 6 (0.3%) | 1 (0.1%) | 0 | 0 | 0 | 1 (0.2%) | 0 | 1 (0.5%) | 5 (0.4%) | 1 (0.2%) | 0 | 1 (0.4%) | 4 (0.6%) | 2 (0.7%) | 2 (0.5%) |
| 5 to 9 years old | 3 (0.2%) | 1 (0.1%) | 1 (0.2%) | 1 (0.3%) | 0 | 0 | 0 | 0 | 2 (0.2%) | 1 (0.2%) | 1 (0.4%) | 0 | 1 (0.2%) | 1 (0.4%) | 0 |
| 10 to 14 years old | 7 (0.4%) | 3 (0.4%) | 1 (0.2%) | 1 (0.3%) | 0 | 2 (0.5%) | 1 (0.5%) | 1 (0.5%) | 4 (0.3%) | 1 (0.2%) | 1 (0.4%) | 0 | 3 (0.5%) | 1 (0.4%) | 2 (0.5%) |
| 15 to 19 years old | 20 (1.0%) | 17 (2.1%) | 8 (2.0%) | 5 (1.7%) | 3 (2.6%) | 9 (2.2%) | 5 (2.5%) | 4 (2.0%) | 3 (0.3%) | 0 | 0 | 0 | 3 (0.5%) | 3 (1.1%) | 0 |
| Median age at disease related death, years (Q1-Q3) | 0.0 (0.0, 16.0) | 14 (0, 18) | 17 (0, 18) | 15 (0, 18) | 17 (0, 17) | 12 (0, 17.5) | 10 (0, 18) | 14 (0, 16) | 0.0 (0.0, 2.5) | 0.0 (0.0, 2.0) | 0.0 (0.0, 7.0) | 0.0 (0.0, 1.0) | 0.0 (0.0, 3.0) | 3.0 (0.0, 11.0) | 0.0 (0.0, 0.0) |

# Table S8. Overview of high-risk features mentioned in venous thromboembolism cases, intensive care unit (ICU) stay, and length of hospitalization, stratified by age and time period.

| **Characteristic** | **Overall,** N = 1,961 | **2004-2013** | | | **2014-2023** | | |
| --- | --- | --- | --- | --- | --- | --- | --- |
|  |  | **Overall**, N = 908 | **0 to 9 years old**, N = 225 | **10 to 19 years old**, N = 683 | **Overall**, N = 1,053 | **0 to 9 years old**, N = 454 | **10 to 19 years old**, N = 599 |
| High-risk features, n (%) | 592 (30.2%) | 192 (21.1%) | 115 (51.1%) | 77 (11.3%) | 400 (38.0%) | 258 (56.8%) | 142 (23.7%) |
| Invasive ventilation | 367 (18.7%) | 125 (13.8%) | 97 (43.1%) | 28 (4.1%) | 242 (23.0%) | 194 (42.7%) | 48 (8.0%) |
| Use of vasopressors | 202 (10.3%) | 34 (3.7%) | 26 (11.6%) | 8 (1.2%) | 168 (16.0%) | 129 (28.4%) | 39 (6.5%) |
| Use of systemic thrombolysis | 150 (7.6%) | 62 (6.8%) | 19 (8.4%) | 43 (6.3%) | 88 (8.4%) | 30 (6.6%) | 58 (9.7%) |
| Shock | 141 (7.2%) | 25 (2.8%) | 15 (6.7%) | 10 (1.5%) | 116 (11.0%) | 68 (15.0%) | 48 (8.0%) |
| Cardiac arrest | 93 (4.7%) | 30 (3.3%) | 18 (8.0%) | 12 (1.8%) | 63 (6.0%) | 48 (10.6%) | 15 (2.5%) |
| Extracorporeal membrane oxygenation | 68 (3.5%) | 17 (1.9%) | 11 (4.9%) | 6 (0.9%) | 51 (4.8%) | 31 (6.8%) | 20 (3.3%) |
| Surgical thrombectomy | 13 (0.7%) | 6 (0.7%) | 2 (0.9%) | 4 (0.6%) | 7 (0.7%) | 5 (1.1%) | 2 (0.3%) |
| Hospital type, n (%) |  |  |  |  |  |  |  |
| University hospital | 647 (33.0%) | 244 (26.9%) | 82 (36.4%) | 162 (23.7%) | 403 (38.3%) | 186 (41.0%) | 217 (36.2%) |
| Major hospital | 565 (28.8%) | 272 (30.0%) | 30 (13.3%) | 242 (35.4%) | 293 (27.8%) | 55 (12.1%) | 238 (39.7%) |
| Regional hospital | 276 (14.1%) | 221 (24.3%) | 1 (0.4%) | 220 (32.2%) | 55 (5.2%) | 2 (0.4%) | 53 (8.8%) |
| Specialized pediatric hospitals | 435 (22.2%) | 161 (17.7%) | 112 (49.8%) | 49 (7.2%) | 274 (26.0%) | 210 (46.3%) | 64 (10.7%) |
| Others | 38 (1.9%) | 10 (1.1%) | 0 (0.0%) | 10 (1.5%) | 28 (2.7%) | 1 (0.2%) | 27 (4.5%) |
| Median length of hospitalization, days (IQR) | 8 (3, 24) | 7 (3, 15) | 21 (9, 55) | 5 (3, 9) | 11 (3, 34) | 25 (9, 58) | 6 (2, 17) |
| Stay in ICU, n (%) | 771 (39.3%) | 262 (28.9%) | 153 (68.0%) | 109 (16.0%) | 509 (48.3%) | 328 (72.2%) | 181 (30.2%) |
| Median length of stay on an ICU (if patient was admitted to ICU), hours (IQR) | 210 (59, 696) | 178 (40, 581) | 326 (144, 1,001) | 49 (23, 112) | 218 (67, 729) | 383 (140, 917) | 76 (27, 272) |

# Table S9. Overview of high-risk features mentioned in pulmonary embolism (PE) and deep vein thrombosis (DVT) cases, intensive care unit (ICU) stay, and length of hospitalization, stratified by age and time period.

| **Characteristic** | **Both diseases,** N = 1,961 | **Pulmonary embolism (with or without concomitant deep vein thrombosis)** | | | | | | | **Deep vein thrombosis (excluding cases with concomitant pulmonary embolism diagnosis)** | | | | | | |
| --- | --- | --- | --- | --- | --- | --- | --- | --- | --- | --- | --- | --- | --- | --- | --- |
|  |  | **Overall**, N = 815 | **2004-2013** | | | **2014-2023** | | | **Overall**, N = 1,146 | **2004-2013** | | | **2014-2023** | | |
|  |  |  | **Overall**, N = 410 | **0 to 9 years old**, N = 29 | **10 to 19 years old**, N = 381 | **Overall**, N = 405 | **0 to 9 years old**, N = 52 | **10 to 19 years old**, N = 353 |  | **Overall**, N = 498 | **0 to 9 years old**, N = 196 | **10 to 19 years old**, N = 302 | **Overall**, N = 648 | **0 to 9 years old**, N = 402 | **10 to 19 years old**, N = 246 |
| High-risk features, n (%) | 592 (30.2%) | 165 (20.2%) | 52 (12.7%) | 11 (42.3%) | 41 (10.8%) | 113 (27.9%) | 32 (61.5%) | 81 (22.9%) | 427 (37.3%) | 140 (28.1%) | 104 (53.1%) | 36 (11.9%) | 287 (44.3%) | 226 (56.2%) | 61 (24.8%) |
| Invasive ventilation | 367 (18.7%) | 78 (9.6%) | 25 (6.1%) | 8 (27.6%) | 17 (4.5%) | 53 (13.1%) | 21 (40.4%) | 32 (9.1%) | 289 (25.2%) | 100 (20.1%) | 89 (45.4%) | 11 (3.6%) | 189 (29.2%) | 173 (43.0%) | 16 (6.5%) |
| Use of vasopressors | 202 (10.3%) | 53 (6.5%) | 10 (2.4%) | 4 (13.8%) | 6 (1.6%) | 43 (10.6%) | 15 (28.8%) | 28 (7.9%) | 149 (13.0%) | 24 (4.8%) | 22 (11.2%) | 2 (0.7%) | 125 (19.3%) | 114 (28.4%) | 11 (4.5%) |
| Use of systemic thrombolysis | 150 (7.6%) | 57 (7.0%) | 26 (6.3%) | 5 (17.2%) | 21 (5.5%) | 31 (7.7%) | 6 (11.5%) | 25 (7.1%) | 93 (8.1%) | 36 (7.2%) | 14 (7.1%) | 22 (7.3%) | 57 (8.8%) | 24 (6.0%) | 33 (13.4%) |
| Shock | 141 (7.2%) | 61 (7.5%) | 7 (1.7%) | 0 (0.0%) | 7 (1.8%) | 54 (13.3%) | 16 (30.8%) | 38 (10.8%) | 80 (7.0%) | 18 (3.6%) | 15 (7.7%) | 3 (1.0%) | 62 (9.6%) | 52 (12.9%) | 10 (4.1%) |
| Cardiac arrest | 93 (4.7%) | 33 (4.0%) | 12 (2.9%) | 3 (11.5%) | 9 (2.4%) | 21 (5.2%) | 12 (23.1%) | 9 (2.5%) | 60 (5.2%) | 18 (3.6%) | 15 (7.7%) | 3 (1.0%) | 42 (6.5%) | 36 (9.0%) | 6 (2.4%) |
| Extracorporeal membrane oxygenation | 68 (3.5%) | 23 (2.8%) | 6 (1.5%) | 2 (6.9%) | 4 (1.0%) | 17 (4.2%) | 6 (11.5%) | 11 (3.1%) | 45 (3.9%) | 11 (2.2%) | 9 (4.6%) | 2 (0.7%) | 34 (5.2%) | 25 (6.2%) | 9 (3.7%) |
| Surgical thrombectomy | 13 (0.7%) | 6 (0.7%) | 3 (0.7%) | 0 (0.0%) | 3 (0.8%) | 3 (0.7%) | 2 (3.8%) | 1 (0.3%) | 7 (0.6%) | 3 (0.6%) | 2 (1.0%) | 1 (0.3%) | 4 (0.6%) | 3 (0.7%) | 1 (0.4%) |
| Hospital type, n (%) |  |  |  |  |  |  |  |  |  |  |  |  |  |  |  |
| University hospital | 647 (33.0%) | 245 (30.1%) | 103 (25.1%) | 14 (48.3%) | 89 (23.4%) | 142 (35.1%) | 31 (59.6%) | 111 (31.4%) | 402 (35.1%) | 141 (28.3%) | 68 (34.7%) | 73 (24.2%) | 261 (40.3%) | 155 (38.6%) | 106 (43.1%) |
| Major hospital | 565 (28.8%) | 339 (41.6%) | 163 (39.8%) | 4 (13.8%) | 159 (41.7%) | 176 (43.5%) | 6 (11.5%) | 170 (48.2%) | 226 (19.7%) | 109 (21.9%) | 26 (13.3%) | 83 (27.5%) | 117 (18.1%) | 49 (12.2%) | 68 (27.6%) |
| Regional hospital | 276 (14.1%) | 155 (19.0%) | 115 (28.0%) | 0 (0.0%) | 115 (30.2%) | 40 (9.9%) | 0 (0.0%) | 40 (11.3%) | 121 (10.6%) | 106 (21.3%) | 1 (0.5%) | 105 (34.8%) | 15 (2.3%) | 2 (0.5%) | 13 (5.3%) |
| Specialized pediatric hospitals | 435 (22.2%) | 58 (7.1%) | 26 (6.3%) | 11 (37.9%) | 15 (3.9%) | 32 (7.9%) | 15 (28.8%) | 17 (4.8%) | 377 (32.9%) | 135 (27.1%) | 101 (51.5%) | 34 (11.3%) | 242 (37.3%) | 195 (48.5%) | 47 (19.1%) |
| Others | 38 (1.9%) | 18 (2.2%) | 3 (0.7%) | 0 (0.0%) | 3 (0.8%) | 15 (3.7%) | 0 (0.0%) | 15 (4.2%) | 20 (1.7%) | 7 (1.4%) | 0 (0.0%) | 7 (2.3%) | 13 (2.0%) | 1 (0.2%) | 12 (4.9%) |
| Median length of hospitalization, days (IQR) | 8 (3, 24) | 6 (3, 17) | 6 (3, 10) | 15 (9, 36) | 5 (3, 9) | 7 (3, 21) | 22 (7, 92) | 6 (3, 18) | 12 (4, 31) | 8 (3, 21) | 22 (9, 57) | 5 (2, 10) | 15 (4, 45) | 25 (9, 58) | 5 (2, 16) |
| Stay in ICU, n (%) | 771 (39.3%) | 267 (32.8%) | 95 (23.2%) | 19 (73.1%) | 76 (19.9%) | 172 (42.5%) | 40 (76.9%) | 132 (37.4%) | 504 (44.0%) | 167 (33.5%) | 134 (68.4%) | 33 (10.9%) | 337 (52.0%) | 288 (71.6%) | 49 (19.9%) |
| Median length of stay on an ICU (if patient was admitted to ICU), hours (IQR) | 210 (59, 696) | 75 (27, 279) | 62 (24, 192) | 308 (127, 2,374) | 41 (23, 107) | 93 (35, 322) | 434 (146, 1,870) | 63 (23, 191) | 316 (97, 842) | 300 (83, 785) | 339 (158, 1,001) | 76 (24, 183) | 338 (107, 853) | 382 (136, 894) | 128 (53, 632) |

ICU: intensive care unit

# Table S10. Overview of high-risk features mentioned in venous thromboembolism cases, intensive care unit (ICU) stay, and length of hospitalization, stratified by age and sex.

| **Characteristic** | **Overall,** N = 1,961 | **Female** | | | **Male** | | |
| --- | --- | --- | --- | --- | --- | --- | --- |
|  |  | **Overall**, N = 1,037 | **0 to 9 years old**, N = 276 | **10 to 19 years old**, N = 761 | **Overall**, N = 924 | **0 to 9 years old**, N = 403 | **10 to 19 years old**, N = 521 |
| High-risk features, n (%) | 592 (30.2%) | 281 (27.1%) | 156 (56.5%) | 125 (16.4%) | 311 (33.7%) | 217 (53.8%) | 94 (18%) |
| Invasive ventilation | 367 (18.7%) | 158 (15.2%) | 120 (43.5%) | 38 (5.0%) | 209 (22.6%) | 171 (42.4%) | 38 (7.3%) |
| Use of vasopressors | 202 (10.3%) | 85 (8.2%) | 61 (22.1%) | 24 (3.2%) | 117 (12.7%) | 94 (23.3%) | 23 (4.4%) |
| Use of systemic thrombolysis | 150 (7.6%) | 96 (9.3%) | 22 (8.0%) | 74 (9.7%) | 54 (5.8%) | 27 (6.7%) | 27 (5.2%) |
| Shock | 141 (7.2%) | 66 (6.4%) | 37 (13.4%) | 29 (3.8%) | 75 (8.1%) | 46 (11.4%) | 29 (5.6%) |
| Cardiac arrest | 93 (4.7%) | 48 (4.6%) | 31 (11.2%) | 17 (2.2%) | 45 (4.9%) | 35 (8.7%) | 10 (1.9%) |
| Extracorporeal membrane oxygenation | 68 (3.5%) | 33 (3.2%) | 16 (5.8%) | 17 (2.2%) | 35 (3.8%) | 26 (6.5%) | 9 (1.7%) |
| Surgical thrombectomy | 13 (0.7%) | 8 (0.8%) | 4 (1.4%) | 4 (0.5%) | 5 (0.5%) | 3 (0.7%) | 2 (0.4%) |
| Hospital type, n (%) |  |  |  |  |  |  |  |
| University hospital | 647 (33.0%) | 311 (30.0%) | 118 (42.8%) | 193 (25.4%) | 336 (36.4%) | 150 (37.2%) | 186 (35.7%) |
| Major hospital | 565 (28.8%) | 341 (32.9%) | 30 (10.9%) | 311 (40.9%) | 224 (24.2%) | 55 (13.6%) | 169 (32.4%) |
| Regional hospital | 276 (14.1%) | 206 (19.9%) | 2 (0.7%) | 204 (26.8%) | 70 (7.6%) | 1 (0.2%) | 69 (13.2%) |
| Specialized pediatric hospitals | 435 (22.2%) | 167 (16.1%) | 125 (45.3%) | 42 (5.5%) | 268 (29.0%) | 197 (48.9%) | 71 (13.6%) |
| Others | 38 (1.9%) | 12 (1.2%) | 1 (0.4%) | 11 (1.4%) | 26 (2.8%) | 0 | 26 (5.0%) |
| Median length of hospitalization, days (IQR) | 8 (3, 24) | 6 (3, 17) | 24 (10, 67) | 5 (2, 8) | 12 (4, 31) | 22 (8, 53) | 8 (3, 18) |
| Stay in ICU, n (%) | 771 (39.3%) | 339 (32.7%) | 203 (73.6%) | 136 (17.9%) | 432 (46.8%) | 278 (69%) | 154 (29.6%) |
| Median length of stay on an ICU (if patient was admitted to ICU), hours (IQR) | 210 (59, 696) | 199 (51, 708) | 409 (141, 973) | 57 (21, 189) | 216 (66, 664) | 325 (141, 961) | 76 (34, 210) |

# Table S11. Overview of high-risk features mentioned in pulmonary embolism and deep vein thrombosis cases, intensive care unit (ICU) stay, and length of hospitalization, stratified by age and sex.

| **Characteristic** | **Both diseases,** N = 1,961 | **Pulmonary embolism (with or without concomitant deep vein thrombosis)** | | | | | | | **Deep vein thrombosis (excluding cases with concomitant pulmonary embolism diagnosis)** | | | | | | |
| --- | --- | --- | --- | --- | --- | --- | --- | --- | --- | --- | --- | --- | --- | --- | --- |
|  |  | **Overall**, N = 815 | **Female** | | | **Male** | | | **Overall**, N = 1,146 | **Female** | | | **Male** | | |
|  |  |  | **Overall**, N = 497 | **0 to 9 years old**, N = 35 | **10 to 19 years old**, N = 462 | **Overall**, N = 318 | **0 to 9 years old**, N = 46 | **10 to 19 years old**, N = 272 |  | **Overall**, N = 540 | **0 to 9 years old**, N = 241 | **10 to 19 years old**, N = 299 | **Overall**, N = 606 | **0 to 9 years old**, N = 357 | **10 to 19 years old**, N = 249 |
| High-risk features, n (%) | 592 (30.2%) | 165 (20.2%) | 95 (19.1%) | 24 (68.6%) | 71 (15.4%) | 70 (22.0%) | 19 (41.3%) | 51 (18.8%) | 427 (37.3%) | 186 (34.4%) | 132 (54.8%) | 54 (18.1%) | 241 (39.8%) | 198 (55.5%) | 43 (17.3%) |
| Invasive ventilation | 367 (18.7%) | 78 (9.6%) | 42 (8.5%) | 17 (48.6%) | 25 (5.4%) | 36 (11.3%) | 12 (26.1%) | 24 (8.8%) | 289 (25.2%) | 116 (21.5%) | 103 (42.7%) | 13 (4.3%) | 173 (28.5%) | 159 (44.5%) | 14 (5.6%) |
| Use of vasopressors | 202 (10.3%) | 53 (6.5%) | 27 (5.4%) | 8 (22.9%) | 19 (4.1%) | 26 (8.2%) | 11 (23.9%) | 15 (5.5%) | 149 (13.0%) | 58 (10.7%) | 53 (22.0%) | 5 (1.7%) | 91 (15.0%) | 83 (23.2%) | 8 (3.2%) |
| Use of systemic thrombolysis | 150 (7.6%) | 57 (7.0%) | 45 (9.1%) | 7 (20.0%) | 38 (8.2%) | 12 (3.8%) | 4 (8.7%) | 8 (2.9%) | 93 (8.1%) | 51 (9.4%) | 15 (6.2%) | 36 (12.0%) | 42 (6.9%) | 23 (6.4%) | 19 (7.6%) |
| Shock | 141 (7.2%) | 61 (7.5%) | 30 (6.0%) | 7 (20.0%) | 23 (5.0%) | 31 (9.7%) | 9 (19.6%) | 22 (8.1%) | 80 (7.0%) | 36 (6.7%) | 30 (12.4%) | 6 (2.0%) | 44 (7.3%) | 37 (10.4%) | 7 (2.8%) |
| Cardiac arrest | 93 (4.7%) | 33 (4.0%) | 20 (4.0%) | 7 (20.0%) | 13 (2.8%) | 13 (4.1%) | 8 (17.4%) | 5 (1.8%) | 60 (5.2%) | 28 (5.2%) | 24 (10.0%) | 4 (1.3%) | 32 (5.3%) | 27 (7.6%) | 5 (2.0%) |
| Extracorporeal membrane oxygenation | 68 (3.5%) | 23 (2.8%) | 13 (2.6%) | 2 (5.7%) | 11 (2.4%) | 10 (3.1%) | 6 (13.0%) | 4 (1.5%) | 45 (3.9%) | 20 (3.7%) | 14 (5.8%) | 6 (2.0%) | 25 (4.1%) | 20 (5.6%) | 5 (2.0%) |
| Surgical thrombectomy | 13 (0.7%) | 6 (0.7%) | 5 (1.0%) | 2 (5.7%) | 3 (0.6%) | 1 (0.3%) | 0 (0.0%) | 1 (0.4%) | 7 (0.6%) | 3 (0.6%) | 2 (0.8%) | 1 (0.3%) | 4 (0.7%) | 3 (0.8%) | 1 (0.4%) |
| Hospital type, n (%) |  |  |  |  |  |  |  |  |  |  |  |  |  |  |  |
| University hospital | 647 (33.0%) | 245 (30.1%) | 119 (23.9%) | 19 (54.3%) | 100 (21.6%) | 126 (39.6%) | 26 (56.5%) | 100 (36.8%) | 402 (35.1%) | 192 (35.6%) | 99 (41.1%) | 93 (31.1%) | 210 (34.7%) | 124 (34.7%) | 86 (34.5%) |
| Major hospital | 565 (28.8%) | 339 (41.6%) | 229 (46.1%) | 3 (8.6%) | 226 (48.9%) | 110 (34.6%) | 7 (15.2%) | 103 (37.9%) | 226 (19.7%) | 112 (20.7%) | 27 (11.2%) | 85 (28.4%) | 114 (18.8%) | 48 (13.4%) | 66 (26.5%) |
| Regional hospital | 276 (14.1%) | 155 (19.0%) | 123 (24.7%) | 0 (0.0%) | 123 (26.6%) | 32 (10.1%) | 0 (0.0%) | 32 (11.8%) | 121 (10.6%) | 83 (15.4%) | 2 (0.8%) | 81 (27.1%) | 38 (6.3%) | 1 (0.3%) | 37 (14.9%) |
| Specialized pediatric hospitals | 435 (22.2%) | 58 (7.1%) | 21 (4.2%) | 13 (37.1%) | 8 (1.7%) | 37 (11.6%) | 13 (28.3%) | 24 (8.8%) | 377 (32.9%) | 146 (27.0%) | 112 (46.5%) | 34 (11.4%) | 231 (38.1%) | 184 (51.5%) | 47 (18.9%) |
| Others | 38 (1.9%) | 18 (2.2%) | 5 (1.0%) | 0 (0.0%) | 5 (1.1%) | 13 (4.1%) | 0 (0.0%) | 13 (4.8%) | 20 (1.7%) | 7 (1.3%) | 1 (0.4%) | 6 (2.0%) | 13 (2.1%) | 0 (0.0%) | 13 (5.2%) |
| Median length of hospitalization, days (IQR) | 8 (3, 24) | 6 (3, 15) | 5 (3, 9) | 31 (12, 102) | 5 (3, 8) | 10 (4, 22) | 14 (7, 40) | 9 (4, 21) | 11 (3, 32) | 8 (3, 30) | 24 (10, 61) | 5 (2, 9) | 13 (4, 34) | 23 (9, 53) | 6 (2, 15) |
| Stay in ICU, n (%) | 771 (39.3%) | 267 (32.8%) | 127 (25.6%) | 30 (85.7%) | 97 (21.0%) | 140 (44.0%) | 29 (63.0%) | 111 (40.8%) | 504 (44.0%) | 212 (39.3%) | 173 (71.8%) | 39 (13.0%) | 292 (48.2%) | 249 (69.7%) | 43 (17.3%) |
| Median length of stay on an ICU (if patient was admitted to ICU), hours (IQR) | 210 (59, 696) | 75 (27, 279) | 66 (23, 278) | 294 (122, 992) | 44 (20, 116) | 89 (37, 284) | 346 (162, 2,176) | 64 (29, 176) | 316 (97, 842) | 342 (106, 896) | 427 (155, 949) | 112 (27, 525) | 309 (89, 797) | 324 (138, 897) | 91 (66, 455) |

ICU: intensive care unit

# Table S12. Incidence rate (IR) of incident hospital admissions, per 100,000 children and adolescents per year stratified by age and sex.

| **Venous thromboembolism** | | | | | | | | | |
| --- | --- | --- | --- | --- | --- | --- | --- | --- | --- |
|  | **Overall** | | | **Female** | | | **Male** | | |
|  |  | | |  | | |  | | |
| Age (years) | n | Events | IR (95% CI) | n | Events | IR (95% CI) | n | Events | IR (95% CI) |
| 0 years old | 1,605,836 | 351 | 21.9 (19.6; 24.3) | 780,437 | 136 | 17.4 (14.6; 20.6) | 825,401 | 215 | 26 (22.7; 29.8) |
| 1-4 years old | 6,512,171 | 135 | 2.1 (1.7; 2.5) | 3,165,233 | 61 | 1.9 (1.5; 2.5) | 3,346,959 | 74 | 2.2 (1.7; 2.8) |
| 5-9 years old | 8,190,890 | 99 | 1.2 (1; 1.5) | 3,980,099 | 43 | 1.1 (0.8; 1.5) | 4,210,809 | 56 | 1.3 (1; 1.7) |
| 10-14 years old | 8,399,245 | 161 | 1.9 (1.6; 2.2) | 4,085,275 | 67 | 1.6 (1.3; 2.1) | 4,313,995 | 94 | 2.2 (1.8; 2.7) |
| 15-19 years old | 8,771,725 | 907 | 10.3 (9.7; 11) | 4,261,492 | 586 | 13.8 (12.7; 14.9) | 4,510,256 | 321 | 7.1 (6.4; 7.9) |
| Overall | 33,479,867 | 1,653 | 4.9 (4.7; 5.2) | 16,272,536 | 893 | 5.5 (5.1; 5.9) | 17,207,420 | 760 | 4.4 (4.1; 4.7) |
|  |  |  |  |  |  |  |  |  |  |
| **Pulmonary embolism (with or without concomitant deep vein thrombosis)** | | | | | | | | | |
|  | **Overall** | | | **Female** | | | **Male** | | |
| Age (years) | n | Events | IR (95% CI) | n | Events | IR (95% CI) | n | Events | IR (95% CI) |
| 0 years old | 1,605,836 | 28 | 1.7 (1.2; 2.5) | 780,437 | 15 | 1.9 (1.1; 3.2) | 825,401 | 13 | 1.6 (0.8; 2.7) |
| 1-4 years old | 6,512,171 | 18 | 0.3 (0.2; 0.4) | 3,165,233 | 9 | 0.3 (0.1; 0.5) | 3,346,959 | 9 | 0.3 (0.1; 0.5) |
| 5-9 years old | 8,190,890 | 24 | 0.3 (0.2; 0.4) | 3,980,099 | 9 | 0.2 (0.1; 0.4) | 4,210,809 | 15 | 0.4 (0.2; 0.6) |
| 10-14 years old | 8,399,245 | 60 | 0.7 (0.5; 0.9) | 4,085,275 | 26 | 0.6 (0.4; 0.9) | 4,313,995 | 34 | 0.8 (0.5; 1.1) |
| 15-19 years old | 8,771,725 | 581 | 6.6 (6.1; 7.2) | 4,261,492 | 386 | 9.1 (8.2; 10) | 4,510,256 | 195 | 4.3 (3.7; 5) |
| Overall | 33,479,867 | 711 | 2.1 (2; 2.3) | 16,272,536 | 445 | 2.7 (2.5; 3) | 17,207,420 | 266 | 1.5 (1.4; 1.7) |
|  |  |  |  |  |  |  |  |  |  |
| **Deep vein thrombosis (excluding cases with concomitant pulmonary embolism diagnosis)** | | | | | | | | | |
|  | **Overall** | | | **Female** | | | **Male** | | |
| Age (years) | n | Events | IR (95% CI) | n | Events | IR (95% CI) | n | Events | IR (95% CI) |
| 0 years old | 1,605,836 | 323 | 20.1 (18; 22.4) | 780,437 | 121 | 15.5 (12.9; 18.5) | 825,401 | 202 | 24.5 (21.2; 28.1) |
| 1-4 years old | 6,512,171 | 117 | 1.8 (1.5; 2.2) | 3,165,233 | 52 | 1.6 (1.2; 2.2) | 3,346,959 | 65 | 1.9 (1.5; 2.5) |
| 5-9 years old | 8,190,890 | 75 | 0.9 (0.7; 1.1) | 3,980,099 | 34 | 0.9 (0.6; 1.2) | 4,210,809 | 41 | 1 (0.7; 1.3) |
| 10-14 years old | 8,399,245 | 101 | 1.2 (1; 1.5) | 4,085,275 | 41 | 1 (0.7; 1.4) | 4,313,995 | 60 | 1.4 (1.1; 1.8) |
| 15-19 years old | 8,771,725 | 326 | 3.7 (3.3; 4.1) | 4,261,492 | 200 | 4.7 (4.1; 5.4) | 4,510,256 | 126 | 2.8 (2.3; 3.3) |
| Overall | 33,479,867 | 942 | 2.8 (2.6; 3) | 16,272,536 | 448 | 2.8 (2.5; 3) | 17,207,420 | 494 | 2.9 (2.6; 3.1) |

# Table S13. Incidence rate (IR) of disease-related hospital admissions, per 100,000 children and adolescents per year stratified by age and sex.

| **Venous thromboembolism** | | | | | | | | | |
| --- | --- | --- | --- | --- | --- | --- | --- | --- | --- |
|  | **Overall** | | | **Female** | | | **Male** | | |
|  |  | | |  | | |  | | |
| Age (years) | n | Events | IR (95% CI) | n | Events | IR (95% CI) | n | Events | IR (95% CI) |
| 0 years old | 1,605,836 | 393 | 24.5 (22.1; 27) | 780,437 | 148 | 19 (16; 22.3) | 825,401 | 245 | 29.7 (26.1; 33.6) |
| 1-4 years old | 6,512,171 | 166 | 2.5 (2.2; 3) | 3,165,233 | 70 | 2.2 (1.7; 2.8) | 3,346,959 | 96 | 2.9 (2.3; 3.5) |
| 5-9 years old | 8,190,890 | 120 | 1.5 (1.2; 1.8) | 3,980,099 | 58 | 1.5 (1.1; 1.9) | 4,210,809 | 62 | 1.5 (1.1; 1.9) |
| 10-14 years old | 8,399,245 | 200 | 2.4 (2.1; 2.7) | 4,085,275 | 88 | 2.2 (1.7; 2.7) | 4,313,995 | 112 | 2.6 (2.1; 3.1) |
| 15-19 years old | 8,771,725 | 1,082 | 12.3 (11.6; 13.1) | 4,261,492 | 673 | 15.8 (14.6; 17) | 4,510,256 | 409 | 9.1 (8.2; 10) |
| Overall | 33,479,867 | 1,961 | 5.9 (5.6; 6.1) | 16,272,536 | 1,037 | 6.4 (6; 6.8) | 17,207,420 | 924 | 5.4 (5; 5.7) |
|  |  |  |  |  |  |  |  |  |  |
| **Pulmonary embolism (with or without concomitant deep vein thrombosis)** | | | | | | | | | |
|  | **Overall** | | | **Female** | | | **Male** | | |
| Age (years) | n | Events | IR (95% CI) | n | Events | IR (95% CI) | n | Events | IR (95% CI) |
| 0 years old | 1,605,836 | 29 | 1.8 (1.2; 2.6) | 780,437 | 15 | 1.9 (1.1; 3.2) | 825,401 | 14 | 1.7 (1; 2.8) |
| 1-4 years old | 6,512,171 | 25 | 0.4 (0.2; 0.6) | 3,165,233 | 10 | 0.3 (0.2; 0.6) | 3,346,959 | 15 | 0.4 (0.3; 0.7) |
| 5-9 years old | 8,190,890 | 27 | 0.3 (0.2; 0.5) | 3,980,099 | 10 | 0.3 (0.1; 0.5) | 4,210,809 | 17 | 0.4 (0.2; 0.6) |
| 10-14 years old | 8,399,245 | 68 | 0.8 (0.6; 1) | 4,085,275 | 32 | 0.8 (0.5; 1.1) | 4,313,995 | 36 | 0.8 (0.6; 1.2) |
| 15-19 years old | 8,771,725 | 666 | 7.6 (7; 8.2) | 4,261,492 | 330 | 10.1 (9.2; 11.1) | 4,510,256 | 236 | 5.2 (4.6; 5.9) |
| Overall | 33,479,867 | 815 | 2.4 (2.3; 2.6) | 16,272,536 | 497 | 3.1 (2.8; 3.3) | 17,207,420 | 318 | 1.8 (1.7; 2.1) |
|  |  |  |  |  |  |  |  |  |  |
| **Deep vein thrombosis (excluding cases with concomitant pulmonary embolism diagnosis)** | | | | | | | | | |
|  | **Overall** | | | **Female** | | | **Male** | | |
| Age (years) | n | Events | IR (95% CI) | n | Events | IR (95% CI) | n | Events | IR (95% CI) |
| 0 years old | 1,605,836 | 364 | 22.7 (20.4; 25.1) | 780,437 | 231 | 17 (14.3; 20.2) | 825,401 | 133 | 28 (24.5; 31.8) |
| 1-4 years old | 6,512,171 | 141 | 2.2 (1.8; 2.6) | 3,165,233 | 81 | 1.9 (1.4; 2.4) | 3,346,959 | 60 | 2.4 (1.9; 3) |
| 5-9 years old | 8,190,890 | 93 | 1.1 (0.9; 1.4) | 3,980,099 | 45 | 1.2 (0.9; 1.6) | 4,210,809 | 48 | 1.1 (0.8; 1.4) |
| 10-14 years old | 8,399,245 | 132 | 1.6 (1.3; 1.9) | 4,085,275 | 76 | 1.4 (1; 1.8) | 4,313,995 | 56 | 1.8 (1.4; 2.2) |
| 15-19 years old | 8,771,725 | 416 | 4.7 (4.3; 5.2) | 4,261,492 | 173 | 5.7 (5; 6.5) | 4,510,256 | 243 | 3.8 (3.3; 4.5) |
| Overall | 33,479,867 | 1,146 | 3.4 (3.2; 3.6) | 16,272,536 | 540 | 3.3 (3; 3.6) | 17,207,420 | 606 | 3.5 (3.2; 3.8) |

# Table S14. Proportion of hospitalizations (PH) of disease-related hospital admissions, per 10,000 hospital admissions stratified by age and sex.

| **Venous thromboembolism** | | | | | | | | | |
| --- | --- | --- | --- | --- | --- | --- | --- | --- | --- |
|  | **Overall** | | | **Female** | | | **Male** | | |
| Age (years) | n | Events | PH (95% CI) | n | Events | PH (95% CI) | n | Events | PH (95% CI) |
| 0 years old | 2,014,821 | 393 | 2.0 (1.8; 2.2) | 959,250 | 148 | 1.5 (1.3; 1.8) | 1,055,571 | 245 | 2.3 (2.0; 2.6) |
| 1-4 years old | 408,490 | 166 | 4.1 (3.5; 4.7) | 169,886 | 70 | 4.1 (3.3; 5.2) | 238,604 | 96 | 4.0 (3.3; 4.9) |
| 5-9 years old | 353,956 | 120 | 3.4 (2.8; 4.1) | 142,393 | 58 | 4.1 (3.2; 5.3) | 211,563 | 62 | 2.9 (2.3; 3.8) |
| 10-14 years old | 368,666 | 200 | 5.4 (4.7; 6.2) | 170,981 | 88 | 5.1 (4.2; 6.3) | 197,685 | 112 | 5.7 (4.7; 6.8) |
| 15-19 years old | 701,056 | 1,082 | 15.4 (14.5; 16.4) | 357,228 | 673 | 18.8 (17.5; 20.3) | 343,828 | 409 | 11.9 (10.8; 13.1) |
| Overall | 3,846,989 | 1,961 | 5.1 (4.9; 5.3) | 1,799,738 | 1,037 | 5.8 (5.4; 6.1) | 2,047,251 | 924 | 4.5 (4.2; 4.8) |
|  |  |  |  |  |  |  |  |  |  |
| **Pulmonary embolism (with or without concomitant deep vein thrombosis)** | | | | | | | | | |
|  | **Overall** | | | **Female** | | | **Male** | | |
| Age (years) | n | Events | PH (95% CI) | n | Events | PH (95% CI) | n | Events | PH (95% CI) |
| 0 years old | 2,014,821 | 29 | 0.1 (0.1; 0.2) | 959,250 | 15 | 0.2 (0.1; 0.3) | 1,055,571 | 14 | 0.1 (0.1; 0.2) |
| 1-4 years old | 408,490 | 25 | 0.6 (0.4; 0.9) | 169,886 | 10 | 0.6 (0.3; 1.1) | 238,604 | 15 | 0.6 (0.4; 1.0) |
| 5-9 years old | 353,956 | 27 | 0.8 (0.5; 1.1) | 142,393 | 10 | 0.7 (0.4; 1.3) | 211,563 | 17 | 0.8 (0.5; 1.3) |
| 10-14 years old | 368,666 | 68 | 1.8 (1.5; 2.3) | 170,981 | 32 | 1.9 (1.3; 2.6) | 197,685 | 36 | 1.8 (1.3; 2.5) |
| 15-19 years old | 701,056 | 666 | 9.5 (8.8; 10.2) | 357,228 | 330 | 12.0 (11.0; 13.2) | 343,828 | 236 | 6.9 (6.0; 7.8) |
| Overall | 3,846,989 | 815 | 2.1 (2.0; 2.3) | 1,799,738 | 497 | 2.8 (2.5; 3.0) | 2,047,251 | 318 | 1.6 (1.4; 1.7) |
|  |  |  |  |  |  |  |  |  |  |
| **Deep vein thrombosis (excluding cases with concomitant pulmonary embolism diagnosis)** | | | | | | | | | |
|  | **Overall** | | | **Female** | | | **Male** | | |
| Age (years) | n | Events | PH (95% CI) | n | Events | PH (95% CI) | n | Events | PH (95% CI) |
| 0 years old | 2,014,821 | 364 | 1.8 (1.6; 2.0) | 959,250 | 231 | 1.4 (1.2; 1.6) | 1,055,571 | 133 | 2.2 (1.9; 2.5) |
| 1-4 years old | 408,490 | 141 | 3.5 (2.9; 4.1) | 169,886 | 81 | 3.5 (2.7; 4.5) | 238,604 | 60 | 3.4 (2.7; 4.2) |
| 5-9 years old | 353,956 | 93 | 2.6 (2.1; 3.2) | 142,393 | 45 | 3.4 (2.5; 4.5) | 211,563 | 48 | 2.1 (1.6; 2.8) |
| 10-14 years old | 368,666 | 132 | 3.6 (3.0; 4.2) | 170,981 | 76 | 3.3 (2.5; 4.3) | 197,685 | 56 | 3.8 (3.1; 4.8) |
| 15-19 years old | 701,056 | 416 | 5.9 (5.4; 6.5) | 357,228 | 173 | 6.8 (6.0; 7.7) | 343,828 | 243 | 5.0 (4.3; 5.8) |
| Overall | 3,846,989 | 1,146 | 3.0 (2.8; 3.2) | 1,799,738 | 540 | 3.0 (2.8; 3.3) | 2,047,251 | 606 | 3.0 (2.7; 3.2) |

# Table S15. Incidence rate (IR) of incident hospital admissions, per 100,000 children and adolescents stratified by clinically selected age groups and sex.

| **Venous thromboembolism** | | | | | | | | | |
| --- | --- | --- | --- | --- | --- | --- | --- | --- | --- |
|  | **Overall** | | | **Female** | | | **Male** | | |
| Age (years) | n | Events | IR (95% CI) | n | Events | IR (95% CI) | n | Events | IR (95% CI) |
| 0 years old | 1,605,836 | 351 | 21.9 (19.6; 24.3) | 780,437 | 136 | 17.4 (14.6; 20.6) | 825,401 | 215 | 26 (22.7; 29.8) |
| 1-5 years old | 8,143,607 | 150 | 1.8 (1.6; 2.2) | 3,957,745 | 69 | 1.7 (1.4; 2.2) | 4,185,889 | 81 | 1.9 (1.5; 2.4) |
| 6-12 years old | 11,561,255 | 148 | 1.3 (1.1; 1.5) | 5,620,544 | 57 | 1 (0.8; 1.3) | 5,940,742 | 91 | 1.5 (1.2; 1.9) |
| 13-17 years old | 8,592,537 | 514 | 6 (5.5; 6.5) | 4,175,734 | 311 | 7.4 (6.6; 8.3) | 4,416,824 | 203 | 4.6 (4; 5.3) |
| Overall | 29,903,235 | 1,163 | 3.9 (3.7; 4.1) | 14,534,460 | 573 | 3.9 (3.6; 4.3) | 15,368,856 | 590 | 3.8 (3.5; 4.2) |
|  |  |  |  |  |  |  |  |  |  |
| **Pulmonary embolism (with or without concomitant deep vein thrombosis)** | | | | | | | | | |
|  | **Overall** | | | **Female** | | | **Male** | | |
| Age (years) | n | Events | IR (95% CI) | n | Events | IR (95% CI) | n | Events | IR (95% CI) |
| 0 years old | 1,605,836 | 28 | 1.7 (1.2; 2.5) | 780,437 | 15 | 1.9 (1.1; 3.2) | 825,401 | 13 | 1.6 (0.8; 2.7) |
| 1-5 years old | 8,143,607 | 19 | 0.2 (0.1; 0.4) | 3,957,745 | 9 | 0.2 (0.1; 0.4) | 4,185,889 | 10 | 0.2 (0.1; 0.4) |
| 6-12 years old | 11,561,255 | 48 | 0.4 (0.3; 0.6) | 5,620,544 | 20 | 0.4 (0.2; 0.5) | 5,940,742 | 28 | 0.5 (0.3; 0.7) |
| 13-17 years old | 8,592,537 | 280 | 3.3 (2.9; 3.7) | 4,175,734 | 179 | 4.3 (3.7; 5) | 4,416,824 | 101 | 2.3 (1.9; 2.8) |
| Overall | 29,903,235 | 375 | 1.3 (1.1; 1.4) | 14,534,460 | 223 | 1.5 (1.3; 1.7) | 15,368,856 | 152 | 1 (0.8; 1.2) |
|  |  |  |  |  |  |  |  |  |  |
| **Deep vein thrombosis (excluding cases with concomitant pulmonary embolism diagnosis)** | | | | | | | | | |
|  | **Overall** | | | **Female** | | | **Male** | | |
| Age (years) | n | Events | IR (95% CI) | n | Events | IR (95% CI) | n | Events | IR (95% CI) |
| 0 years old | 1,605,836 | 323 | 20.1 (18; 22.4) | 780,437 | 121 | 15.5 (12.9; 18.5) | 825,401 | 202 | 24.5 (21.2; 28.1) |
| 1-5 years old | 8,143,607 | 131 | 1.6 (1.3; 1.9) | 3,957,745 | 60 | 1.5 (1.2; 2) | 4,185,889 | 71 | 1.7 (1.3; 2.1) |
| 6-12 years old | 11,561,255 | 100 | 0.9 (0.7; 1.1) | 5,620,544 | 37 | 0.7 (0.5; 0.9) | 5,940,742 | 63 | 1.1 (0.8; 1.4) |
| 13-17 years old | 8,592,537 | 234 | 2.7 (2.4; 3.1) | 4,175,734 | 132 | 3.2 (2.6; 3.7) | 4,416,824 | 102 | 2.3 (1.9; 2.8) |
| Overall | 29,903,235 | 788 | 2.6 (2.5; 2.8) | 14,534,460 | 350 | 2.4 (2.2; 2.7) | 15,368,856 | 438 | 2.8 (2.6; 3.1) |

# Table S16. Incidence rate (IR) of disease-related hospital admissions, per 100,000 children and adolescents stratified by clinically selected age groups and sex.

| **Venous thromboembolism** | | | | | | | | | |
| --- | --- | --- | --- | --- | --- | --- | --- | --- | --- |
|  | **Overall** | | | **Female** | | | **Male** | | |
| Age (years) | n | Events | IR (95% CI) | n | Events | IR (95% CI) | n | Events | IR (95% CI) |
| 0 years old | 1,605,836 | 393 | 24.5 (22.1; 27) | 780,437 | 148 | 19 (16; 22.3) | 825,401 | 245 | 29.7 (26.1; 33.6) |
| 1-5 years old | 8,143,607 | 184 | 2.3 (1.9; 2.6) | 3,957,745 | 80 | 2 (1.6; 2.5) | 4,185,889 | 104 | 2.5 (2; 3) |
| 6-12 years old | 11,561,255 | 177 | 1.5 (1.3; 1.8) | 5,620,544 | 75 | 1.3 (1; 1.7) | 5,940,742 | 102 | 1.7 (1.4; 2.1) |
| 13-17 years old | 8,592,537 | 636 | 7.4 (6.8; 8) | 4,175,734 | 377 | 9 (8.1; 10) | 4,416,824 | 259 | 5.9 (5.2; 6.6) |
| Overall | 29,903,235 | 1,390 | 4.6 (4.4; 4.9) | 14,534,460 | 680 | 4.7 (4.3; 5.0) | 15,368,856 | 710 | 4.6 (4.3; 5.0) |
|  |  |  |  |  |  |  |  |  |  |
| **Pulmonary embolism (with or without concomitant deep vein thrombosis)** | | | | | | | | | |
|  | **Overall** | | | **Female** | | | **Male** | | |
| Age (years) | n | Events | IR (95% CI) | n | Events | IR (95% CI) | n | Events | IR (95% CI) |
| 0 years old | 1,605,836 | 29 | 1.8 (1.2; 2.6) | 780,437 | 15 | 1.9 (1.1; 3.2) | 825,401 | 14 | 1.7 (0.9; 2.8) |
| 1-5 years old | 8,143,607 | 26 | 0.3 (0.2; 0.5) | 3,957,745 | 10 | 0.3 (0.1; 0.5) | 4,185,889 | 16 | 0.4 (0.2; 0.6) |
| 6-12 years old | 11,561,255 | 55 | 0.5 (0.4; 0.6) | 5,620,544 | 23 | 0.4 (0.3; 0.6) | 5,940,742 | 32 | 0.5 (0.4; 0.8) |
| 13-17 years old | 8,592,537 | 322 | 3.7 (3.3; 4.2) | 4,175,734 | 207 | 5 (4.3; 5.7) | 4,416,824 | 115 | 2.6 (2.1; 3.1) |
| Overall | 29,903,235 | 432 | 1.4 (1.3; 1.6) | 14,534,460 | 255 | 1.8 (1.5; 2.0) | 15,368,856 | 177 | 1.2 (1.0; 1.3) |
|  |  |  |  |  |  |  |  |  |  |
| **Deep vein thrombosis (excluding cases with concomitant pulmonary embolism diagnosis)** | | | | | | | | | |
|  | **Overall** | | | **Female** | | | **Male** | | |
| Age (years) | n | Events | IR (95% CI) | n | Events | IR (95% CI) | n | Events | IR (95% CI) |
| 0 years old | 1,605,836 | 364 | 22.7 (20.4; 25.1) | 780,437 | 133 | 17 (14.3; 20.2) | 825,401 | 231 | 28 (24.5; 31.8) |
| 1-5 years old | 8,143,607 | 158 | 1.9 (1.6; 2.3) | 3,957,745 | 70 | 1.8 (1.4; 2.2) | 4,185,889 | 88 | 2.1 (1.7; 2.6) |
| 6-12 years old | 11,561,255 | 122 | 1.1 (0.9; 1.3) | 5,620,544 | 52 | 0.9 (0.7; 1.2) | 5,940,742 | 70 | 1.2 (0.9; 1.5) |
| 13-17 years old | 8,592,537 | 314 | 3.7 (3.3; 4.1) | 4,175,734 | 170 | 4.1 (3.5; 4.7) | 4,416,824 | 144 | 3.3 (2.7; 3.8) |
| Overall | 29,903,235 | 958 | 3.2 (3.0; 3.4) | 14,534,460 | 425 | 2.9 (2.7; 3.2) | 15,368,856 | 533 | 3.5 (3.2; 3.8) |

# Table S17. Proportion of hospitalizations (PH) of disease-related hospital admissions, per 10,000 hospital admissions stratified by clinically selected age groups and sex.

| **Venous thromboembolism** | | | | | | | | | |
| --- | --- | --- | --- | --- | --- | --- | --- | --- | --- |
|  | **Overall** | | | **Female** | | | **Male** | | |
| Age (years) | n | Events | PH (95% CI) | n | Events | PH (95% CI) | n | Events | PH (95% CI) |
| 0 years old | 2,014,821 | 393 | 2.0 (1.8; 2.2) | 959,250 | 148 | 1.5 (1.3; 1.8) | 1,055,571 | 245 | 2.3 (2.0; 2.6) |
| 1-5 years old | 494,579 | 184 | 3.7 (3.2; 4.3) | 204,345 | 80 | 3.9 (3.1; 4.9) | 290,234 | 104 | 3.6 (3; 4.3) |
| 6-12 years old | 455,234 | 177 | 3.9 (3.4; 4.5) | 190,479 | 75 | 3.9 (3.1; 4.9) | 264,755 | 102 | 3.9 (3.2; 4.7) |
| 13-17 years old | 566,087 | 636 | 11.2 (10.4; 12.1) | 285,852 | 377 | 13.2 (11.9; 14.6) | 280,235 | 259 | 9.2 (8.2; 10.4) |
| Overall | 3,530,721 | 1,390 | 3.9 (3.7; 4.1) | 1,639,926 | 680 | 4.1 (3.8; 4.5) | 1,890,795 | 710 | 3.8 (3.5; 4.0) |
|  |  |  |  |  |  |  |  |  |  |
| **Pulmonary embolism (with or without concomitant deep vein thrombosis)** | | | | | | | | | |
|  | **Overall** | | | **Female** | | | **Male** | | |
| Age (years) | n | Events | PH (95% CI) | n | Events | PH (95% CI) | n | Events | PH (95% CI) |
| 0 years old | 2,014,821 | 29 | 0.1 (0.1; 0.2) | 959,250 | 15 | 0.2 (0.1; 0.3) | 1,055,571 | 14 | 0.1 (0.1; 0.2) |
| 1-5 years old | 494,579 | 26 | 0.5 (0.4; 0.8) | 204,345 | 10 | 0.5 (0.3; 0.9) | 290,234 | 16 | 0.6 (0.3; 0.9) |
| 6-12 years old | 455,234 | 55 | 1.2 (0.9; 1.6) | 190,479 | 23 | 1.2 (0.8; 1.8) | 264,755 | 32 | 1.2 (0.9; 1.7) |
| 13-17 years old | 566,087 | 322 | 5.7 (5.1; 6.3) | 285,852 | 207 | 7.2 (6.3; 8.3) | 280,235 | 115 | 4.1 (3.4; 4.9) |
| Overall | 3,530,721 | 432 | 1.2 (1.1; 1.3) | 1,639,926 | 255 | 1.6 (1.4; 1.7) | 1,890,795 | 177 | 0.9 (0.8; 1.1) |
|  |  |  |  |  |  |  |  |  |  |
| **Deep vein thrombosis (excluding cases with concomitant pulmonary embolism diagnosis)** | | | | | | | | | |
|  | **Overall** | | | **Female** | | | **Male** | | |
| Age (years) | n | Events | PH (95% CI) | n | Events | PH (95% CI) | n | Events | PH (95% CI) |
| 0 years old | 2,014,821 | 364 | 1.8 (1.6; 2.0) | 959,250 | 133 | 1.4 (1.2; 1.6) | 1,055,571 | 231 | 2.2 (1.9; 2.5) |
| 1-5 years old | 494,579 | 158 | 3.2 (2.7; 3.7) | 204,345 | 70 | 3.4 (2.7; 4.3) | 290,234 | 88 | 3.0 (2.5; 3.7) |
| 6-12 years old | 455,234 | 122 | 2.7 (2.2; 3.2) | 190,479 | 52 | 2.7 (2.1; 3.6) | 264,755 | 70 | 2.6 (2.1; 3.3) |
| 13-17 years old | 566,087 | 314 | 5.5 (5; 6.2) | 285,852 | 170 | 5.9 (5.1; 6.9) | 280,235 | 144 | 5.1 (4.4; 6.0) |
| Overall | 3,530,721 | 958 | 2.7 (2.5; 2.9) | 1,639,926 | 425 | 2.6 (2.4; 2.8) | 1,890,795 | 533 | 2.8 (2.6; 3.1) |

# Table S18. In-hospital case fatality rate (CFR), per 100 disease-related hospital admissions stratified by age.

| **Venous thromboembolism** | | | | | | | | | |
| --- | --- | --- | --- | --- | --- | --- | --- | --- | --- |
|  | **Overall** | | | **Female** | | | **Male** | | |
| Age (years) | n | Events | CFR (95% CI) | n | Events | CFR (95% CI) | n | Events | CFR (95% CI) |
| 0 years old | 393 | 43 | 10.9% (8.2; 14.4) | 148 | 19 | 12.8% (8.4; 19.2) | 245 | 24 | 9.8% (6.7; 14.2) |
| 1-4 years old | 166 | 6 | 3.6% (1.7; 7.7) | 70 | 2 | 2.9% (0.8; 9.8) | 96 | 4 | 4.2% (1.6; 10.2) |
| 5-9 years old | 120 | 3 | 2.5% (0.9; 7.1) | 58 | 3 | 5.2% (1.8; 14.1) | 62 | 0 | 0 |
| 10-14 years old | 200 | 7 | 3.5% (1.7; 7.0) | 88 | 4 | 4.5% (1.8; 11.1) | 112 | 3 | 2.7% (0.9; 7.6) |
| 15-19 years old | 1,082 | 20 | 1.8% (1.2; 2.8) | 673 | 13 | 1.9% (1.1; 3.3) | 409 | 7 | 1.7% (0.8; 3.5) |
| Overall | 1,961 | 79 | 4.0% (3.2; 5.0) | 1,037 | 41 | 4.0% (2.9; 5.3) | 924 | 38 | 4.1% (3.0; 5.6) |
|  |  | | |  | | |  | | |
| **Pulmonary embolism (with or without concomitant deep vein thrombosis)** | | | | | | | | | |
|  | **Overall** | | | | | | | | |
| Age (years) | n | | | Events | | | CFR (95% CI) | | |
| 0 years old | 29 | | | 13 | | | 44.8% (28.4; 62.5) | | |
| 1-4 years old | 25 | | | 1 | | | 4.0% (0.7; 19.5) | | |
| 5-9 years old | 27 | | | 1 | | | 3.7% (0.7; 18.3) | | |
| 10-14 years old | 68 | | | 3 | | | 4.4% (1.5; 12.2) | | |
| 15-19 years old | 666 | | | 17 | | | 2.6% (1.6; 4) | | |
| Overall | 815 | | | 35 | | | 4.3% (3.1; 5.9) | | |
|  |  | | |  | | |  | | |
| **Deep vein thrombosis (excluding cases with concomitant pulmonary embolism diagnosis)** | | | | | | | | | |
|  | **Overall** | | | | | | | | |
| Age (years) | n | | | Events | | | CFR (95% CI) | | |
| 0 years old | 201 | | | 30 | | | 8.2% (5.8; 11.5) | | |
| 1-4 years old | 85 | | | 5 | | | 3.5% (1.5; 8.0) | | |
| 5-9 years old | 70 | | | 2 | | | 2.2% (0.6; 7.5) | | |
| 10-14 years old | 105 | | | 4 | | | 3.0% (1.2; 7.5) | | |
| 15-19 years old | 379 | | | 3 | | | 0.7% (0.2; 2.1) | | |
| Overall | 1,146 | | | 44 | | | 3.8% (2.9; 5.1) | | |

Sex stratification was only performed for venous thromboembolism due to low number of disease-related deaths.

# Table S19. In-hospital case fatality rate (CFR), per 100 disease-related hospital admissions stratified by clinically selected age groups.

| **Venous thromboembolism** | | | | | | | | | |
| --- | --- | --- | --- | --- | --- | --- | --- | --- | --- |
|  | **Overall** | | | **Female** | | | **Male** | | |
| Age (years) | n | Events | CFR (95% CI) | n | Events | CFR (95% CI) | n | Events | CFR (95% CI) |
| 0 years old | 393 | 43 | 10.9% (8.2; 14.4) | 148 | 19 | 12.8% (8.4; 19.2) | 245 | 24 | 9.8% (6.7; 14.2) |
| 1-5 years old | 184 | 6 | 3.3% (1.5; 6.9) | 80 | 2 | 3.8% (1.5; 9.5) | 104 | 4 | 2.5% (0.7; 8.7) |
| 6-12 years old | 177 | 6 | 3.4% (1.6; 7.2) | 75 | 5 | 1% (0.2; 5.3) | 102 | 1 | 6.7% (2.9; 14.7) |
| 13-17 years old | 636 | 14 | 2.2% (1.3; 3.7) | 377 | 8 | 2.3% (1.1; 5) | 259 | 6 | 2.1% (1.1; 4.1) |
| Overall | 1,390 | 69 | 4.9% (3.9; 6.2) | 680 | 34 | 5.0% (3.6; 6.9) | 710 | 35 | 4.9% (3.6; 6.8) |
|  |  | | |  | | |  | | |
| **Pulmonary embolism (with or without concomitant deep vein thrombosis)** | | | | | | | | | |
|  | **Overall** | | | | | | | | |
| Age (years) | n | | | Events | | | CFR (95% CI) | | |
| 0 years old | 29 | | | 13 | | | 44.8% (28.4; 62.5) | | |
| 1-5 years old | 26 | | | 1 | | | 3.8% (0.7; 18.9) | | |
| 6-12 years old | 55 | | | 2 | | | 3.6% (1; 12.3) | | |
| 13-17 years old | 322 | | | 10 | | | 3.1% (1.7; 5.6) | | |
| Overall | 432 | | | 26 | | | 6.0% (4.1; 8.7) | | |
|  |  | | |  | | |  | | |
| **Deep vein thrombosis (excluding cases with concomitant pulmonary embolism diagnosis)** | | | | | | | | | |
|  | **Overall** | | | | | | | | |
| Age (years) | n | | | Events | | | CFR (95% CI) | | |
| 0 years old | 364 | | | 30 | | | 8.2% (5.8; 11.5) | | |
| 1-5 years old | 158 | | | 5 | | | 3.2% (1.4; 7.2) | | |
| 6-12 years old | 122 | | | 4 | | | 3.3% (1.3; 8.1) | | |
| 13-17 years old | 314 | | | 4 | | | 1.3% (0.5; 3.2) | | |
| Overall | 958 | | | 43 | | | 4.5% (3.3; 6.0) | | |

Sex stratification was only performed for venous thromboembolism due to low number of disease-related deaths.

# Table S20. In-hospital case fatality rate (CFR) stratified by sex and presence of high-risk features.

| **Venous thromboembolism** | | | | | | | | | |
| --- | --- | --- | --- | --- | --- | --- | --- | --- | --- |
|  | Overall (N=1,961) | | | Female (N=1,037) | | | Male (N=924) | | |
|  | Deaths | Prevalence | CFR (95% CI) | Deaths | Prevalence | CFR (95% CI) | Deaths | Prevalence | CFR (95% CI) |
| Invasive ventilation | 52 | 18.7% | 14.2% (11.0; 18.1) | 29 | 15.2% | 7.0% (3.9; 12.0) | 23 | 22.6% | 19.6% (14.8; 25.5) |
| Shock | 33 | 7.2% | 23.4% (17.2; 31.0) | 18 | 6.4% | 24.2% (15.5; 35.8) | 15 | 8.1% | 22.7% (14.7; 33.3) |
| Cardiac arrest or resuscitation | 34 | 4.7% | 36.6% (27.5; 46.7) | 19 | 4.6% | 22.9% (13.3; 36.5) | 15 | 4.9% | 51.1% (37.0; 65.0) |
| Use of systemic thrombolysis | 18 | 7.6% | 12.0% (7.7; 18.2) | 11 | 9.3% | 5.2 (2.2; 11.6) | 7 | 5.8% | 24.1% (14.6; 36.9) |
| Use of vasopressors | 34 | 10.3% | 16.8% (12.3; 22.6) | 13 | 8.2% | 9.4% (4.8; 17.5) | 21 | 12.7% | 22.2% (15.6; 30.6) |
| ECMO | 26 | 3.5% | 38.2% (27.6; 50.1) | 13 | 3.2% | 39.4% (24.7; 56.3) | 13 | 3.8% | 37.1% (23.2; 53.7) |
| Surgical thrombectomy | 2 | 0.7% | 15.4% (4.3; 42.2) | 2 | 0.8% | 12.5% (2.2; 47.1) | 0 | 0.5% | 20.0% (3.6; 62.4) |
| Any oft he above | 70 | 30.2% | 11.8% (9.5; 14.7) | 36 | 27.1% | 7.5% (4.9; 11.2) | 34 | 33.7% | 15.8% (12.1; 20.2) |
| None of the above | 9 | 69.8% | 0.7% (0.3; 1.2) | 5 | 72.9% | 0.8% (0.4; 1.7) | 4 | 66.3% | 0.5% (0.2; 1.4) |
|  |  |  |  |  |  |  |  |  |  |
| Catheter-directed treatment | 4 | 3.9% | 5.2% (2.0; 12.6) | 2 | 5.0% | 3.8% (1.1; 13.0) | 2 | 2.7% | 8% (2.2; 25.0) |
| Placement of an inferior vena cava filter | 0 | 1.6% | 0 | 0 | 1.4% | 0 | 0 | 1.8% | 0 |
|  |  |  |  |  |  |  |  |  |  |
| **Pulmonary embolism (with or without concomitant deep vein thrombosis)** | | | | | | | | | |
|  | Overall (N=815) | | | Female (N=497) | | | Male (N=318) | | |
|  | Deaths | Prevalence | CFR (95% CI) | Deaths | Prevalence | CFR (95% CI) | Deaths | Prevalence | CFR (95% CI) |
| Invasive ventilation | 20 | 4.0% | 25.6% (17.3; 36.3) | 15 | 4.0% | 35.7% (23; 8.4) | 5 | 4.1% | 13.9% (6.1; 9.6) |
| Shock | 17 | 7.5% | 27.9% (18.2; 40.2) | 9 | 6.0% | 30% (16.7; 11.4) | 8 | 9.7% | 25.8% (13.7; 11) |
| Cardiac arrest or resuscitation | 15 | 6.5% | 45.5% (29.8; 62) | 10 | 5.4% | 50% (29.9; 16.1) | 5 | 8.2% | 38.5% (17.7; 22.8) |
| Use of systemic thrombolysis | 13 | 7.0% | 22.8% (13.8; 35.2) | 10 | 9.1% | 22.2% (12.5; 7.9) | 3 | 3.8% | 25% (8.9; 24.2) |
| Use of vasopressors | 13 | 9.6% | 24.5% (14.9; 37.6) | 5 | 8.5% | 18.5% (8.2; 12.5) | 8 | 11.3% | 30.8% (16.5; 12.9) |
| ECMO | 11 | 2.8% | 47.8% (29.2; 67) | 6 | 2.6% | 46.2% (23.2; 22.8) | 5 | 3.1% | 50% (23.7; 27.8) |
| Surgical thrombectomy | 2 | 0.7% | 33.3% (9.7; 70) | 2 | 1.0% | 40% (11.8; 43.4) | 0 | 0.3% | 0 |
| Any of the above | 30 | 20.2% | 18.2% (13; 24.8) | 19 | 19.1% | 20% (13.2; 3.9) | 11 | 22.0% | 15.7% (9; 5.2) |
| None of the above | 5 | 79.8% | 0.8% (0.3; 1.8) | 2 | 80.9% | 0.5% (0.1; 0.9) | 3 | 78.0% | 1.2% (0.4; 1.5) |
|  |  |  |  |  |  |  |  |  |  |
| Catheter-directed treatment | 4 | 3.3% | 14.8% (5.9; 32.5) | 2 | 4.2% | 9.5% (2.7; 28.9) | 2 | 1.9% | 33.3% (9.7; 70.0) |
| Placement of an inferior vena cava filter | 0 | 2.7% | 0 | 0 | 2.0% | 0 | 0 | 3.8% | 0 |
|  |  |  |  |  |  |  |  |  |  |
| **Deep vein thrombosis (excluding cases with concomitant pulmonary embolism diagnosis)** | | | | | | | | | |
|  | Overall (N=1,146) | | | Female (N=540) | | | Male (N=606) | | |
|  | Deaths | Prevalence | CFR (95% CI) | Deaths | Prevalence | CFR (95% CI) | Deaths | Prevalence | CFR (95% CI) |
| Invasive ventilation | 32 | 25.2% | 11.1% (8.0; 15.2) | 14 | 21.5% | 3.4% (1.3; 8.5) | 18 | 28.5% | 16.2% (11.4; 22.4) |
| Shock | 16 | 7% | 20.0% (12.7; 30.0) | 9 | 6.7% | 13.9% (6.1; 28.7) | 7 | 7.3% | 25.0% (14.6; 39.4) |
| Cardiac arrest or resuscitation | 19 | 5.2% | 31.7% (21.3; 44.2) | 9 | 5.2% | 7.1% (2; 22.6) | 10 | 5.3% | 53.1% (36.4; 69.1) |
| Use of systemic thrombolysis | 5 | 8.1% | 5.4% (2.3; 12.0) | 1 | 9.4% | 0% (0; 7.0) | 4 | 6.9% | 11.9% (5.2; 25) |
| Use of vasopressors | 21 | 13% | 14.1% (9.4; 20.6) | 8 | 10.7% | 3.4% (1.0; 11.7) | 13 | 15% | 20.9% (13.8; 30.3) |
| ECMO | 15 | 3.9% | 33.3% (21.4; 47.9) | 7 | 3.7% | 25.0% (11.2; 46.9) | 8 | 4.1% | 40.0% (23.4; 59.3) |
| Surgical thrombectomy | 0 | 0.6% | 0 | 0 | 0.6% | 0 | 0 | 0.7% | 0 |
| Any oft he above | 40 | 37.3% | 9.4% (7.0; 12.5) | 17 | 34.4% | 3.2% (1.5; 6.9) | 23 | 39.8% | 14.1% (10.3; 19.1) |
| None of the above | 4 | 62.7% | 0.6% (0.2; 1.4) | 3 | 65.6% | 0.3% (0; 1.6) | 1 | 60.2% | 0.8% (0.3; 2.4) |
|  |  |  |  |  |  |  |  |  |  |
| Catheter-directed treatment | 0 | 4.4% | 0 | 0 | 5.7% | 0 | 0 | 3.1% | 0 |
| Placement of an inferior vena cava filter | 0 | 0.9% | 0 | 0 | 0.9% | 0 | 0 | 0.8% | 0 |

ECMO: Extracorporeal membrane oxygenation.

# Table S21. Therapeutic procedures performed in patients with venous thromboembolism stratified by age and time period.

| **Characteristic** | **Overall,** N = 1,961 | **2004-2013** | | | **2014-2023** | | |
| --- | --- | --- | --- | --- | --- | --- | --- |
|  |  | **Overall**, N = 908 | **0 to 9 years old**, N = 225 | **10 to 19 years old**, N = 683 | **Overall**, N = 1,053 | **0 to 9 years old**, N = 454 | **10 to 19 years old**, N = 599 |
| Blood product transfusion, n (%) | 515 (26.3%) | 114 (12.6%) | 71 (31.6%) | 43 (6.3%) | 401 (38.1%) | 282 (62.1%) | 119 (19.9%) |
| Transfusion of erythrocytes | 473 (24.1%) | 102 (11.2%) | 64 (28.4%) | 38 (5.6%) | 371 (35.2%) | 262 (57.7%) | 109 (18.2%) |
| Transfusion of plasma | 275 (14.0%) | 46 (5.1%) | 38 (16.9%) | 8 (1.2%) | 229 (21.7%) | 176 (38.8%) | 53 (8.8%) |
| Transfusion of thrombocytes | 238 (12.1%) | 43 (4.7%) | 33 (14.7%) | 10 (1.5%) | 195 (18.5%) | 150 (33.0%) | 45 (7.5%) |
| Transfusion of coagulation factors | 183 (9.3%) | 17 (1.9%) | 15 (6.7%) | 2 (0.3%) | 166 (15.8%) | 127 (28.0%) | 39 (6.5%) |
| Invasive ventilation, n (%) | 367 (18.7%) | 125 (13.8%) | 97 (43.1%) | 28 (4.1%) | 242 (23.0%) | 194 (42.7%) | 48 (8.0%) |
| Central venous catheter, n/N (%) | 312 (15.9%) | 78 (8.6%) | 63 (28.0%) | 15 (2.2%) | 234 (22.2%) | 168 (37.0%) | 66 (11.0%) |
| Usage of a heart-lung machine (excl. ECMO), n (%) | 198 (10.1%) | 59 (6.5%) | 47 (20.9%) | 12 (1.8%) | 139 (13.2%) | 116 (25.6%) | 23 (3.8%) |
| Use of systemic thrombolysis, n (%) | 150 (7.6%) | 62 (6.8%) | 19 (8.4%) | 43 (6.3%) | 88 (8.4%) | 30 (6.6%) | 58 (9.7%) |
| Catheter-directed treatment, n (%) | 77 (3.9%) | 12 (1.3%) | 1 (0.4%) | 11 (1.6%) | 65 (6.2%) | 8 (1.8%) | 57 (9.5%) |
| Right-heart catheter, n (%) | 103 (5.3%) | 40 (4.4%) | 33 (14.7%) | 7 (1.0%) | 63 (6.0%) | 55 (12.1%) | 8 (1.3%) |
| Intervention at abdominal veins, n (%) | 66 (3.4%) | 8 (0.9%) | 0 (0.0%) | 8 (1.2%) | 58 (5.5%) | 5 (1.1%) | 53 (8.8%) |
| Extracorporeal membrane oxygenation, n (%) | 68 (3.5%) | 17 (1.9%) | 11 (4.9%) | 6 (0.9%) | 51 (4.8%) | 31 (6.8%) | 20 (3.3%) |
| Haemodialysis, n (%) | 64 (3.3%) | 15 (1.7%) | 9 (4.0%) | 6 (0.9%) | 49 (4.7%) | 26 (5.7%) | 23 (3.8%) |
| Intervention at pulmonary vessels, n (%) | 46 (2.3%) | 6 (0.7%) | 6 (2.7%) | 0 | 40 (3.8%) | 35 (7.7%) | 5 (0.8%) |
| Inferior vena cava filter placement, n (%) | 32 (1.6%) | 9 (1.0%) | 1 (0.4%) | 8 (1.2%) | 23 (2.2%) | 0 | 23 (3.8%) |
| Surgical thrombectomy, n (%) | 13 (0.7%) | 6 (0.7%) | 2 (0.9%) | 4 (0.6%) | 7 (0.7%) | 5 (1.1%) | 2 (0.3%) |

# Table S22. Therapeutic procedures performed in patients with pulmonary embolism and deep vein thrombosis stratified by age and time period.

| **Characteristic** | **Both diseases,** N = 1,961 | **Pulmonary embolism** | | | | | | | **Deep vein thrombosis (excluding cases with concomitant pulmonary embolism diagnosis)** | | | | | | |
| --- | --- | --- | --- | --- | --- | --- | --- | --- | --- | --- | --- | --- | --- | --- | --- |
|  |  | **Overall**, N = 815 | **2004-2013** | | | **2014-2023** | | | **Overall**, N = 1,146 | **2004-2013** | | | **2014-2023** | | |
|  |  |  | **Overall**, N = 410 | **0 to 9 years old**, N = 29 | **10 to 19 years old**, N = 381 | **Overall**, N = 405 | **0 to 9 years old**, N = 52 | **10 to 19 years old**, N = 353 |  | **Overall**, N = 498 | **0 to 9 years old**, N = 196 | **10 to 19 years old**, N = 302 | **Overall**, N = 648 | **0 to 9 years old**, N = 402 | **10 to 19 years old**, N = 246 |
| Blood product transfusion, n (%) | 515 (26.3%) | 150 (18.4%) | 38 (9.3%) | 11 (37.9%) | 27 (7.1%) | 112 (27.7%) | 37 (71.2%) | 75 (21.2%) | 365 (31.8%) | 76 (15.3%) | 60 (30.6%) | 16 (5.3%) | 289 (44.6%) | 245 (60.9%) | 44 (17.9%) |
| Transfusion of erythrocytes | 473 (24.1%) | 139 (17.1%) | 33 (8.0%) | 10 (34.5%) | 23 (6.0%) | 106 (26.2%) | 36 (69.2%) | 70 (19.8%) | 334 (29.1%) | 69 (13.9%) | 54 (27.6%) | 15 (5.0%) | 265 (40.9%) | 226 (56.2%) | 39 (15.9%) |
| Transfusion of plasma | 275 (14.0%) | 73 (9.0%) | 14 (3.4%) | 8 (27.6%) | 6 (1.6%) | 59 (14.6%) | 28 (53.8%) | 31 (8.8%) | 202 (17.6%) | 32 (6.4%) | 30 (15.3%) | 2 (0.7%) | 170 (26.2%) | 148 (36.8%) | 22 (8.9%) |
| Transfusion of thrombocytes | 238 (12.1%) | 64 (7.9%) | 14 (3.4%) | 7 (24.1%) | 7 (1.8%) | 50 (12.3%) | 23 (44.2%) | 27 (7.6%) | 174 (15.2%) | 29 (5.8%) | 26 (13.3%) | 3 (1.0%) | 145 (22.4%) | 127 (31.6%) | 18 (7.3%) |
| Transfusion of coagulation factors | 183 (9.3%) | 42 (5.2%) | 4 (1.0%) | 2 (6.9%) | 2 (0.5%) | 38 (9.4%) | 18 (34.6%) | 20 (5.7%) | 141 (12.3%) | 13 (2.6%) | 13 (6.6%) | 0 (0.0%) | 128 (19.8%) | 109 (27.1%) | 19 (7.7%) |
| Invasive ventilation, n (%) | 367 (18.7%) | 78 (9.6%) | 25 (6.1%) | 8 (27.6%) | 17 (4.5%) | 53 (13.1%) | 21 (40.4%) | 32 (9.1%) | 289 (25.2%) | 189 (29.2%) | 173 (43.0%) | 16 (6.5%) | 100 (20.1%) | 89 (45.4%) | 11 (3.6%) |
| Central venous catheter, n/N (%) | 312 (15.9%) | 82 (10.1%) | 19 (4.6%) | 9 (31.0%) | 10 (2.6%) | 63 (15.6%) | 18 (34.6%) | 45 (12.7%) | 230 (20.1%) | 59 (11.8%) | 54 (27.6%) | 5 (1.7%) | 171 (26.4%) | 150 (37.3%) | 21 (8.5%) |
| Usage of a heart-lung machine (excl. ECMO), n (%) | 198 (10.1%) | 54 (6.6%) | 16 (3.9%) | 9 (31.0%) | 7 (1.8%) | 38 (9.4%) | 21 (40.4%) | 17 (4.8%) | 144 (12.6%) | 43 (8.6%) | 38 (19.4%) | 5 (1.7%) | 101 (15.6%) | 95 (23.6%) | 6 (2.4%) |
| Use of systemic thrombolysis, n (%) | 150 (7.6%) | 57 (7.0%) | 26 (6.3%) | 5 (17.2%) | 21 (5.5%) | 31 (7.7%) | 6 (11.5%) | 25 (7.1%) | 93 (8.1%) | 36 (7.2%) | 14 (7.1%) | 22 (7.3%) | 57 (8.8%) | 24 (6.0%) | 33 (13.4%) |
| Catheter-directed treatment, n (%) | 77 (3.9%) | 27 (3.3%) | 4 (1.0%) | 0 | 4 (1.0%) | 23 (5.7%) | 5 (9.6%) | 18 (5.1%) | 50 (4.4%) | 8 (1.6%) | 1 (0.5%) | 7 (2.3%) | 42 (6.5%) | 3 (0.7%) | 39 (15.9%) |
| Right-heart catheter, n (%) | 103 (5.3%) | 23 (2.8%) | 9 (2.2%) | 7 (24.1%) | 2 (0.5%) | 14 (3.5%) | 11 (21.2%) | 3 (0.8%) | 80 (7.0%) | 31 (6.2%) | 26 (13.3%) | 5 (1.7%) | 49 (7.6%) | 44 (10.9%) | 5 (2.0%) |
| Intervention at abdominal veins, n (%) | 66 (3.4%) | 18 (2.2%) | 2 (0.5%) | 0 | 2 (0.5%) | 16 (4.0%) | 2 (3.8%) | 14 (4.0%) | 48 (4.2%) | 6 (1.2%) | 0 | 6 (2.0%) | 42 (6.5%) | 3 (0.7%) | 39 (15.9%) |
| Extracorporeal membrane oxygenation, n (%) | 68 (3.5%) | 23 (2.8%) | 6 (1.5%) | 2 (6.9%) | 4 (1.0%) | 17 (4.2%) | 6 (11.5%) | 11 (3.1%) | 45 (3.9%) | 34 (5.2%) | 25 (6.2%) | 9 (3.7%) | 11 (2.2%) | 9 (4.6%) | 2 (0.7%) |
| Haemodialysis, n (%) | 64 (3.3%) | 22 (2.7%) | 5 (1.2%) | 2 (6.9%) | 3 (0.8%) | 17 (4.2%) | 3 (5.8%) | 14 (4.0%) | 42 (3.7%) | 10 (2.0%) | 7 (3.6%) | 3 (1.0%) | 32 (4.9%) | 23 (5.7%) | 9 (3.7%) |
| Intervention at pulmonary vessels, n (%) | 46 (2.3%) | 14 (1.7%) | 4 (1.0%) | 4 (13.8%) | 0 | 10 (2.5%) | 7 (13.5%) | 3 (0.8%) | 32 (2.8%) | 2 (0.4%) | 2 (1.0%) | 0 | 30 (4.6%) | 28 (7.0%) | 2 (0.8%) |
| Inferior vena cava filter placement, n (%) | 32 (1.6%) | 22 (2.7%) | 5 (1.2%) | 0 | 5 (1.3%) | 17 (4.2%) | 0 | 17 (4.8%) | 10 (0.9%) | 4 (0.8%) | 1 (0.5%) | 3 (1.0%) | 6 (0.9%) | 0 | 6 (2.4%) |
| Surgical thrombectomy, n (%) | 13 (0.7%) | 6 (0.7%) | 3 (0.7%) | 0 (0.0%) | 3 (0.8%) | 3 (0.7%) | 2 (3.8%) | 1 (0.3%) | 7 (0.6%) | 4 (0.6%) | 3 (0.7%) | 1 (0.4%) | 3 (0.6%) | 2 (1.0%) | 1 (0.3%) |

# Table S23. Therapeutic procedures performed in patients with venous thromboembolism stratified by age and sex.

| **Characteristic** | **Overall,** N = 1,961 | **Female** | | | **Male** | | |
| --- | --- | --- | --- | --- | --- | --- | --- |
|  |  | **Overall**, N = 1,037 | **0 to 9 years old**, N = 276 | **10 to 19 years old**, N = 761 | **Overall**, N = 924 | **0 to 9 years old**, N = 403 | **10 to 19 years old**, N = 521 |
| Blood product transfusion, n (%) | 515 (26.3%) | 228 (22.0%) | 152 (55.1%) | 76 (10.0%) | 287 (31.1%) | 201 (49.9%) | 86 (16.5%) |
| Transfusion of erythrocytes | 473 (24.1%) | 212 (20.4%) | 145 (52.5%) | 67 (8.8%) | 261 (28.2%) | 181 (44.9%) | 80 (15.4%) |
| Transfusion of plasma | 275 (14.0%) | 112 (10.8%) | 86 (31.2%) | 26 (3.4%) | 163 (17.6%) | 128 (31.8%) | 35 (6.7%) |
| Transfusion of thrombocytes | 238 (12.1%) | 100 (9.6%) | 78 (28.3%) | 22 (2.9%) | 138 (14.9%) | 105 (26.1%) | 33 (6.3%) |
| Transfusion of coagulation factors | 183 (9.3%) | 73 (7.0%) | 55 (19.9%) | 18 (2.4%) | 110 (11.9%) | 87 (21.6%) | 23 (4.4%) |
| Invasive ventilation, n (%) | 367 (18.7%) | 158 (15.2%) | 120 (43.5%) | 38 (5.0%) | 209 (22.6%) | 171 (42.4%) | 38 (7.3%) |
| Central venous catheter, n/N (%) | 312 (15.9%) | 114 (11.0%) | 83 (30.1%) | 31 (4.1%) | 198 (21.4%) | 148 (36.7%) | 50 (9.6%) |
| Usage of a heart-lung machine (excl. ECMO), n (%) | 198 (10.1%) | 81 (7.8%) | 67 (24.3%) | 14 (1.8%) | 117 (12.7%) | 96 (23.8%) | 21 (4.0%) |
| Use of systemic thrombolysis, n (%) | 150 (7.6%) | 96 (9.3%) | 22 (8.0%) | 74 (9.7%) | 54 (5.8%) | 27 (6.7%) | 27 (5.2%) |
| Right-heart catheter, n (%) | 103 (5.3%) | 47 (4.5%) | 39 (14.1%) | 8 (1.1%) | 56 (6.1%) | 49 (12.2%) | 7 (1.3%) |
| Catheter-directed treatment, n (%) | 77 (3.9%) | 52 (5.0%) | 13 (4.7%) | 39 (5.1%) | 25 (2.7%) | 20 (5.0%) | 5 (1.0%) |
| Intervention at abdominal veins, n (%) | 66 (3.4%) | 45 (4.3%) | 2 (0.7%) | 43 (5.7%) | 21 (2.3%) | 3 (0.7%) | 18 (3.5%) |
| Extracorporeal membrane oxygenation, n (%) | 68 (3.5%) | 33 (3.2%) | 16 (5.8%) | 17 (2.2%) | 35 (3.8%) | 26 (6.5%) | 9 (1.7%) |
| Haemodialysis, n (%) | 46 (2.3%) | 18 (1.7%) | 14 (5.1%) | 4 (0.5%) | 28 (3.0%) | 27 (6.7%) | 1 (0.2%) |
| Intervention at pulmonary vessels, n (%) | 46 (2.3%) | 18 (1.7%) | 14 (5.1%) | 4 (0.5%) | 28 (3.0%) | 27 (6.7%) | 1 (0.2%) |
| Inferior vena cava filter placement, n (%) | 32 (1.6%) | 15 (1.4%) | 1 (0.4%) | 14 (1.8%) | 17 (1.8%) | 0 | 17 (3.3%) |
| Surgical thrombectomy, n (%) | 13 (0.7%) | 8 (0.8%) | 4 (1.4%) | 4 (0.5%) | 5 (0.5%) | 3 (0.7%) | 2 (0.4%) |

# Table S24. Therapeutic procedures performed in patients with pulmonary embolism stratified by age and sex.

| **Characteristic** | **Both diseases,** N = 1,961 | **Pulmonary embolism** | | | | | | | **Deep vein thrombosis (excluding cases with concomitant pulmonary embolism diagnosis)** | | | | | | |
| --- | --- | --- | --- | --- | --- | --- | --- | --- | --- | --- | --- | --- | --- | --- | --- |
|  |  | **Overall**, N = 815 | **Female** | | | **Male** | | | **Overall**, N = 1,146 | **Female** | | | **Male** | | |
|  |  |  | **Overall**, N = 497 | **0 to 9 years old**, N = 35 | **10 to 19 years old**, N = 462 | **Overall**, N = 318 | **0 to 9 years old**, N = 46 | **10 to 19 years old**, N = 272 |  | **Overall**, N = 540 | **0 to 9 years old**, N = 241 | **10 to 19 years old**, N = 299 | **Overall**, N = 606 | **0 to 9 years old**, N = 357 | **10 to 19 years old**, N = 249 |
| Blood product transfusion, n (%) | 515 (26.3%) | 150 (18.4%) | 67 (13.5%) | 22 (62.9%) | 45 (9.7%) | 83 (26.1%) | 26 (56.5%) | 57 (21.0%) | 365 (31.8%) | 161 (29.8%) | 130 (53.9%) | 31 (10.4%) | 204 (33.7%) | 175 (49.0%) | 29 (11.6%) |
| Transfusion of erythrocytes | 473 (24.1%) | 139 (17.1%) | 59 (11.9%) | 20 (57.1%) | 39 (8.4%) | 80 (25.2%) | 26 (56.5%) | 54 (19.9%) | 334 (29.1%) | 153 (28.3%) | 125 (51.9%) | 28 (9.4%) | 181 (29.9%) | 155 (43.4%) | 26 (10.4%) |
| Transfusion of plasma | 275 (14.0%) | 73 (9.0%) | 33 (6.6%) | 17 (48.6%) | 16 (3.5%) | 40 (12.6%) | 19 (41.3%) | 21 (7.7%) | 202 (17.6%) | 79 (14.6%) | 69 (28.6%) | 10 (3.3%) | 123 (20.3%) | 109 (30.5%) | 14 (5.6%) |
| Transfusion of thrombocytes | 238 (12.1%) | 64 (7.9%) | 30 (6.0%) | 17 (48.6%) | 13 (2.8%) | 34 (10.7%) | 13 (28.3%) | 21 (7.7%) | 174 (15.2%) | 70 (13.0%) | 61 (25.3%) | 9 (3.0%) | 104 (17.2%) | 92 (25.8%) | 12 (4.8%) |
| Transfusion of coagulation factors | 183 (9.3%) | 42 (5.2%) | 20 (4.0%) | 11 (31.4%) | 9 (1.9%) | 22 (6.9%) | 9 (19.6%) | 13 (4.8%) | 141 (12.3%) | 53 (9.8%) | 44 (18.3%) | 9 (3.0%) | 88 (14.5%) | 78 (21.8%) | 10 (4.0%) |
| Invasive ventilation, n (%) | 367 (18.7%) | 78 (9.6%) | 42 (8.5%) | 17 (48.6%) | 25 (5.4%) | 36 (11.3%) | 12 (26.1%) | 24 (8.8%) | 289 (25.2%) | 116 (21.5%) | 103 (42.7%) | 13 (4.3%) | 173 (28.5%) | 159 (44.5%) | 14 (5.6%) |
| Central venous catheter, n/N (%) | 312 (15.9%) | 82 (10.1%) | 32 (6.4%) | 9 (25.7%) | 23 (5.0%) | 50 (15.7%) | 18 (39.1%) | 32 (11.8%) | 230 (20.1%) | 82 (15.2%) | 74 (30.7%) | 8 (2.7%) | 148 (24.4%) | 130 (36.4%) | 18 (7.2%) |
| Usage of a heart-lung machine (excl. ECMO), n (%) | 198 (10.1%) | 54 (6.6%) | 25 (5.0%) | 15 (42.9%) | 10 (2.2%) | 29 (9.1%) | 15 (32.6%) | 14 (5.1%) | 144 (12.6%) | 56 (10.4%) | 52 (21.6%) | 4 (1.3%) | 88 (14.5%) | 81 (22.7%) | 7 (2.8%) |
| Use of systemic thrombolysis, n (%) | 150 (7.6%) | 57 (7.0%) | 45 (9.1%) | 7 (20.0%) | 38 (8.2%) | 12 (3.8%) | 4 (8.7%) | 8 (2.9%) | 93 (8.1%) | 51 (9.4%) | 15 (6.2%) | 36 (12.0%) | 42 (6.9%) | 23 (6.4%) | 19 (7.6%) |
| Catheter-directed treatment, n (%) | 77 (3.9%) | 27 (3.3%) | 21 (4.2%) | 3 (8.6%) | 18 (3.9%) | 6 (1.9%) | 2 (4.3%) | 4 (1.5%) | 50 (4.4%) | 31 (5.7%) | 1 (0.4%) | 30 (10.0%) | 19 (3.1%) | 3 (0.8%) | 16 (6.4%) |
| Right-heart catheter, n (%) | 103 (5.3%) | 23 (2.8%) | 11 (2.2%) | 8 (22.9%) | 3 (0.6%) | 12 (3.8%) | 10 (21.7%) | 2 (0.7%) | 80 (7.0%) | 36 (6.7%) | 31 (12.9%) | 5 (1.7%) | 44 (7.3%) | 39 (10.9%) | 5 (2.0%) |
| Intervention at abdominal veins, n (%) | 66 (3.4%) | 18 (2.2%) | 15 (3.0%) | 1 (2.9%) | 14 (3.0%) | 3 (0.9%) | 1 (2.2%) | 2 (0.7%) | 48 (4.2%) | 30 (5.6%) | 1 (0.4%) | 29 (9.7%) | 18 (3.0%) | 2 (0.6%) | 16 (6.4%) |
| Extracorporeal membrane oxygenation, n (%) | 68 (3.5%) | 23 (2.8%) | 13 (2.6%) | 2 (5.7%) | 11 (2.4%) | 10 (3.1%) | 6 (13.0%) | 4 (1.5%) | 45 (3.9%) | 20 (3.7%) | 14 (5.8%) | 6 (2.0%) | 25 (4.1%) | 20 (5.6%) | 5 (2.0%) |
| Haemodialysis, n (%) | 64 (3.3%) | 22 (2.7%) | 12 (2.4%) | 2 (5.7%) | 10 (2.2%) | 10 (3.1%) | 3 (6.5%) | 7 (2.6%) | 42 (3.7%) | 21 (3.9%) | 15 (6.2%) | 6 (2.0%) | 21 (3.5%) | 15 (4.2%) | 6 (2.4%) |
| Intervention at pulmonary vessels, n (%) | 46 (2.3%) | 14 (1.7%) | 7 (1.4%) | 4 (11.4%) | 3 (0.6%) | 7 (2.2%) | 7 (15.2%) | 0 | 32 (2.8%) | 11 (2.0%) | 10 (4.1%) | 1 (0.3%) | 21 (3.5%) | 20 (5.6%) | 1 (0.4%) |
| Inferior vena cava filter placement, n (%) | 32 (1.6%) | 22 (2.7%) | 10 (2.0%) | 0 | 10 (2.2%) | 12 (3.8%) | 0 | 12 (4.4%) | 10 (0.9%) | 5 (0.9%) | 1 (0.4%) | 4 (1.3%) | 5 (0.8%) | 0 | 5 (2.0%) |
| Surgical thrombectomy, n (%) | 13 (0.7%) | 6 (0.7%) | 5 (1%) | 2 (5.7%) | 3 (0.6%) | 1 (0.3%) | 0 | 1 (0.4%) | 7 (0.6%) | 3 (0.6%) | 2 (0.8%) | 1 (0.3%) | 4 (0.7%) | 3 (0.8%) | 1 (0.4%) |

# Table S25. Comorbidities in patients with venous thromboembolism stratified by age and time period.

| **Characteristic** | **Overall,** N = 1,961 | **2004-2013** | | | **2014-2023** | | |
| --- | --- | --- | --- | --- | --- | --- | --- |
|  |  | **Overall**, N = 908 | **0 to 9 years old**, N = 225 | **10 to 19 years old**, N = 683 | **Overall**, N = 1,053 | **0 to 9 years old**, N = 454 | **10 to 19 years old**, N = 599 |
| Injury or trauma, n (%) | 762 (38.9%) | 239 (26.3%) | 102 (45.3%) | 137 (20.1%) | 523 (49.7%) | 297 (65.4%) | 226 (37.7%) |
| Respiratory diseases, n (%) | 652 (33.2%) | 218 (24.0%) | 96 (42.7%) | 122 (17.9%) | 434 (41.2%) | 221 (48.7%) | 213 (35.6%) |
| Pneumonia | 185 (9.4%) | 68 (7.5%) | 33 (14.7%) | 35 (5.1%) | 117 (11.1%) | 60 (13.2%) | 57 (9.5%) |
| Respiratory failure | 172 (8.8%) | 48 (5.3%) | 31 (13.8%) | 17 (2.5%) | 124 (11.8%) | 73 (16.1%) | 51 (8.5%) |
| Asthma | 22 (1.1%) | 18 (2.0%) | 0 (0.0%) | 18 (2.6%) | 4 (0.4%) | 0 (0.0%) | 4 (0.7%) |
| Acute bronchiolitis | 20 (1.0%) | 5 (0.6%) | 5 (2.2%) | 0 | 15 (1.4%) | 14 (3.1%) | 1 (0.2%) |
| Acute bronchitis | 18 (0.9%) | 6 (0.7%) | 6 (2.7%) | 0 | 12 (1.1%) | 10 (2.2%) | 2 (0.3%) |
| Infectious diseases, n (%) | 535 (27.3%) | 156 (17.2%) | 81 (36.0%) | 75 (11.0%) | 379 (36.0%) | 231 (50.9%) | 148 (24.7%) |
| Sepsis | 161 (8.2%) | 50 (5.5%) | 32 (14.2%) | 18 (2.6%) | 111 (10.5%) | 67 (14.8%) | 44 (7.3%) |
| Cardiovascular diseases, n (%) | 463 (23.6%) | 135 (14.9%) | 88 (39.1%) | 47 (6.9%) | 328 (31.1%) | 221 (48.7%) | 107 (17.9%) |
| Diseases of arteries, arterioles and capillaries | 119 (6.1%) | 22 (2.4%) | 17 (7.6%) | 5 (0.7%) | 97 (9.2%) | 75 (16.5%) | 22 (3.7%) |
| Pulmonary hearth disease | 103 (5.3%) | 34 (3.7%) | 23 (10.2%) | 11 (1.6%) | 69 (6.6%) | 61 (13.4%) | 8 (1.3%) |
| Arterial hypertension | 102 (5.2%) | 27 (3.0%) | 23 (10.2%) | 4 (0.6%) | 75 (7.1%) | 54 (11.9%) | 21 (3.5%) |
| Heart failure | 90 (4.6%) | 25 (2.8%) | 17 (7.6%) | 8 (1.2%) | 65 (6.2%) | 41 (9.0%) | 24 (4.0%) |
| Cerebral infraction | 47 (2.4%) | 8 (0.9%) | 6 (2.7%) | 2 (0.3%) | 39 (3.7%) | 16 (3.5%) | 23 (3.8%) |
| Acute and subacute endocarditis | 24 (1.2%) | 5 (0.6%) | 3 (1.3%) | 2 (0.3%) | 19 (1.8%) | 4 (0.9%) | 15 (2.5%) |
| Cardiomyopathy | 24 (1.2%) | 7 (0.8%) | 3 (1.3%) | 4 (0.6%) | 17 (1.6%) | 10 (2.2%) | 7 (1.2%) |
| Ischemic heart disease | 20 (1.0%) | 6 (0.7%) | 3 (1.3%) | 3 (0.4%) | 14 (1.3%) | 10 (2.2%) | 4 (0.7%) |
| Acute myocarditis | 4 (0.2%) | 2 (0.2%) | 1 (0.4%) | 1 (0.1%) | 2 (0.2%) | 0 | 2 (0.3%) |
| Chronic rheumatic heart disease | 1 (0.1%) | 1 (0.1%) | 1 (0.4%) | 0 | 0 | 0 | 0 |
| Metabolic disorders, n (%) | 374 (19.1%) | 100 (11.0%) | 64 (28.4%) | 36 (5.3%) | 274 (26.0%) | 185 (40.7%) | 89 (14.9%) |
| Haemorrhage, n (%) | 313 (16.0%) | 84 (9.3%) | 36 (16.0%) | 48 (7.0%) | 229 (21.7%) | 130 (28.6%) | 99 (16.5%) |
| Congenital heart disease, n (%) | 313 (16.0%) | 96 (10.6%) | 81 (36.0%) | 15 (2.2%) | 217 (20.6%) | 197 (43.4%) | 20 (3.3%) |
| Coagulation disturbances or thrombophilia, n (%) | 278 (14.2%) | 85 (9.4%) | 18 (8.0%) | 67 (9.8%) | 193 (18.3%) | 110 (24.2%) | 83 (13.9%) |
| Secondary thrombophilia | 97 (4.9%) | 5 (0.6%) | 4 (1.8%) | 1 (0.1%) | 92 (8.7%) | 63 (13.9%) | 29 (4.8%) |
| Hereditary deficiency of clotting factors | 40 (2.0%) | 9 (1.0%) | 1 (0.4%) | 8 (1.2%) | 31 (2.9%) | 12 (2.6%) | 19 (3.2%) |
| Primary thrombophilia | 38 (1.9%) | 27 (3.0%) | 1 (0.4%) | 26 (3.8%) | 11 (1.0%) | 2 (0.4%) | 9 (1.5%) |
| Cancer, n (%) | 211 (10.8%) | 78 (8.6%) | 20 (8.9%) | 58 (8.5%) | 133 (12.6%) | 52 (11.5%) | 81 (13.5%) |
| Venous thromboembolism not including PE or DVT, n (%) | 183 (9.3%) | 47 (5.2%) | 18 (8.0%) | 29 (4.2%) | 136 (12.9%) | 101 (22.2%) | 35 (5.8%) |
| Renal failure, n (%) | 160 (8.2%) | 38 (4.2%) | 22 (9.8%) | 16 (2.3%) | 122 (11.6%) | 77 (17.0%) | 45 (7.5%) |
| Malnutrition, n (%) | 56 (2.9%) | 7 (0.8%) | 4 (1.8%) | 3 (0.4%) | 49 (4.7%) | 18 (4.0%) | 31 (5.2%) |
| Venous thrombosis of the deep veins of the upper extremities, n (%) | 48 (2.4%) | 3 (0.3%) | 2 (0.9%) | 1 (0.1%) | 45 (4.3%) | 27 (5.9%) | 18 (3.0%) |
| Obesity, n (%) | 42 (2.1%) | 31 (3.4%) | 0 | 31 (4.5%) | 11 (1.0%) | 0 | 11 (1.8%) |
| Osteomyelitis, n (%) | 24 (1.2%) | 10 (1.1%) | 2 (0.9%) | 8 (1.2%) | 14 (1.3%) | 7 (1.5%) | 7 (1.2%) |
| Intracerebral haemorrhage, n (%) | 28 (1.4%) | 8 (0.9%) | 5 (2.2%) | 3 (0.4%) | 20 (1.9%) | 15 (3.3%) | 5 (0.8%) |
| Peritonitis, n (%) | 20 (1.0%) | 2 (0.2%) | 1 (0.4%) | 1 (0.1%) | 18 (1.7%) | 14 (3.1%) | 4 (0.7%) |
| Glomerular diseases, n (%) | 17 (0.9%) | 9 (1.0%) | 3 (1.3%) | 6 (0.9%) | 8 (0.8%) | 3 (0.7%) | 5 (0.8%) |
| Nephrotic syndrome | 5 (0.3%) | 3 (0.3%) | 1 (0.4%) | 2 (0.3%) | 2 (0.2%) | 2 (0.4%) | 0 |
| Corona virus disease 2019, n (%) | 16 (0.8%) | n. a. | n. a. | n. a. | 16 (1.5%) | 3 (0.7%) | 13 (2.2%) |
| Diabetes mellitus, n (%) | 8 (0.4%) | 3 (0.3%) | 1 (0.4%) | 2 (0.3%) | 5 (0.5%) | 2 (0.4%) | 3 (0.5%) |
| Hyposplenism, n (%) | 3 (0.2%) | 1 (0.1%) | 0 | 1 (0.1%) | 2 (0.2%) | 1 (0.2%) | 1 (0.2%) |

n. a.: not applicable

# Table S26. Comorbidities in patients with pulmonary embolism and deep vein thrombosis stratified by age and time period.

| **Characteristic** | **Both diseases,** N = 1,961 | **Pulmonary embolism** | | | | | | | **Deep vein thrombosis (excluding cases with concomitant pulmonary embolism diagnosis)** | | | | | | |
| --- | --- | --- | --- | --- | --- | --- | --- | --- | --- | --- | --- | --- | --- | --- | --- |
|  |  | **Overall**, N = 815 | **2004-2013** | | | **2014-2023** | | | **Overall**, N = 1,146 | **2004-2013** | | | **2014-2023** | | |
|  |  |  | **Overall**, N = 410 | **0 to 9 years old**, N = 29 | **10 to 19 years old**, N = 381 | **Overall**, N = 405 | **0 to 9 years old**, N = 52 | **10 to 19 years old**, N = 353 |  | **Overall**, N = 498 | **0 to 9 years old**, N = 196 | **10 to 19 years old**, N = 302 | **Overall**, N = 648 | **0 to 9 years old**, N = 402 | **10 to 19 years old**, N = 246 |
| Injury or trauma, n (%) | 762 (38.9%) | 242 (29.7%) | 80 (19.5%) | 14 (48.3%) | 66 (17.3%) | 162 (40.0%) | 33 (63.5%) | 129 (36.5%) | 520 (45.4%) | 159 (31.9%) | 88 (44.9%) | 71 (23.5%) | 361 (55.7%) | 264 (65.7%) | 97 (39.4%) |
| Respiratory diseases, n (%) | 652 (33.2%) | 314 (38.5%) | 116 (28.3%) | 15 (51.7%) | 101 (26.5%) | 198 (48.9%) | 31 (59.6%) | 167 (47.3%) | 338 (29.5%) | 102 (20.5%) | 81 (41.3%) | 21 (7.0%) | 236 (36.4%) | 190 (47.3%) | 46 (18.7%) |
| Pneumonia | 185 (9.4%) | 91 (11.2%) | 34 (8.3%) | 5 (17.2%) | 29 (7.6%) | 57 (14.1%) | 14 (26.9%) | 43 (12.2%) | 94 (8.2%) | 34 (6.8%) | 28 (14.3%) | 6 (2.0%) | 60 (9.3%) | 46 (11.4%) | 14 (5.7%) |
| Respiratory failure | 172 (8.8%) | 69 (8.5%) | 18 (4.4%) | 5 (17.2%) | 13 (3.4%) | 51 (12.6%) | 10 (19.2%) | 41 (11.6%) | 103 (9.0%) | 30 (6.0%) | 26 (13.3%) | 4 (1.3%) | 73 (11.3%) | 63 (15.7%) | 10 (4.1%) |
| Asthma | 22 (1.1%) | 16 (2.0%) | 14 (3.4%) | 0 (0.0%) | 14 (3.7%) | 2 (0.5%) | 0 (0.0%) | 2 (0.6%) | 6 (0.5%) | 4 (0.8%) | 0 (0.0%) | 4 (1.3%) | 2 (0.3%) | 0 (0.0%) | 2 (0.8%) |
| Acute bronchiolitis | 20 (1.0%) | 5 (0.6%) | 1 (0.2%) | 1 (3.4%) | 0 (0.0%) | 4 (1.0%) | 3 (5.8%) | 1 (0.3%) | 15 (1.3%) | 4 (0.8%) | 4 (2.0%) | 0 (0.0%) | 11 (1.7%) | 11 (2.7%) | 0 (0.0%) |
| Acute bronchitis | 18 (0.9%) | 4 (0.5%) | 1 (0.2%) | 1 (3.4%) | 0 (0.0%) | 3 (0.7%) | 1 (1.9%) | 2 (0.6%) | 14 (1.2%) | 5 (1.0%) | 5 (2.6%) | 0 (0.0%) | 9 (1.4%) | 9 (2.2%) | 0 (0.0%) |
| Infectious diseases, n (%) | 535 (27.3%) | 176 (21.6%) | 54 (13.2%) | 14 (48.3%) | 40 (10.5%) | 122 (30.1%) | 27 (51.9%) | 95 (26.9%) | 359 (31.3%) | 102 (20.5%) | 67 (34.2%) | 35 (11.6%) | 257 (39.7%) | 204 (50.7%) | 53 (21.5%) |
| Sepsis | 161 (8.2%) | 65 (8.0%) | 20 (4.9%) | 9 (31.0%) | 11 (2.9%) | 45 (11.1%) | 12 (23.1%) | 33 (9.3%) | 96 (8.4%) | 30 (6.0%) | 23 (11.7%) | 7 (2.3%) | 66 (10.2%) | 55 (13.7%) | 11 (4.5%) |
| Cardiovascular diseases, n (%) | 463 (23.6%) | 137 (16.8%) | 40 (9.8%) | 11 (37.9%) | 29 (7.6%) | 97 (24.0%) | 26 (50.0%) | 71 (20.1%) | 326 (28.4%) | 95 (19.1%) | 77 (39.3%) | 18 (6.0%) | 231 (35.6%) | 195 (48.5%) | 36 (14.6%) |
| Diseases of arteries, arterioles and capillaries | 119 (6.1%) | 30 (3.7%) | 7 (1.7%) | 4 (13.8%) | 3 (0.8%) | 23 (5.7%) | 9 (17.3%) | 14 (4.0%) | 89 (7.8%) | 15 (3.0%) | 13 (6.6%) | 2 (0.7%) | 74 (11.4%) | 66 (16.4%) | 8 (3.3%) |
| Pulmonary hearth disease | 103 (5.3%) | 28 (3.4%) | 13 (3.2%) | 6 (20.7%) | 7 (1.8%) | 15 (3.7%) | 10 (19.2%) | 5 (1.4%) | 75 (6.5%) | 21 (4.2%) | 17 (8.7%) | 4 (1.3%) | 54 (8.3%) | 51 (12.7%) | 3 (1.2%) |
| Arterial hypertension | 102 (5.2%) | 21 (2.6%) | 3 (0.7%) | 2 (6.9%) | 1 (0.3%) | 18 (4.4%) | 8 (15.4%) | 10 (2.8%) | 81 (7.1%) | 24 (4.8%) | 21 (10.7%) | 3 (1.0%) | 57 (8.8%) | 46 (11.4%) | 11 (4.5%) |
| Heart failure | 90 (4.6%) | 28 (3.4%) | 8 (2.0%) | 2 (6.9%) | 6 (1.6%) | 20 (4.9%) | 3 (5.8%) | 17 (4.8%) | 62 (5.4%) | 17 (3.4%) | 15 (7.7%) | 2 (0.7%) | 45 (6.9%) | 38 (9.5%) | 7 (2.8%) |
| Cerebral infraction | 47 (2.4%) | 13 (1.6%) | 1 (0.2%) | 1 (3.4%) | 0 | 12 (3.0%) | 2 (3.8%) | 10 (2.8%) | 34 (3.0%) | 7 (1.4%) | 5 (2.6%) | 2 (0.7%) | 27 (4.2%) | 14 (3.5%) | 13 (5.3%) |
| Acute and subacute endocarditis | 24 (1.2%) | 13 (1.6%) | 3 (0.7%) | 1 (3.4%) | 2 (0.5%) | 10 (2.5%) | 0 | 10 (2.8%) | 11 (1.0%) | 2 (0.4%) | 2 (1.0%) | 0 | 9 (1.4%) | 4 (1.0%) | 5 (2.0%) |
| Cardiomyopathy | 24 (1.2%) | 9 (1.1%) | 5 (1.2%) | 1 (3.4%) | 4 (1.0%) | 4 (1.0%) | 2 (3.8%) | 2 (0.6%) | 15 (1.3%) | 2 (0.4%) | 2 (1.0%) | 0 | 13 (2.0%) | 8 (2.0%) | 5 (2.0%) |
| Ischemic heart disease | 20 (1.0%) | 6 (0.7%) | 2 (0.5%) | 0 | 2 (0.5%) | 4 (1.0%) | 3 (5.8%) | 1 (0.3%) | 14 (1.2%) | 4 (0.8%) | 3 (1.5%) | 1 (0.3%) | 10 (1.5%) | 7 (1.7%) | 3 (1.2%) |
| Acute myocarditis | 4 (0.2%) | 1 (0.1%) | 1 (0.2%) | 0 | 1 (0.3%) | 0 | 0 | 0 | 3 (0.3%) | 1 (0.2%) | 1 (0.5%) | 0 | 2 (0.3%) | 0 | 2 (0.8%) |
| Chronic rheumatic heart disease | 1 (0.1%) | 0 | 0 | 0 | 0 | 0 | 0 | 0 | 1 (0.1%) | 1 (0.2%) | 1 (0.5%) | 0 | 0 | 0 | 0 |
| Metabolic disorders, n (%) | 374 (19.1%) | 115 (14.1%) | 33 (8.0%) | 11 (37.9%) | 22 (5.8%) | 82 (20.2%) | 27 (51.9%) | 55 (15.6%) | 259 (22.6%) | 67 (13.5%) | 53 (27.0%) | 14 (4.6%) | 192 (29.6%) | 158 (39.3%) | 34 (13.8%) |
| Haemorrhage, n (%) | 313 (16.0%) | 118 (14.5%) | 32 (7.8%) | 3 (10.3%) | 29 (7.6%) | 86 (21.2%) | 22 (42.3%) | 64 (18.1%) | 195 (17.0%) | 52 (10.4%) | 33 (16.8%) | 19 (6.3%) | 143 (22.1%) | 108 (26.9%) | 35 (14.2%) |
| Congenital heart disease, n (%) | 313 (16.0%) | 52 (6.4%) | 16 (3.9%) | 10 (34.5%) | 6 (1.6%) | 36 (8.9%) | 22 (42.3%) | 14 (4.0%) | 261 (22.8%) | 80 (16.1%) | 71 (36.2%) | 9 (3.0%) | 181 (27.9%) | 175 (43.5%) | 6 (2.4%) |
| Coagulation disturbances or thrombophilia, n (%) | 278 (14.2%) | 102 (12.5%) | 36 (8.8%) | 5 (17.2%) | 31 (8.1%) | 66 (16.3%) | 13 (25.0%) | 53 (15.0%) | 176 (15.4%) | 49 (9.8%) | 13 (6.6%) | 36 (11.9%) | 127 (19.6%) | 97 (24.1%) | 30 (12.2%) |
| Secondary thrombophilia | 97 (4.9%) | 30 (3.7%) | 1 (0.2%) | 1 (3.4%) | 0 (0.0%) | 29 (7.2%) | 7 (13.5%) | 22 (6.2%) | 67 (5.8%) | 4 (0.8%) | 3 (1.5%) | 1 (0.3%) | 63 (9.7%) | 56 (13.9%) | 7 (2.8%) |
| Hereditary deficiency of clotting factors | 40 (2.0%) | 13 (1.6%) | 4 (1.0%) | 1 (3.4%) | 3 (0.8%) | 9 (2.2%) | 1 (1.9%) | 8 (2.3%) | 27 (2.4%) | 5 (1.0%) | 0 | 5 (1.7%) | 22 (3.4%) | 11 (2.7%) | 11 (4.5%) |
| Primary thrombophilia | 38 (1.9%) | 18 (2.2%) | 10 (2.4%) | 0 | 10 (2.6%) | 8 (2.0%) | 0 | 8 (2.3%) | 20 (1.7%) | 17 (3.4%) | 1 (0.5%) | 16 (5.3%) | 3 (0.5%) | 2 (0.5%) | 1 (0.4%) |
| Cancer, n (%) | 211 (10.8%) | 70 (8.6%) | 25 (6.1%) | 1 (3.4%) | 24 (6.3%) | 45 (11.1%) | 13 (25.0%) | 32 (9.1%) | 141 (12.3%) | 53 (10.6%) | 19 (9.7%) | 34 (11.3%) | 88 (13.6%) | 39 (9.7%) | 49 (19.9%) |
| Venous thromboembolism not including PE or DVT, n (%) | 183 (9.3%) | 49 (6.0%) | 17 (4.1%) | 1 (3.4%) | 16 (4.2%) | 32 (7.9%) | 12 (23.1%) | 20 (5.7%) | 134 (11.7%) | 30 (6.0%) | 17 (8.7%) | 13 (4.3%) | 104 (16.0%) | 89 (22.1%) | 15 (6.1%) |
| Renal failure, n (%) | 160 (8.2%) | 55 (6.7%) | 14 (3.4%) | 3 (10.3%) | 11 (2.9%) | 41 (10.1%) | 11 (21.2%) | 30 (8.5%) | 105 (9.2%) | 24 (4.8%) | 19 (9.7%) | 5 (1.7%) | 81 (12.5%) | 66 (16.4%) | 15 (6.1%) |
| Malnutrition, n (%) | 56 (2.9%) | 26 (3.2%) | 2 (0.5%) | 1 (3.4%) | 1 (0.3%) | 24 (5.9%) | 5 (9.6%) | 19 (5.4%) | 30 (2.6%) | 5 (1.0%) | 3 (1.5%) | 2 (0.7%) | 25 (3.9%) | 13 (3.2%) | 12 (4.9%) |
| Venous thrombosis of the deep veins of the upper extremities, n (%) | 48 (2.4%) | 12 (1.5%) | 1 (0.2%) | 0 | 1 (0.3%) | 11 (2.7%) | 4 (7.7%) | 7 (2.0%) | 36 (3.1%) | 2 (0.4%) | 2 (1.0%) | 0 | 34 (5.2%) | 23 (5.7%) | 11 (4.5%) |
| Obesity, n (%) | 42 (2.1%) | 26 (3.2%) | 20 (4.9%) | 0 | 20 (5.2%) | 6 (1.5%) | 0 | 6 (1.7%) | 16 (1.4%) | 11 (2.2%) | 0 | 11 (3.6%) | 5 (0.8%) | 0 | 5 (2.0%) |
| Osteomyelitis, n (%) | 24 (1.2%) | 9 (1.1%) | 3 (0.7%) | 1 (3.4%) | 2 (0.5%) | 6 (1.5%) | 1 (1.9%) | 5 (1.4%) | 15 (1.3%) | 7 (1.4%) | 1 (0.5%) | 6 (2.0%) | 8 (1.2%) | 6 (1.5%) | 2 (0.8%) |
| Intracerebral haemorrhage, n (%) | 28 (1.4%) | 10 (1.2%) | 3 (0.7%) | 0 | 3 (0.8%) | 7 (1.7%) | 4 (7.7%) | 3 (0.8%) | 18 (1.6%) | 5 (1.0%) | 5 (2.6%) | 0 | 13 (2.0%) | 11 (2.7%) | 2 (0.8%) |
| Peritonitis, n (%) | 20 (1.0%) | 6 (0.7%) | 0 | 0 | 0 | 6 (1.5%) | 2 (3.8%) | 4 (1.1%) | 14 (1.2%) | 2 (0.4%) | 1 (0.5%) | 1 (0.3%) | 12 (1.9%) | 12 (3.0%) | 0 |
| Glomerular diseases, n (%) | 17 (0.9%) | 8 (1.0%) | 6 (1.5%) | 2 (6.9%) | 4 (1.0%) | 2 (0.5%) | 0 | 2 (0.6%) | 9 (0.8%) | 3 (0.6%) | 1 (0.5%) | 2 (0.7%) | 6 (0.9%) | 3 (0.7%) | 3 (1.2%) |
| Nephrotic syndrome | 5 (0.3%) | 3 (0.4%) | 3 (0.7%) | 1 (3.4%) | 2 (0.5%) | 0 | 0 | 0 | 2 (0.2%) | 0 | 0 | 0 | 2 (0.3%) | 2 (0.5%) | 0 |
| Corona virus disease 2019, n (%) | 16 (0.8%) | 9 (1.1%) | n. a. | n. a. | n. a. | 9 (2.2%) | 1 (1.9%) | 8 (2.3%) | 7 (0.6%) | n. a. | n. a. | n. a. | 7 (1.1%) | 2 (0.5%) | 5 (2.0%) |
| Diabetes mellitus, n (%) | 8 (0.4%) | 4 (0.5%) | 2 (0.5%) | 0 | 2 (0.5%) | 2 (0.5%) | 0 | 2 (0.6%) | 4 (0.3%) | 1 (0.2%) | 1 (0.5%) | 0 | 3 (0.5%) | 2 (0.5%) | 1 (0.4%) |
| Hyposplenism, n (%) | 3 (0.2%) | 2 (0.2%) | 1 (0.2%) | `0 | 1 (0.3%) | 1 (0.2%) | 0 | 1 (0.3%) | 1 (0.1%) | 0 | 0 | 0 | 1 (0.2%) | 1 (0.2%) | 0 |

n. a.: not applicable

# Table S27. Comorbidities in patients with venous thromboembolism stratified by age and sex.

| **Characteristic** | **Overall,** N = 1,961 | **Female** | | | **Male** | | |
| --- | --- | --- | --- | --- | --- | --- | --- |
|  |  | **Overall**, N = 1,037 | **0 to 9 years old**, N = 276 | **10 to 19 years old**, N = 761 | **Overall**, N = 924 | **0 to 9 years old**, N = 403 | **10 to 19 years old**, N = 521 |
| Injury or trauma, n (%) | 762 (38.9%) | 335 (32.3%) | 174 (63.0%) | 161 (21.2%) | 427 (46.2%) | 225 (55.8%) | 202 (38.8%) |
| Respiratory diseases, n (%) | 652 (33.2%) | 315 (30.4%) | 138 (50.0%) | 177 (23.3%) | 337 (36.5%) | 179 (44.4%) | 158 (30.3%) |
| Pneumonia | 185 (9.4%) | 86 (8.3%) | 41 (14.9%) | 45 (5.9%) | 99 (10.7%) | 52 (12.9%) | 47 (9.0%) |
| Respiratory failure | 172 (8.8%) | 74 (7.1%) | 47 (17.0%) | 27 (3.5%) | 98 (10.6%) | 57 (14.1%) | 41 (7.9%) |
| Asthma | 22 (1.1%) | 15 (1.4%) | 0 (0.0%) | 15 (2.0%) | 7 (0.8%) | 0 (0.0%) | 7 (1.3%) |
| Acute bronchiolitis | 20 (1.0%) | 9 (0.9%) | 9 (3.3%) | 0 (0.0%) | 11 (1.2%) | 10 (2.5%) | 1 (0.2%) |
| Acute bronchitis | 18 (0.9%) | 7 (0.7%) | 6 (2.2%) | 1 (0.1%) | 11 (1.2%) | 10 (2.5%) | 1 (0.2%) |
| Infectious diseases, n (%) | 535 (27.3%) | 237 (22.9%) | 135 (48.9%) | 102 (13.4%) | 298 (32.3%) | 177 (43.9%) | 121 (23.2%) |
| Sepsis | 161 (8.2%) | 74 (7.1%) | 43 (15.6%) | 31 (4.1%) | 87 (9.4%) | 56 (13.9%) | 31 (6.0%) |
| Cardiovascular diseases, n (%) | 463 (23.6%) | 195 (18.8%) | 121 (43.8%) | 74 (9.7%) | 268 (29.0%) | 188 (46.7%) | 80 (15.4%) |
| Diseases of arteries, arterioles and capillaries | 119 (6.1%) | 51 (4.9%) | 38 (13.8%) | 13 (1.7%) | 68 (7.4%) | 54 (13.4%) | 14 (2.7%) |
| Pulmonary hearth disease | 103 (5.3%) | 47 (4.5%) | 34 (12.3%) | 13 (1.7%) | 56 (6.1%) | 50 (12.4%) | 6 (1.2%) |
| Arterial hypertension | 102 (5.2%) | 40 (3.9%) | 33 (12.0%) | 7 (0.9%) | 62 (6.7%) | 44 (10.9%) | 18 (3.5%) |
| Heart failure | 90 (4.6%) | 50 (4.8%) | 31 (11.2%) | 19 (2.5%) | 40 (4.3%) | 27 (6.7%) | 13 (2.5%) |
| Cerebral infraction | 47 (2.4%) | 19 (1.8%) | 7 (2.5%) | 12 (1.6%) | 28 (3.0%) | 15 (3.7%) | 13 (2.5%) |
| Acute and subacute endocarditis | 24 (1.2%) | 7 (0.7%) | 2 (0.7%) | 5 (0.7%) | 17 (1.8%) | 5 (1.2%) | 12 (2.3%) |
| Cardiomyopathy | 24 (1.2%) | 12 (1.2%) | 8 (2.9%) | 4 (0.5%) | 12 (1.3%) | 5 (1.2%) | 7 (1.3%) |
| Ischemic heart disease | 20 (1.0%) | 8 (0.8%) | 5 (1.8%) | 3 (0.4%) | 12 (1.3%) | 8 (2.0%) | 4 (0.8%) |
| Acute myocarditis | 4 (0.2%) | 3 (0.3%) | 1 (0.4%) | 2 (0.3%) | 1 (0.1%) | 0 (0.0%) | 1 (0.2%) |
| Chronic rheumatic heart disease | 1 (0.1%) | 1 (0.1%) | 1 (0.4%) | 0 | 0 | 0 | 0 |
| Metabolic disorders, n (%) | 374 (19.1%) | 171 (16.5%) | 106 (38.4%) | 65 (8.5%) | 203 (22.0%) | 143 (35.5%) | 60 (11.5%) |
| Haemorrhage, n (%) | 313 (16.0%) | 137 (13.2%) | 73 (26.4%) | 64 (8.4%) | 176 (19.0%) | 93 (23.1%) | 83 (15.9%) |
| Congenital heart disease, n (%) | 313 (16.0%) | 125 (12.1%) | 112 (40.6%) | 13 (1.7%) | 188 (20.3%) | 166 (41.2%) | 22 (4.2%) |
| Coagulation disturbances or thrombophilia, n (%) | 278 (14.2%) | 133 (12.8%) | 54 (19.6%) | 79 (10.4%) | 145 (15.7%) | 74 (18.4%) | 71 (13.6%) |
| Secondary thrombophilia | 97 (4.9%) | 37 (3.6%) | 25 (9.1%) | 12 (1.6%) | 60 (6.5%) | 42 (10.4%) | 18 (3.5%) |
| Hereditary deficiency of clotting factors | 40 (2.0%) | 13 (1.3%) | 2 (0.7%) | 11 (1.4%) | 27 (2.9%) | 11 (2.7%) | 16 (3.1%) |
| Primary thrombophilia | 38 (1.9%) | 22 (2.1%) | 2 (0.7%) | 20 (2.6%) | 16 (1.7%) | 1 (0.2%) | 15 (2.9%) |
| Cancer, n (%) | 211 (10.8%) | 90 (8.7%) | 37 (13.4%) | 53 (7.0%) | 121 (13.1%) | 35 (8.7%) | 86 (16.5%) |
| Venous thromboembolism not including PE or DVT, n (%) | 183 (9.3%) | 66 (6.4%) | 34 (12.3%) | 32 (4.2%) | 117 (12.7%) | 85 (21.1%) | 32 (6.1%) |
| Renal failure, n (%) | 160 (8.2%) | 87 (8.4%) | 55 (19.9%) | 32 (4.2%) | 73 (7.9%) | 44 (10.9%) | 29 (5.6%) |
| Malnutrition, n (%) | 56 (2.9%) | 23 (2.2%) | 14 (5.1%) | 9 (1.2%) | 33 (3.6%) | 8 (2.0%) | 25 (4.8%) |
| Venous thrombosis of the deep veins of the upper extremities, n (%) | 48 (2.4%) | 24 (2.3%) | 15 (5.4%) | 9 (1.2%) | 24 (2.6%) | 14 (3.5%) | 10 (1.9%) |
| Obesity, n (%) | 42 (2.1%) | 31 (3.0%) | 0 | 31 (4.1%) | 11 (1.2%) | 0 | 11 (2.1%) |
| Osteomyelitis, n (%) | 24 (1.2%) | 6 (0.6%) | 2 (0.7%) | 4 (0.5%) | 18 (1.9%) | 7 (1.7%) | 11 (2.1%) |
| Intracerebral haemorrhage, n (%) | 28 (1.4%) | 12 (1.2%) | 6 (2.2%) | 6 (0.8%) | 16 (1.7%) | 14 (3.5%) | 2 (0.4%) |
| Peritonitis, n (%) | 20 (1.0%) | 6 (0.6%) | 4 (1.4%) | 2 (0.3%) | 14 (1.5%) | 11 (2.7%) | 3 (0.6%) |
| Glomerular diseases, n (%) | 17 (0.9%) | 5 (0.5%) | 1 (0.4%) | 4 (0.5%) | 12 (1.3%) | 5 (1.2%) | 7 (1.3%) |
| Nephrotic syndrome | 5 (0.3%) | 0 | 0 | 0 | 5 (0.5%) | 3 (0.7%) | 2 (0.4%) |
| Corona virus disease 2019, n (%) | 16 (0.8%) | 7 (0.7%) | 1 (0.4%) | 6 (0.8%) | 9 (1.0%) | 2 (0.5%) | 7 (1.3%) |
| Diabetes mellitus, n (%) | 8 (0.4%) | 2 (0.2%) | 0 | 2 (0.3%) | 6 (0.6%) | 3 (0.7%) | 3 (0.6%) |
| Hyposplenism, n (%) | 3 (0.2%) | 1 (0.1%) | 1 (0.4%) | 0 | 2 (0.2%) | 0 | 2 (0.4%) |

n. a.: not applicable

# Table S28. Comorbidities in patients with pulmonary embolism and deep vein thrombosis stratified by age and sex.

| **Characteristic** | **Both diseases,** N = 1,961 | **Pulmonary embolism** | | | | | | | **Deep vein thrombosis (excluding cases with concomitant pulmonary embolism diagnosis)** | | | | | | |
| --- | --- | --- | --- | --- | --- | --- | --- | --- | --- | --- | --- | --- | --- | --- | --- |
|  |  | **Overall**, N = 815 | **Female** | | | **Male** | | | **Overall**, N = 1,146 | **Female** | | | **Male** | | |
|  |  |  | **Overall**, N = 497 | **0 to 9 years old**, N = 35 | **10 to 19 years old**, N = 462 | **Overall**, N = 318 | **0 to 9 years old**, N = 46 | **10 to 19 years old**, N = 272 |  | **Overall**, N = 540 | **0 to 9 years old**, N = 241 | **10 to 19 years old**, N = 299 | **Overall**, N = 606 | **0 to 9 years old**, N = 357 | **10 to 19 years old**, N = 249 |
| Injury or trauma, n (%) | 762 (38.9%) | 242 (29.7%) | 105 (21.1%) | 23 (65.7%) | 82 (17.7%) | 137 (43.1%) | 24 (52.2%) | 113 (41.5%) | 520 (45.4%) | 230 (42.6%) | 151 (62.7%) | 79 (26.4%) | 290 (47.9%) | 201 (56.3%) | 89 (35.7%) |
| Respiratory diseases, n (%) | 652 (33.2%) | 314 (38.5%) | 163 (32.8%) | 19 (54.3%) | 144 (31.2%) | 151 (47.5%) | 27 (58.7%) | 124 (45.6%) | 338 (29.5%) | 152 (28.1%) | 119 (49.4%) | 33 (11.0%) | 186 (30.7%) | 152 (42.6%) | 34 (13.7%) |
| Pneumonia | 185 (9.4%) | 91 (11.2%) | 43 (8.7%) | 6 (17.1%) | 37 (8.0%) | 48 (15.1%) | 13 (28.3%) | 35 (12.9%) | 94 (8.2%) | 43 (8.0%) | 35 (14.5%) | 8 (2.7%) | 51 (8.4%) | 39 (10.9%) | 12 (4.8%) |
| Respiratory failure | 172 (8.8%) | 69 (8.5%) | 27 (5.4%) | 7 (20.0%) | 20 (4.3%) | 42 (13.2%) | 8 (17.4%) | 34 (12.5%) | 103 (9.0%) | 47 (8.7%) | 40 (16.6%) | 7 (2.3%) | 56 (9.2%) | 49 (13.7%) | 7 (2.8%) |
| Asthma | 22 (1.1%) | 16 (2.0%) | 11 (2.2%) | 0 | 11 (2.4%) | 5 (1.6%) | 0 | 5 (1.8%) | 6 (0.5%) | 4 (0.7%) | 0 | 4 (1.3%) | 2 (0.3%) | 0 | 2 (0.8%) |
| Acute bronchiolitis | 20 (1.0%) | 5 (0.6%) | 2 (0.4%) | 1 (2.9%) | 1 (0.2%) | 2 (0.6%) | 1 (2.2%) | 1 (0.4%) | 15 (1.3%) | 5 (0.9%) | 5 (2.1%) | 0 | 9 (1.5%) | 9 (2.5%) | 0 |
| Acute bronchitis | 18 (0.9%) | 4 (0.5%) | 2 (0.4%) | 2 (5.7%) | 0 | 3 (0.9%) | 2 (4.3%) | 1 (0.4%) | 14 (1.2%) | 7 (1.3%) | 7 (2.9%) | 0 | 8 (1.3%) | 8 (2.2%) | 0 |
| Infectious diseases, n (%) | 535 (27.3%) | 176 (21.6%) | 80 (16.1%) | 19 (54.3%) | 61 (13.2%) | 96 (30.2%) | 22 (47.8%) | 74 (27.2%) | 359 (31.3%) | 157 (29.1%) | 116 (48.1%) | 41 (13.7%) | 202 (33.3%) | 155 (43.4%) | 47 (18.9%) |
| Sepsis | 161 (8.2%) | 65 (8.0%) | 32 (6.4%) | 11 (31.4%) | 21 (4.5%) | 33 (10.4%) | 10 (21.7%) | 23 (8.5%) | 96 (8.4%) | 42 (7.8%) | 32 (13.3%) | 10 (3.3%) | 54 (8.9%) | 46 (12.9%) | 8 (3.2%) |
| Cardiovascular diseases, n (%) | 463 (23.6%) | 137 (16.8%) | 67 (13.5%) | 15 (42.9%) | 52 (11.3%) | 70 (22.0%) | 22 (47.8%) | 48 (17.6%) | 326 (28.4%) | 128 (23.7%) | 106 (44.0%) | 22 (7.4%) | 198 (32.7%) | 166 (46.5%) | 32 (12.9%) |
| Diseases of arteries, arterioles and capillaries | 119 (6.1%) | 30 (3.7%) | 18 (3.6%) | 7 (20.0%) | 11 (2.4%) | 12 (3.8%) | 6 (13.0%) | 6 (2.2%) | 89 (7.8%) | 33 (6.1%) | 31 (12.9%) | 2 (0.7%) | 56 (9.2%) | 48 (13.4%) | 8 (3.2%) |
| Pulmonary hearth disease | 103 (5.3%) | 28 (3.4%) | 16 (3.2%) | 6 (17.1%) | 10 (2.2%) | 12 (3.8%) | 10 (21.7%) | 2 (0.7%) | 75 (6.5%) | 31 (5.7%) | 28 (11.6%) | 3 (1.0%) | 44 (7.3%) | 40 (11.2%) | 4 (1.6%) |
| Arterial hypertension | 102 (5.2%) | 21 (2.6%) | 9 (1.8%) | 6 (17.1%) | 3 (0.6%) | 12 (3.8%) | 4 (8.7%) | 8 (2.9%) | 81 (7.1%) | 31 (5.7%) | 27 (11.2%) | 4 (1.3%) | 50 (8.3%) | 40 (11.2%) | 10 (4.0%) |
| Heart failure | 90 (4.6%) | 28 (3.4%) | 18 (3.6%) | 4 (11.4%) | 14 (3.0%) | 10 (3.1%) | 1 (2.2%) | 9 (3.3%) | 62 (5.4%) | 32 (5.9%) | 27 (11.2%) | 5 (1.7%) | 30 (5.0%) | 26 (7.3%) | 4 (1.6%) |
| Cerebral infraction | 47 (2.4%) | 13 (1.6%) | 6 (1.2%) | 1 (2.9%) | 5 (1.1%) | 7 (2.2%) | 2 (4.3%) | 5 (1.8%) | 34 (3.0%) | 13 (2.4%) | 6 (2.5%) | 7 (2.3%) | 21 (3.5%) | 13 (3.6%) | 8 (3.2%) |
| Acute and subacute endocarditis | 24 (1.2%) | 13 (1.6%) | 6 (1.2%) | 1 (2.9%) | 5 (1.1%) | 7 (2.2%) | 0 | 7 (2.6%) | 11 (1.0%) | 1 (0.2%) | 1 (0.4%) | 0 | 10 (1.7%) | 5 (1.4%) | 5 (2.0%) |
| Cardiomyopathy | 24 (1.2%) | 9 (1.1%) | 2 (0.4%) | 2 (5.7%) | 0 | 7 (2.2%) | 1 (2.2%) | 6 (2.2%) | 15 (1.3%) | 10 (1.9%) | 6 (2.5%) | 4 (1.3%) | 5 (0.8%) | 4 (1.1%) | 1 (0.4%) |
| Ischemic heart disease | 20 (1.0%) | 6 (0.7%) | 3 (0.6%) | 1 (2.9%) | 2 (0.4%) | 3 (0.9%) | 2 (4.3%) | 1 (0.4%) | 14 (1.2%) | 5 (0.9%) | 4 (1.7%) | 1 (0.3%) | 9 (1.5%) | 6 (1.7%) | 3 (1.2%) |
| Acute myocarditis | 4 (0.2%) | 1 (0.1%) | 1 (0.2%) | 0 (0.0%) | 1 (0.2%) | 0 | 0 | 0 | 3 (0.3%) | 2 (0.4%) | 1 (0.4%) | 1 (0.3%) | 1 (0.2%) | 0 | 1 (0.4%) |
| Chronic rheumatic heart disease | 1 (0.1%) | 0 | 0 | 0 | 0 | 0 | 0 | 0 | 1 (0.1%) | 1 (0.2%) | 1 (0.4%) | 0 | 0 | 0 | 0 |
| Metabolic disorders, n (%) | 374 (19.1%) | 115 (14.1%) | 62 (12.5%) | 20 (57.1%) | 42 (9.1%) | 53 (16.7%) | 18 (39.1%) | 35 (12.9%) | 259 (22.6%) | 109 (20.2%) | 86 (35.7%) | 23 (7.7%) | 150 (24.8%) | 125 (35.0%) | 25 (10.0%) |
| Haemorrhage, n (%) | 313 (16.0%) | 118 (14.5%) | 48 (9.7%) | 12 (34.3%) | 36 (7.8%) | 70 (22.0%) | 13 (28.3%) | 57 (21.0%) | 195 (17.0%) | 89 (16.5%) | 61 (25.3%) | 28 (9.4%) | 106 (17.5%) | 80 (22.4%) | 26 (10.4%) |
| Congenital heart disease, n (%) | 313 (16.0%) | 52 (6.4%) | 25 (5.0%) | 16 (45.7%) | 9 (1.9%) | 27 (8.5%) | 16 (34.8%) | 11 (4.0%) | 261 (22.8%) | 100 (18.5%) | 96 (39.8%) | 4 (1.3%) | 161 (26.6%) | 150 (42.0%) | 11 (4.4%) |
| Coagulation disturbances or thrombophilia, n (%) | 278 (14.2%) | 102 (12.5%) | 47 (9.5%) | 10 (28.6%) | 37 (8.0%) | 55 (17.3%) | 8 (17.4%) | 47 (17.3%) | 176 (15.4%) | 86 (15.9%) | 44 (18.3%) | 42 (14.0%) | 90 (14.9%) | 66 (18.5%) | 24 (9.6%) |
| Secondary thrombophilia | 97 (4.9%) | 30 (3.7%) | 12 (2.4%) | 4 (11.4%) | 8 (1.7%) | 18 (5.7%) | 4 (8.7%) | 14 (5.1%) | 67 (5.8%) | 25 (4.6%) | 21 (8.7%) | 4 (1.3%) | 42 (6.9%) | 38 (10.6%) | 4 (1.6%) |
| Hereditary deficiency of clotting factors | 40 (2.0%) | 13 (1.6%) | 3 (0.6%) | 1 (2.9%) | 2 (0.4%) | 10 (3.1%) | 1 (2.2%) | 9 (3.3%) | 27 (2.4%) | 10 (1.9%) | 1 (0.4%) | 9 (3.0%) | 17 (2.8%) | 10 (2.8%) | 7 (2.8%) |
| Primary thrombophilia | 38 (1.9%) | 18 (2.2%) | 7 (1.4%) | 0 | 7 (1.5%) | 11 (3.5%) | 0 | 11 (4.0%) | 20 (1.7%) | 15 (2.8%) | 2 (0.8%) | 13 (4.3%) | 5 (0.8%) | 1 (0.3%) | 4 (1.6%) |
| Cancer, n (%) | 211 (10.8%) | 70 (8.6%) | 30 (6.0%) | 6 (17.1%) | 24 (5.2%) | 40 (12.6%) | 8 (17.4%) | 32 (11.8%) | 141 (12.3%) | 60 (11.1%) | 31 (12.9%) | 29 (9.7%) | 81 (13.4%) | 27 (7.6%) | 54 (21.7%) |
| Venous thromboembolism not including PE or DVT, n (%) | 183 (9.3%) | 49 (6.0%) | 19 (3.8%) | 2 (5.7%) | 17 (3.7%) | 30 (9.4%) | 11 (23.9%) | 19 (7.0%) | 134 (11.7%) | 47 (8.7%) | 32 (13.3%) | 15 (5.0%) | 87 (14.4%) | 74 (20.7%) | 13 (5.2%) |
| Renal failure, n (%) | 160 (8.2%) | 55 (6.7%) | 31 (6.2%) | 8 (22.9%) | 23 (5.0%) | 24 (7.5%) | 6 (13.0%) | 18 (6.6%) | 105 (9.2%) | 56 (10.4%) | 47 (19.5%) | 9 (3.0%) | 49 (8.1%) | 38 (10.6%) | 11 (4.4%) |
| Malnutrition, n (%) | 56 (2.9%) | 26 (3.2%) | 9 (1.8%) | 4 (11.4%) | 5 (1.1%) | 17 (5.3%) | 2 (4.3%) | 15 (5.5%) | 30 (2.6%) | 14 (2.6%) | 10 (4.1%) | 4 (1.3%) | 16 (2.6%) | 6 (1.7%) | 10 (4.0%) |
| Venous thrombosis of the deep veins of the upper extremities, n (%) | 48 (2.4%) | 12 (1.5%) | 6 (1.2%) | 2 (5.7%) | 4 (0.9%) | 6 (1.9%) | 2 (2.4%) | 4 (1.5%) | 36 (3.1%) | 18 (3.3%) | 13 (5.4%) | 5 (1.7%) | 18 (3.0%) | 12 (3.4%) | 6 (2.4%) |
| Obesity, n (%) | 42 (2.1%) | 26 (3.2%) | 20 (4.0%) | 0 | 20 (4.3%) | 6 (1.9%) | 0 | 6 (2.2%) | 16 (1.4%) | 11 (2.0%) | 0 | 11 (3.7%) | 5 (0.8%) | 0 | 5 (2.0%) |
| Osteomyelitis, n (%) | 24 (1.2%) | 9 (1.1%) | 4 (0.8%) | 2 (5.7%) | 2 (0.4%) | 5 (1.6%) | 0 | 5 (1.8%) | 15 (1.3%) | 2 (0.4%) | 0 | 2 (0.7%) | 13 (2.1%) | 7 (2.0%) | 6 (2.4%) |
| Intracerebral haemorrhage, n (%) | 28 (1.4%) | 10 (1.2%) | 6 (1.2%) | 1 (2.9%) | 5 (1.1%) | 4 (1.3%) | 3 (6.5%) | 1 (0.4%) | 18 (1.6%) | 6 (1.1%) | 5 (2.1%) | 1 (0.3%) | 12 (2.0%) | 11 (3.1%) | 1 (0.4%) |
| Peritonitis, n (%) | 20 (1.0%) | 6 (0.7%) | 3 (0.6%) | 1 (2.9%) | 2 (0.4%) | 3 (0.9%) | 1 (2.2%) | 2 (0.7%) | 14 (1.2%) | 3 (0.6%) | 3 (1.2%) | 0 | 11 (1.8%) | 10 (2.8%) | 1 (0.4%) |
| Glomerular diseases, n (%) | 17 (0.9%) | 8 (1.0%) | 1 (0.2%) | 0 | 1 (0.2%) | 7 (2.2%) | 2 (4.3%) | 5 (1.8%) | 9 (0.8%) | 4 (0.7%) | 1 (0.4%) | 3 (1.0%) | 5 (0.8%) | 3 (0.8%) | 2 (0.8%) |
| Nephrotic syndrome | 5 (0.3%) | 3 (0.4%) | 0 | 0 | 0 | 3 (0.9%) | 1 (2.2%) | 2 (0.7%) | 2 (0.2%) | 0 | 0 | 0 | 2 (0.3%) | 2 (0.6%) | 0 |
| Corona virus disease 2019, n (%) | 16 (0.8%) | 9 (1.1%) | 3 (0.6%) | 0 | 3 (0.6%) | 6 (1.9%) | 1 (2.2%) | 5 (1.8%) | 7 (0.6%) | 4 (0.7%) | 1 (0.4%) | 3 (1.0%) | 3 (0.5%) | 1 (0.3%) | 2 (0.8%) |
| Diabetes mellitus, n (%) | 8 (0.4%) | 4 (0.5%) | 1 (0.2%) | 0 | 1 (0.2%) | 3 (0.9%) | 0 | 3 (1.1%) | 4 (0.3%) | 1 (0.2%) | 0 | 1 (0.3%) | 3 (0.5%) | 3 (0.8%) | 0 |
| Hyposplenism, n (%) | 3 (0.2%) | 2 (0.2%) | 0 | 0 | 0 | 2 (0.6%) | 0 | 2 (0.7%) | 1 (0.1%) | 1 (0.2%) | 1 (0.4%) | 0 | 0 | 0 | 0 |

# Table S29. Length of hospitalization ([Q1-Q3] days) stratified by age and sex.

| **Venous thromboembolism** | | | |
| --- | --- | --- | --- |
|  | **Overall** | **Female** | **Male** |
| Age (years) |  |  |  |
| 0 years old | 34 (16-84) | 44.5 (16-94) | 31 (15-79) |
| 1-4 years old | 13 (3-33) | 15.5 (7-43.75) | 10.5 (3-23) |
| 5-9 years old | 11.5 (3-23.25) | 15 (5-31.75) | 8 (3-17.75) |
| 10-14 years old | 8 (2.75-21) | 7.5 (2.75-20) | 8.5 (2.75-23.5) |
| 15-19 years old | 5 (2-11) | 4 (2-8) | 7 (3-17) |
| Overall | 8 (3-24) | 6 (3-17) | 12 (4-31) |
|  |  |  |  |
| **Pulmonary embolism (with or without deep vein thrombosis)** | | | |
|  | **Overall** | **Female** | **Male** |
| Age (years) |  |  |  |
| 0 years old | 39 (9-152) | 22 (5.5-140.5) | 63 (29.75-161) |
| 1-4 years old | 19 (9-40) | 30.5 (16.25-118.25) | 17 (2-23) |
| 5-9 years old | 13 (8.5-32.5) | 34.5 (14.5-48.5) | 10 (7-14) |
| 10-14 years old | 12.5 (4-26.25) | 9 (3.75-29.25) | 13 (5.5-26.25) |
| 15-19 years old | 6 (4-12) | 5 (3-8) | 9 (4-20) |
| Overall | 6 (3-15) | 5 (3-9) | 10 (4-22) |
|  |  |  |  |
| **Deep vein thrombosis (excluding cases with concomitant pulmonary embolism diagnosis)** | | | |
|  | **Overall** | **Female** | **Male** |
| Age (years) |  |  |  |
| 0 years old | 34 (16-80.5) | 45 (18-89) | 31 (15-74.5) |
| 1-4 years old | 13 (3-31) | 14.5 (5-42.25) | 9 (3-23) |
| 5-9 years old | 11 (3-23) | 15 (3.75-29.25) | 8 (3-20) |
| 10-14 years old | 7 (2-19) | 7 (2-16.75) | 8 (2-19.25) |
| 15-19 years old | 5 (2-9.25) | 4 (2-8) | 6 (2-13) |
| Overall | 11 (3-32) | 8 (3-30) | 13 (4-34) |

# Table S30. Length of hospitalization ([Q1-Q3] days) stratified by clinically selected age groups and sex.

| **Venous thromboembolism** | | | |
| --- | --- | --- | --- |
|  | **Overall** | **Female** | **Male** |
| Age (years) |  |  |  |
| 0 years old | 34 (16-84) | 44.5 (16-94) | 31 (15-79) |
| 1-5 years old | 14 (3-34) | 17.5 (7.75-44.5) | 10.5 (3-23) |
| 6-12 years old | 10 (3-23) | 15 (4-31.5) | 8 (3-18.75) |
| 13-17 years old | 5 (2-12) | 5 (2-8) | 7 (2-17.5) |
| Overall | 11 (4-32) | 8 (3-27.5) | 13 (5-34) |
|  |  |  |  |
| **Pulmonary embolism (with or without deep vein thrombosis)** | | | |
|  | **Overall** | **Female** | **Male** |
| Age (years) |  |  |  |
| 0 years old | 39 (9-151) | 22 (5-139.5) | 63 (29.5-160.25) |
| 1-5 years old | 17.5 (9.25-39.25) | 30 (15.25-117.25) | 13.5 (2-22) |
| 6-12 years old | 13 (6-31) | 17 (10-44) | 8 (4.5-14.5) |
| 13-17 years old | 6 (3-12) | 5 (2.5-8) | 11 (4-22.5) |
| Overall | 7 (3-18.25) | 6 (3-13) | 11 (5-26) |
|  |  |  |  |
| **Deep vein thrombosis (excluding cases with concomitant pulmonary embolism diagnosis)** | | | |
|  | **Overall** | **Female** | **Male** |
| Age (years) |  |  |  |
| 0 years old | 34 (16-80.5) | 45 (18-89) | 31 (15-74.5) |
| 1-5 years old | 13 (3-33) | 17 (5.5-42.75) | 10 (3-23.5) |
| 6-12 years old | 9 (3-21) | 10 (3-24) | 8.5 (3-18.75) |
| 13-17 years old | 5 (2-11) | 5 (2-8.75) | 6 (2-13.25) |
| Overall | 14 (4-40) | 11 (4-42) | 15 (5-36) |

# Table S31. Univariable logistical regression models for selected clinical outcomes.

|  | Death, OR (95% CI) | Intracranial haemorrhage, OR (95% CI) | Haemorrhage not including intracranial haemorrhage, OR (95% CI) | Intensive care unit admission, OR (95% CI) |
| --- | --- | --- | --- | --- |
| Overall cohort | | | | |
| Invasive ventilation | 9.58 (5.98; 15.69) | 4.48 (2.1; 9.55) | 5.16 (3.96; 6.71) | 61.32 (37.95; 106.64) |
| Use of vasopressors | 7.71 (4.78; 12.34) | 5.04 (2.21; 10.87) | 4.76 (3.47; 6.49) | 102.46 (43.23; 333.57) |
| Use of systemic thromboliytics | 3.91 (2.19; 6.68) | 3.39 (1.23; 8) | 2.21 (1.5; 3.21) | 1.65 (1.18; 2.3) |
| Extracorporeal membrane oxygenation | 21.49 (12.19; 37.54) | 15.05 (6.25; 33.84) | 13.69 (8.16; 23.71) | 113.16 (24.98; 2000.36) |
| Surgical thrombectomy | 4.42 (0.68; 16.82) | 5.93 (0.32; 31.76) | 6.26 (2.07; 19.57) | 5.2 (1.58; 23.25) |
| Presence of high-risk features | 20.26 (10.6; 43.82) | 7.16 (3.17; 18.25) | 6.08 (4.7; 7.89) | 20.21 (15.73; 26.19) |
|  |  |  |  |  |
| Catheter directed intervention | 1.32 (0.4; 3.29) | 0 (0; inf) | 1.18 (0.63; 2.07) | 0.53 (0.31; 0.87) |
| Placement of an inferior vena cava filter | 0 (0; inf) | 2.27 (0.13; 11.22) | 4.24 (2.05; 8.59) | 3 (1.47; 6.49) |
| Transfusions of blood or blood components | 13.4 (7.79; 24.7) | 6.07 (2.80; 14.2) | 14.2 (10.7; 19.0) | 23.6 (17.9; 31.6) |
| Pulmonary embolism (vs. deep vein thrombosis) | 1.12 (0.71; 1.77) | 0.78 (0.34; 1.66) | 0.83 (0.64; 1.06) | 0.62 (0.51; 0.75) |
| Admission to an intensive care unit | 20.6 (9.71; 53.4) | 13.3 (4.63; 55.8) | 7.69 (5.80; 10.3) | - |
|  |  |  |  |  |
| Patients aged 0-9 years | | | | |
| Invasive ventilation | 5.62 (2.93; 11.69) | 2.04 (0.83; 5.28) | 2.92 (2.04; 4.22) | 29.76 (15.74; 63.94) |
| Use of vasopressors | 3.86 (2.16; 6.9) | 2.32 (0.89; 5.72) | 2.49 (1.69; 3.66) | 30.03 (11.19; 122.77) |
| Use of systemic thromboliytics | 5.47 (2.61; 10.97) | 2.35 (0.54; 7.32) | 2.29 (1.24; 4.14) | 2.61 (1.23; 6.45) |
| Extracorporeal membrane oxygenation | 6.87 (3.23; 14.07) | 7.42 (2.51; 19.7) | 8.14 (4.2; 16.6) | inf (0; inf) |
| Surgical thrombectomy | 2.03 (0.11; 12.2) | 0 (0; inf) | 2.34 (0.46; 10.73) | 2.49 (0.42; 47.16) |
| Presence of high-risk features | 15.27 (5.53; 63.28) | 1.95 (0.77; 5.56) | 3.68 (2.49; 5.56) | 30.32 (18.2; 53.63) |
|  |  |  |  |  |
| Catheter directed intervention | 3.54 (0.52; 15.12) | 0 (0; inf) | 3.95 (1.03; 16.13) | 1.45 (0.35; 9.77) |
| Placement of an inferior vena cava filter | 0 (0; inf) | 0 (0; inf) | 0 (0; inf) | 0 (0; inf) |
| Transfusions of blood or blood components | 6.53 (3.09; 16.1) | 2.16 (0.86; 6.17) | 9.29 (5.85; 15.4) | 16.2 (10.3; 26.4) |
| Pulmonary embolism (vs. deep vein thrombosis) | 3.45 (1.75; 6.51) | 1.89 (0.53; 5.31) | 1.45 (0.86; 2.38) | 1.12 (0.67; 1.92) |
| Admission to an intensive care unit | 23.4 (5.07; 415) | 8.10 (1.66; 146) | 6.07 (3.53; 11.3) | - |
|  |  |  |  |  |
| Patients aged 10-19 years | | | | |
| Invasive ventilation | 12.59 (5.48; 28.01) | 5.41 (0.78; 23.93) | 9.45 (5.78; 15.46) | 43.93 (21.34; 106.31) |
| Use of vasopressors | 13.13 (5.15; 30.94) | 9.1 (1.31; 40.79) | 9.43 (5.16; 17.3) | 186.83 (40.57; 3317.7) |
| Use of systemic thromboliytics | 2.74 (0.9; 6.87) | 7.2 (1.46; 29.78) | 2.35 (1.39; 3.85) | 1.83 (1.18; 2.81) |
| Extracorporeal membrane oxygenation | 88.71 (35.18; 230.12) | 32.63 (6.4; 141.29) | 19.66 (8.65; 48.74) | 93.49 (19.69; 1674.03) |
| Surgical thrombectomy | 9.62 (0.49; 62.44) | 36.26 (1.77; 268.13) | 15.85 (3.07; 115.04) | 6.92 (1.34; 50.12) |
| Presence of high-risk features | 18.68 (7.9; 51.44) | 35.07 (6.2; 657.19) | 7.43 (5.14; 10.75) | 10.49 (7.61; 14.57) |
|  |  |  |  |  |
| Catheter directed intervention | 1.44 (0.23; 4.98) | 0 (0; inf) | 1.19 (0.54; 2.34) | 0.8 (0.41; 1.44) |
| Placement of an inferior vena cava filter | 0 (0; inf) | 5.92 (0.31; 34.79) | 6.92 (3.29; 14.35) | 7.67 (3.65; 17.18) |
| Transfusions of blood or blood components | 18.5 (8.21; 45.5) | 11.9 (2.88; 58.3) | 23.3 (15.6; 35.3) | 15.4 (10.5; 22.8) |
| Pulmonary embolism (vs. deep vein thrombosis) | 2.16 (0.95; 5.55) | 2.25 (0.52; 15.4) | 1.33 (0.93; 1.9) | 2.25 (1.7; 3) |
| Admission to an intensive care unit | 16.2 (6.57; 48.7) | 10.5 (2.39; 71.6) | 8.20 (5.69; 11.9) | - |

# Table S32. Multivariable logistical regression models adjusted for sex for selected clinical outcomes.

|  | Death, OR (95% CI) | Intracranial haemorrhage, OR (95% CI) | Haemorrhage not including intracranial haemorrhage, OR (95% CI) | Intensive care unit admission, OR (95% CI) |
| --- | --- | --- | --- | --- |
| Overall cohort | | | | |
| Invasive ventilation | 9.84 (6.12; 16.19) | 4.34 (2.02; 9.31) | 5 (3.84; 6.52) | 60.4 (37.33; 105.14) |
| Use of vasopressors | 7.84 (4.84; 12.6) | 4.87 (2.13; 10.56) | 4.6 (3.35; 6.28) | 100.77 (42.47; 328.3) |
| Use of systemic thromboliytics | 3.97 (2.22; 6.81) | 3.6 (1.3; 8.56) | 2.36 (1.6; 3.44) | 1.8 (1.28; 2.53) |
| Extracorporeal membrane oxygenation | 21.51 (12.2; 37.59) | 14.86 (6.17; 33.47) | 13.79 (8.19; 23.95) | 116.46 (25.64; 2060.26) |
| Surgical thrombectomy | 4.44 (0.68; 16.9) | 6.17 (0.33; 33.32) | 6.63 (2.17; 20.87) | 5.66 (1.71; 25.47) |
| Presence of high-risk features | 20.53 (10.72; 44.44) | 7.01 (3.1; 17.91) | 5.96 (4.61; 7.75) | 20.53 (15.93; 26.7) |
|  |  |  |  |  |
| Catheter directed intervention | 1.33 (0.4; 3.33) | 0 (0; inf) | 1.26 (0.67; 2.23) | 0.57 (0.33; 0.95) |
| Placement of an inferior vena cava filter | 0 (0; inf) | 2.22 (0.12; 10.98) | 4.18 (2.02; 8.51) | 2.96 (1.44; 6.45) |
| Transfusions of blood or blood components | 13.8 (800; 25.5) | 5.92 (2.72; 13.9) | 13.9 (10.5; 18.6) | 23.2 (17.5; 31.1) |
| Pulmonary embolism (vs. deep vein thrombosis) | 1.13 (0.71; 1.79) | 0.82 (0.36; 1.77) | 0.87 (0.68; 1.12) | 0.66 (0.55; 0.8) |
| Admission to an intensive care unit | 21.6 (10.1; 56.1) | 13.0 (4.53; 55.1) | 7.49 (5.64; 10.1) | - |
|  |  |  |  |  |
| Patients aged 0-9 years | | | | |
| Invasive ventilation | 5.62 (2.93; 11.68) | 2.05 (0.84; 5.31) | 2.92 (2.04; 4.22) | 29.9 (15.81; 64.28) |
| Use of vasopressors | 3.89 (2.18; 6.95) | 2.31 (0.89; 5.69) | 2.5 (1.7; 3.69) | 30.35 (11.3; 124.11) |
| Use of systemic thromboliytics | 5.43 (2.59; 10.89) | 2.41 (0.55; 7.53) | 2.27 (1.23; 4.11) | 2.59 (1.21; 6.4) |
| Extracorporeal membrane oxygenation | 6.96 (3.27; 14.28) | 7.37 (2.48; 19.62) | 8.23 (4.25; 16.8) | inf (0; inf) |
| Surgical thrombectomy | 1.95 (0.1; 11.78) | 0 (0; inf) | 2.28 (0.44; 10.46) | 2.4 (0.41; 45.6) |
| Presence of high-risk features | 15.21 (5.51; 63.02) | 1.98 (0.78; 5.64) | 3.67 (2.48; 5.55) | 30.36 (18.21; 53.71) |
|  |  |  |  |  |
| Catheter directed intervention | 3.52 (0.51; 15.05) | 0 (0; inf) | 3.93 (1.03; 16.08) | 1.44 (0.34; 9.71) |
| Placement of an inferior vena cava filter | 0 (0; inf) | 0 (0; inf) | 0 (0; inf) | 0 (0; inf) |
| Transfusions of blood or blood components | 6.48 (3.06; 15.9) | 2.22 (0.88; 6.34) | 9.25 (5.83; 15.3) | 16.1 (10.2, 26.2) |
| Pulmonary embolism (vs. deep vein thrombosis) | 3.43 (1.74; 6.48) | 1.92 (0.54; 5.4) | 1.44 (0.85; 2.37) | 1.11 (0.67; 1.91) |
| Admission to an intensive care unit | 23.2 (5.02; 411) | 8.34 (1.71; 150) | 6.04 (3.51; 11.2) | - |
|  |  |  |  |  |
| Patients aged 10-19 years | | | | |
| Invasive ventilation | 13.03 (5.64; 29.19) | 5.84 (0.84; 26.12) | 9.24 (5.61; 15.23) | 44.54 (21.52; 108.21) |
| Use of vasopressors | 13.46 (5.26; 31.9) | 9.76 (1.4; 44.23) | 9.34 (5.06; 17.3) | 192.57 (41.62; 3424.5) |
| Use of systemic thromboliytics | 2.71 (0.89; 6.83) | 6.69 (1.34; 28.05) | 2.73 (1.59; 4.52) | 2.08 (1.32; 3.22) |
| Extracorporeal membrane oxygenation | 88.5 (35.06; 229.86) | 31.75 (6.2; 138.06) | 22.73 (9.82; 57.33) | 106.67 (22.28; 1914.74) |
| Surgical thrombectomy | 9.52 (0.49; 61.92) | 35.13 (1.71; 264.23) | 18.03 (3.4; 133.12) | 7.62 (1.45; 55.84) |
| Presence of high-risk features | 18.83 (7.95; 51.86) | 35.93 (6.34; 673.75) | 7.58 (5.22; 11.03) | 11.04 (7.94; 15.46) |
|  |  |  |  |  |
| Catheter directed intervention | 1.42 (0.22; 4.92) | 0 (0; inf) | 1.31 (0.59; 2.59) | 0.86 (0.44; 1.57) |
| Placement of an inferior vena cava filter | 0 (0; inf) | 6.63 (0.35; 39.86) | 6.51 (3.06; 13.64) | 7.31 (3.45; 16.51) |
| Transfusions of blood or blood components | 20.0 (8.81; 49.9) | 13.6 (3.27; 67.7) | 22.4 (14.9; 34.0) | 14.8 (10.1, 22.0) |
| Pulmonary embolism (vs. deep vein thrombosis) | 2.15 (0.94; 5.52) | 2.14 (0.49; 14.68) | 1.42 (1; 2.05) | 2.45 (1.84; 3.28) |
| Admission to an intensive care unit | 17.8 (7.15; 54.0) | 12.3 (2.76; 84.7) | 7.69 (5.32; 11.2) | - |

No adjustment besides for sex was performed due to low numbers of outcomes.

# Figure S1. Pulmonary embolism (PE)-related and deep vein thrombosis (DVT)-related incidence rate (PE / DVT incident hospital admissions per 100,000 children and adolescents per year) across age groups stratified by sex.


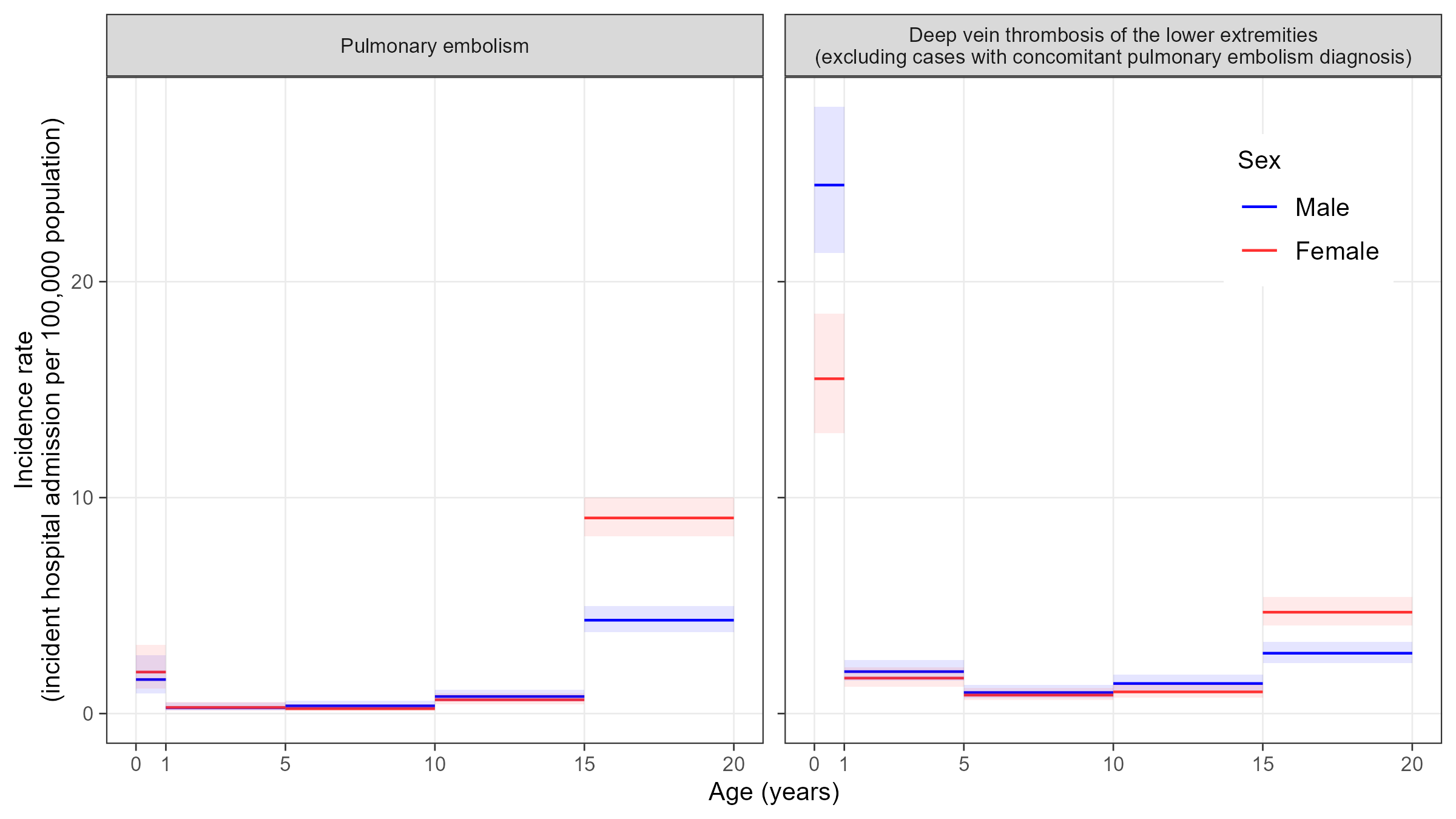


The shaded area depicts the 95% confidence interval.

# Figure S2. Venous thromboembolism (VTE)-related, pulmonary embolism (PE)-related and deep vein thrombosis (DVT)-related incidence rate (VTE / PE / DVT-related hospital admissions per 100,000 children and adolescents per year) across age groups stratified by sex.


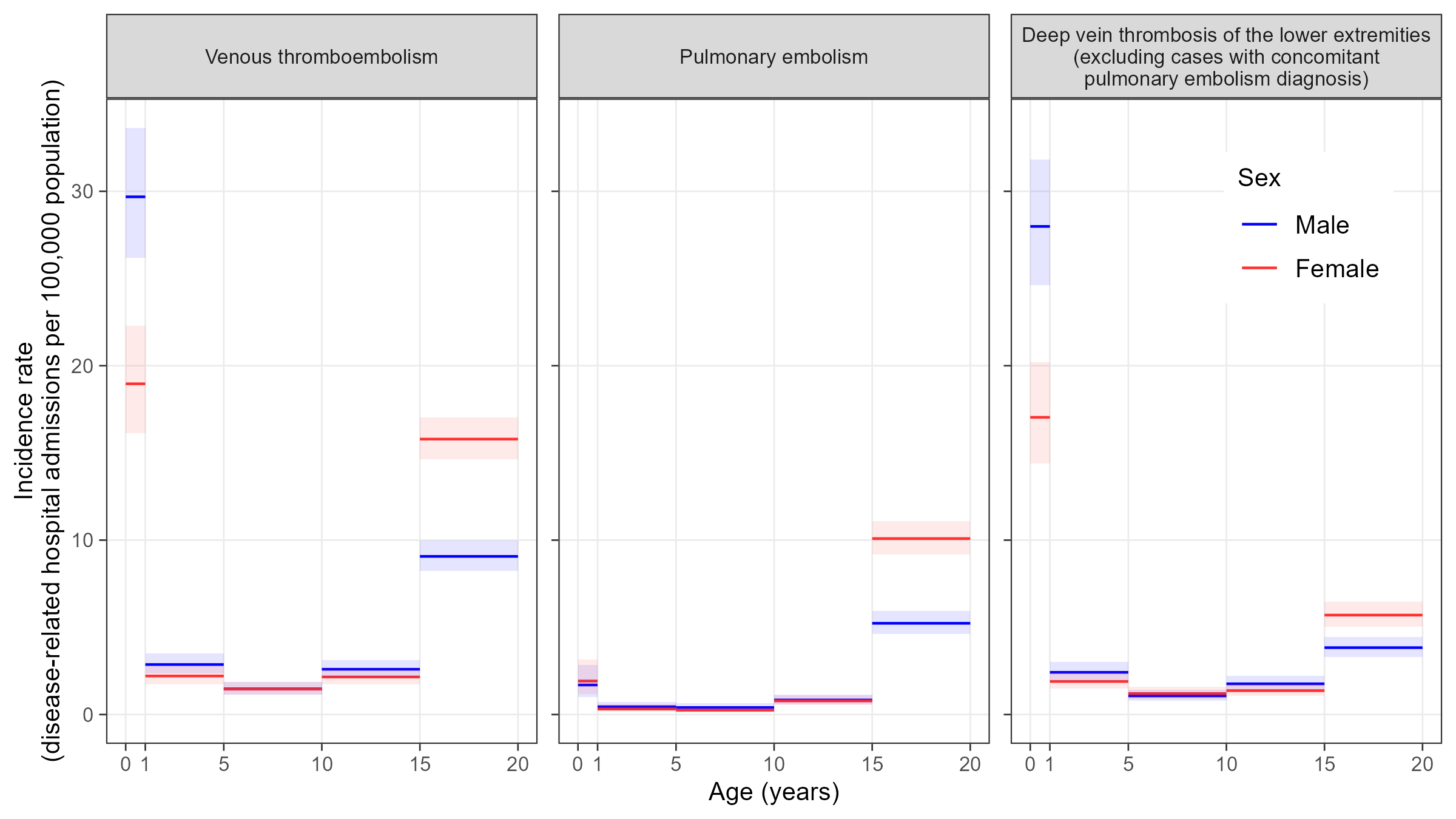


The shaded area depicts the 95% confidence interval.

# Figure S3. Proportion of venous thromboembolism (VTE)-related, pulmonary embolism (PE)-related, and deep vein thrombosis (DVT)-related hospitalizations out of all hospitalizations (VTE / PE / DVT-related hospital admissions per 10,000 hospital admissions) across age groups in male and female patients.


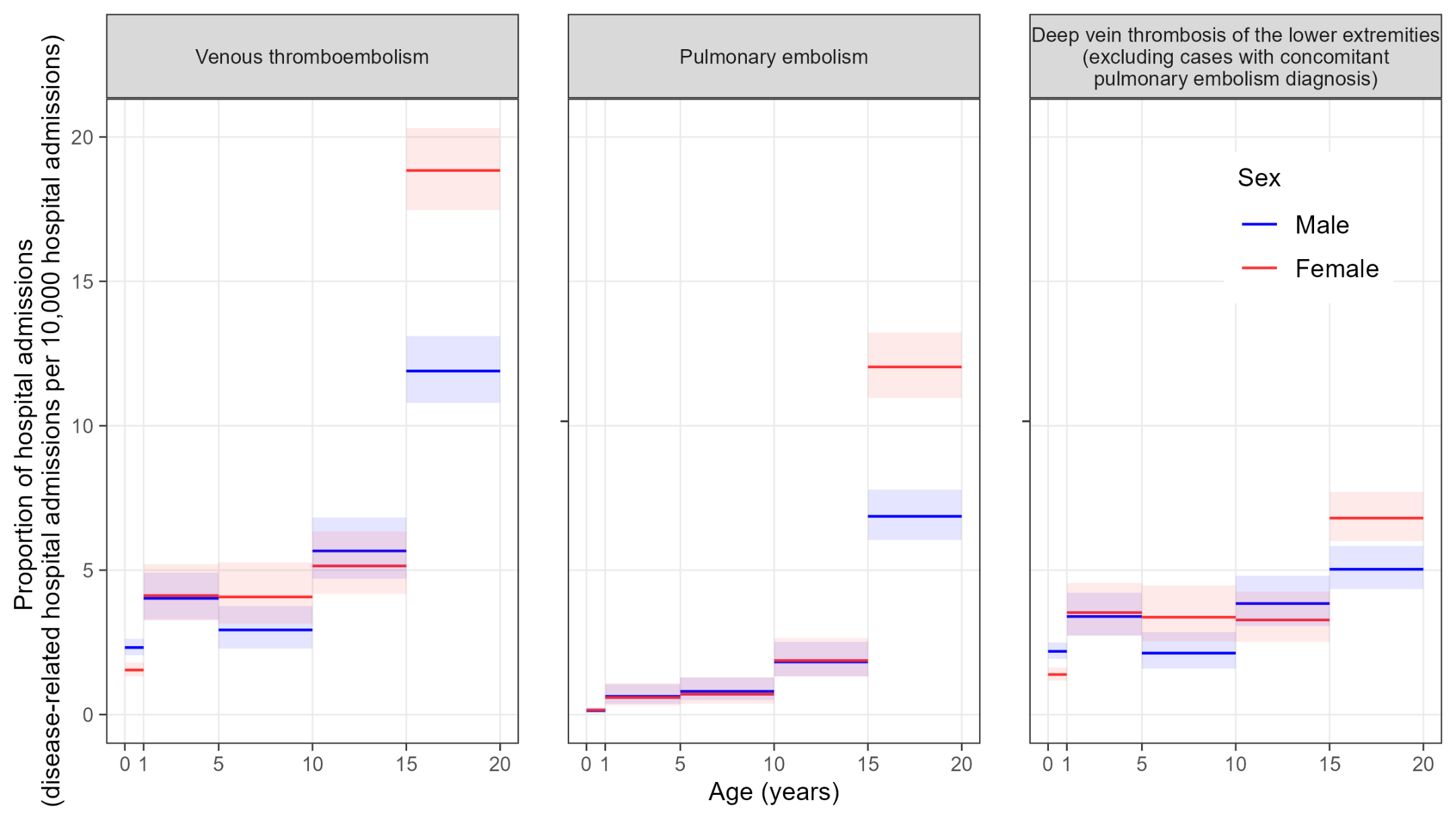
The shaded area depicts the 95% confidence interval.

# Figure S4. Venous thromboembolism (VTE)-related, pulmonary embolism (PE)-related and deep vein thrombosis (DVT)-related incidence rate (VTE / PE / DVT incident hospital admissions per 100,000 children and adolescents per year) across clinically selected age groups stratified by sex.


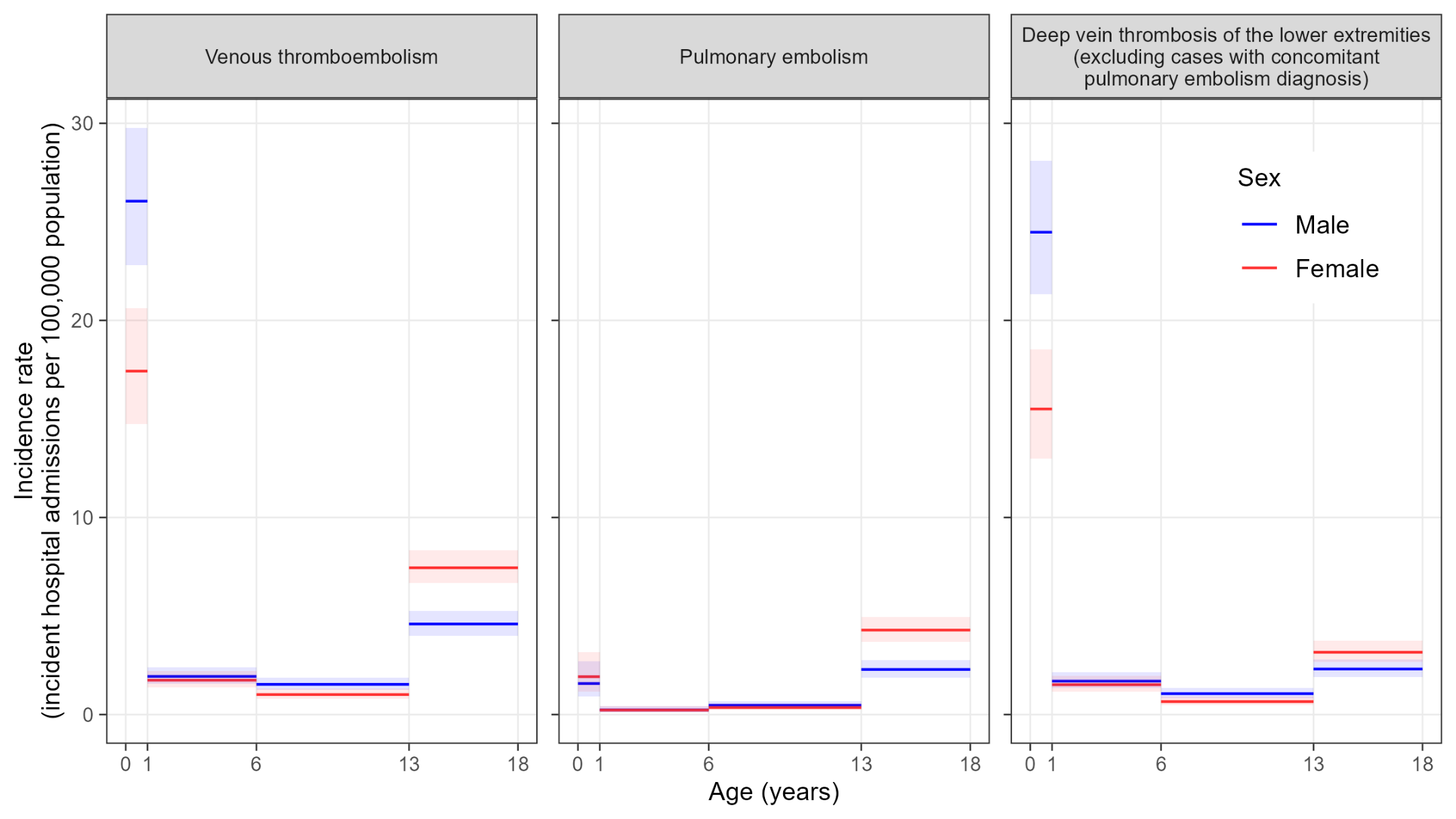
The shaded area depicts the 95% confidence interval.

# Figure S5. Venous thromboembolism (VTE)-related, pulmonary embolism (PE)-related and deep vein thrombosis (DVT)-related incidence rate (VTE / PE / DVT-related hospital admissions per 100,000 children and adolescents per year) across clinically selected age groups stratified by sex.


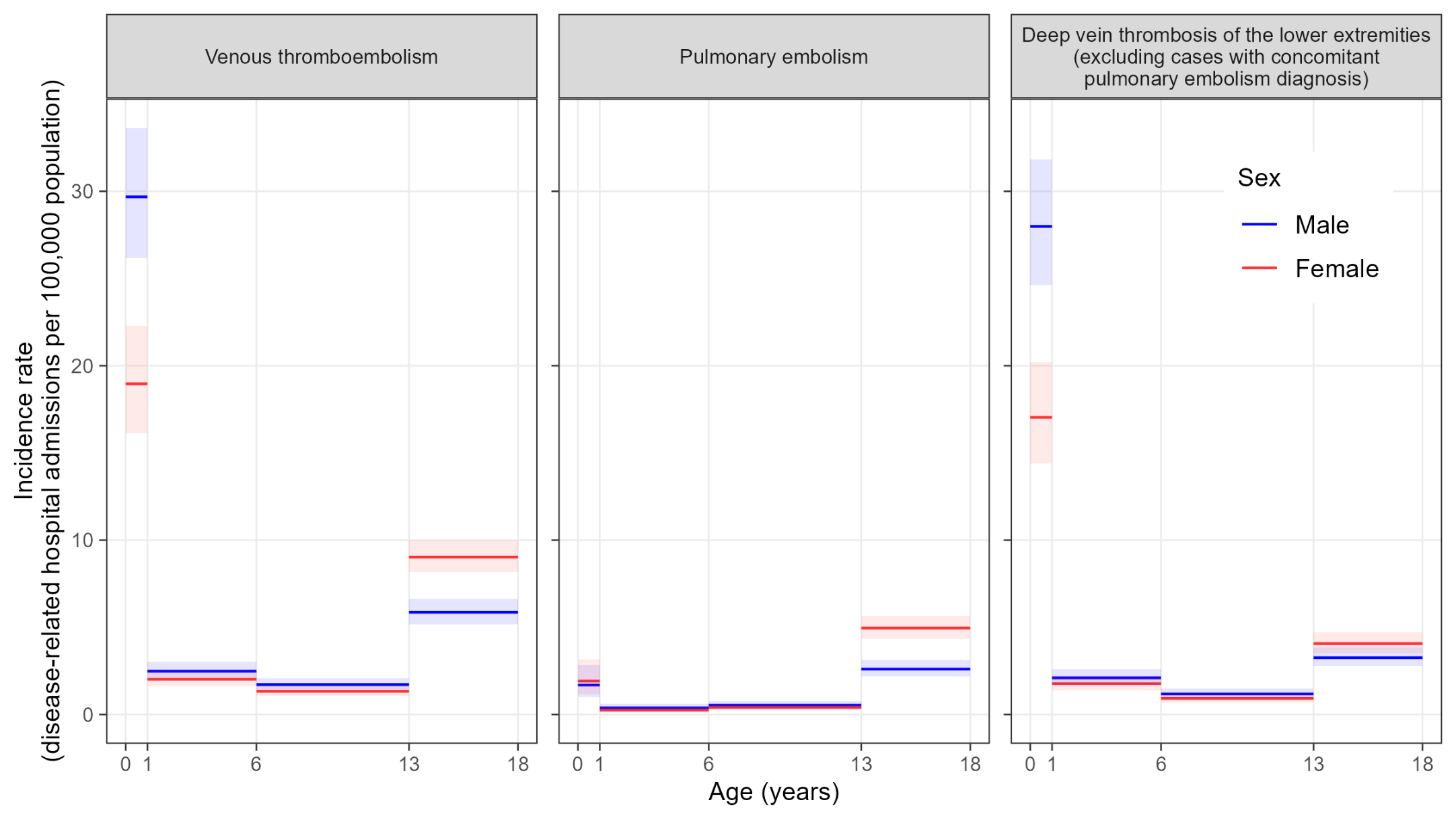


The shaded area depicts the 95% confidence interval.

# Figure S6. Proportion of venous thromboembolism (VTE)-related, pulmonary embolism (PE)-related, and deep vein thrombosis (DVT)-related hospitalizations out of all hospitalizations (VTE / PE / DVT-related hospital admissions per 10,000 hospital admissions) across clinically selected age groups in male and female patients.


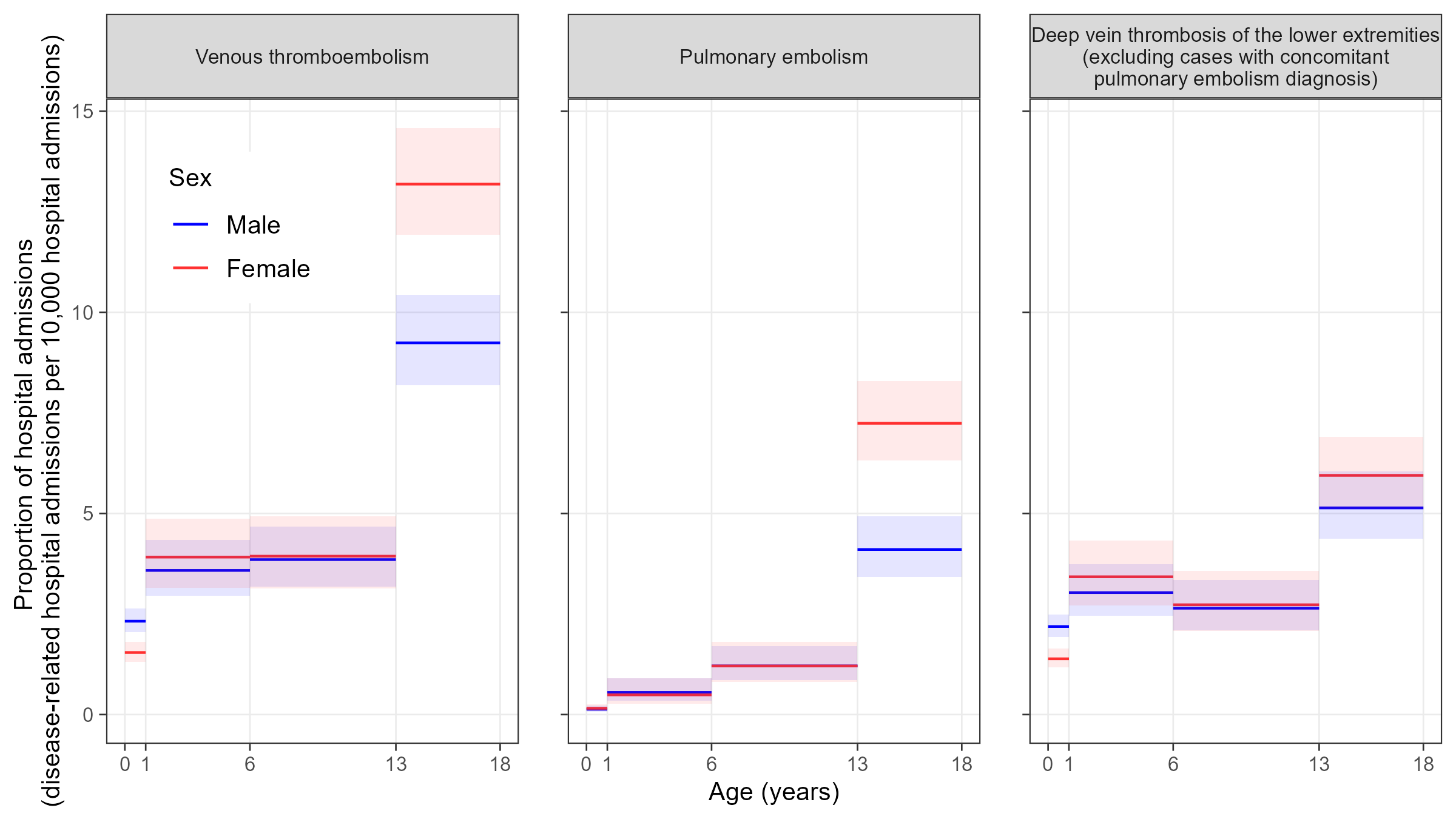
The shaded area depicts the 95% confidence interval.

# Figure S7. Pulmonary embolism (PE)-related and deep vein thrombosis (DVT)-related in-hospital case fatality rate (PE / DVT-related deaths per 100 PE / DVT-related hospital admissions) across age groups.


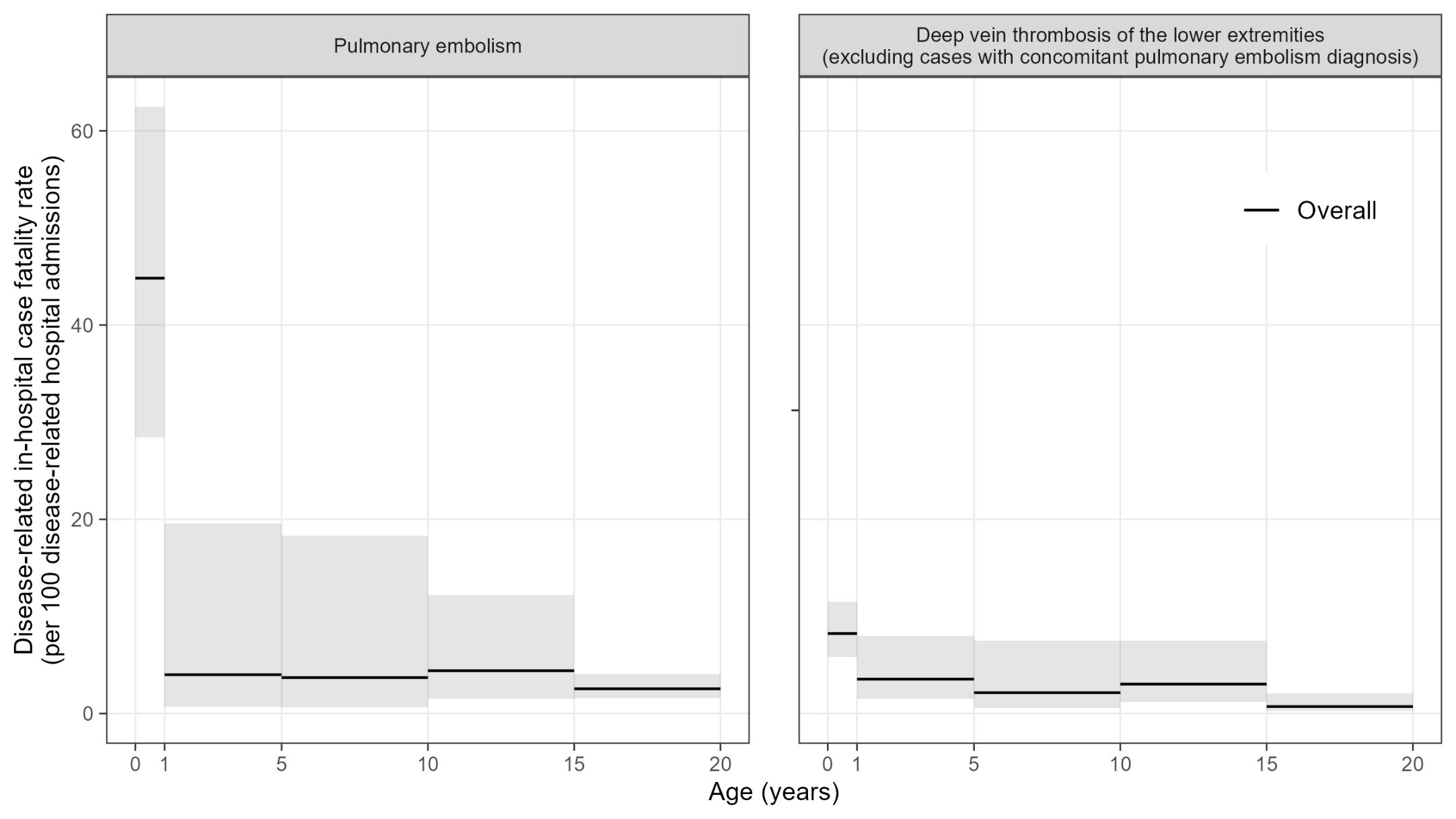
The shaded area depicts the 95% confidence interval.

# Figure S8. Venous thromboembolism (VTE)-related, pulmonary embolism (PE)-related and deep vein thrombosis (DVT)-related in-hospital case fatality rate (PE / DVT-related deaths per 100 PE / DVT-related hospital admissions) across clinically selected age groups.


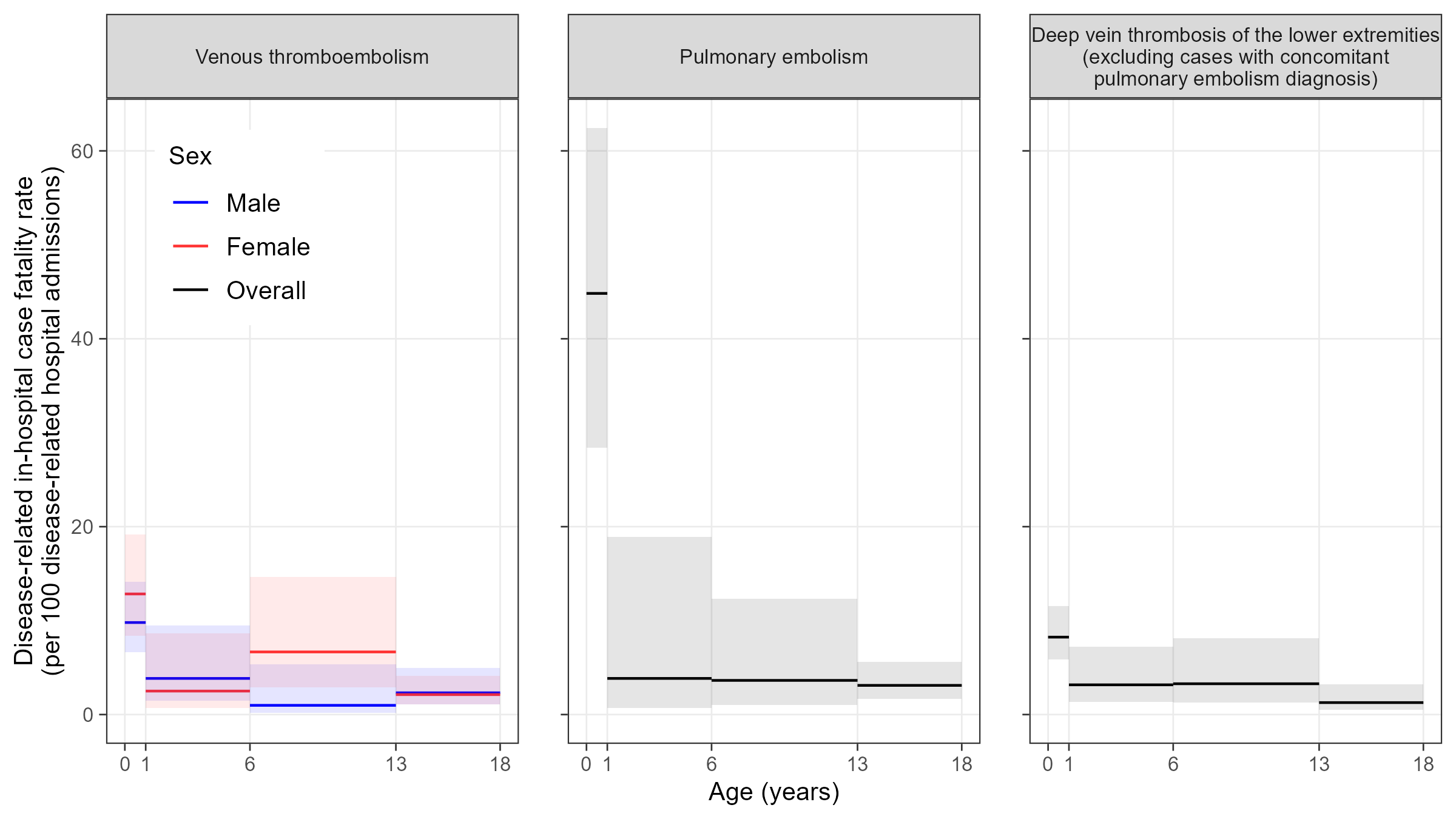
The shaded area depicts the 95% confidence interval.

# Figure S9. Median length of hospitalization (days) for patients with venous thromboembolism, pulmonary embolism, and deep vein thrombosis across age groups stratified by sex.


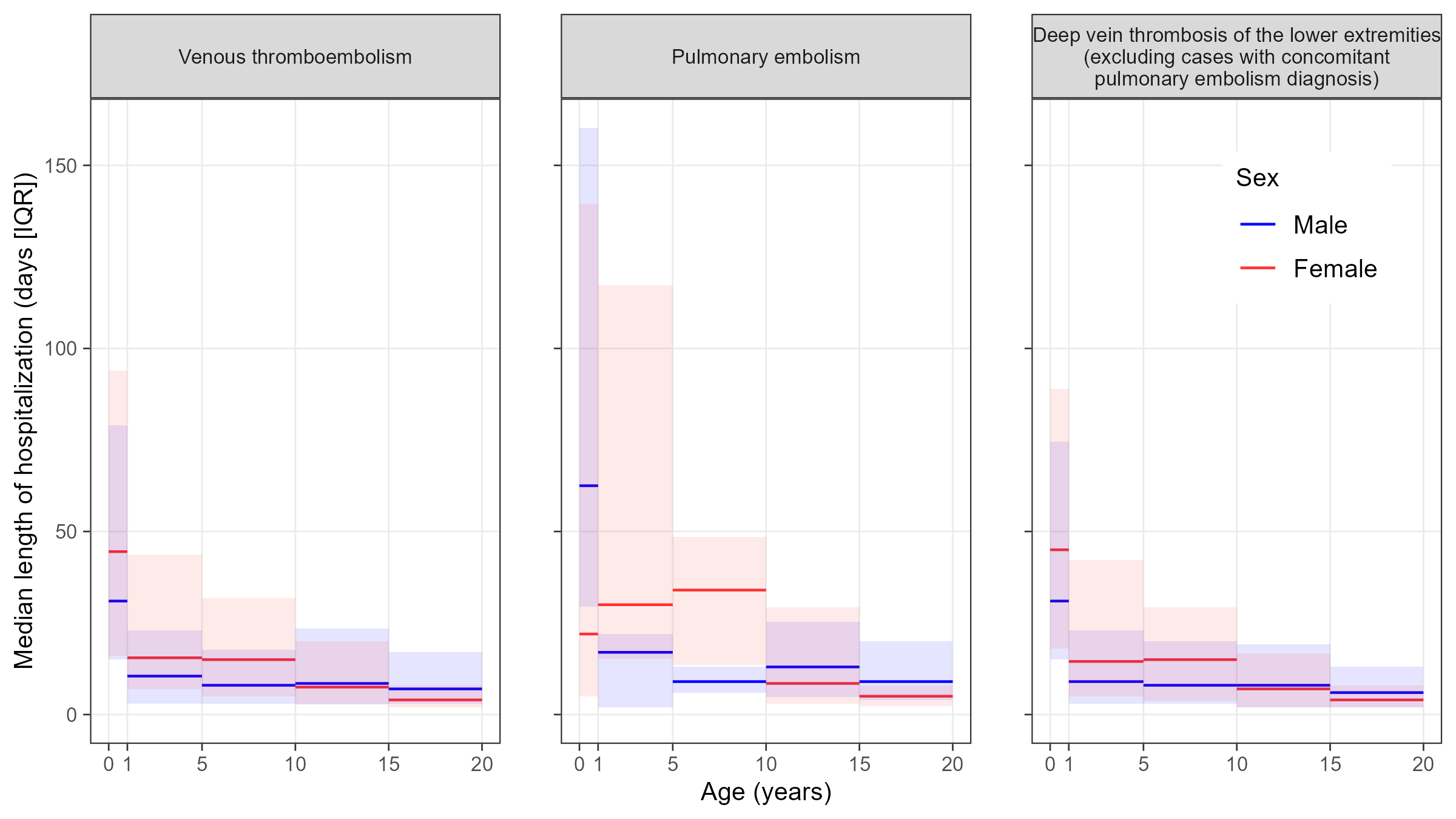


The shaded area depicts the interquartile range.

# Figure S10. Median length of hospitalization (days) for patients with venous thromboembolism, pulmonary embolism, and deep vein thrombosis across clinically selected age groups stratified by sex.


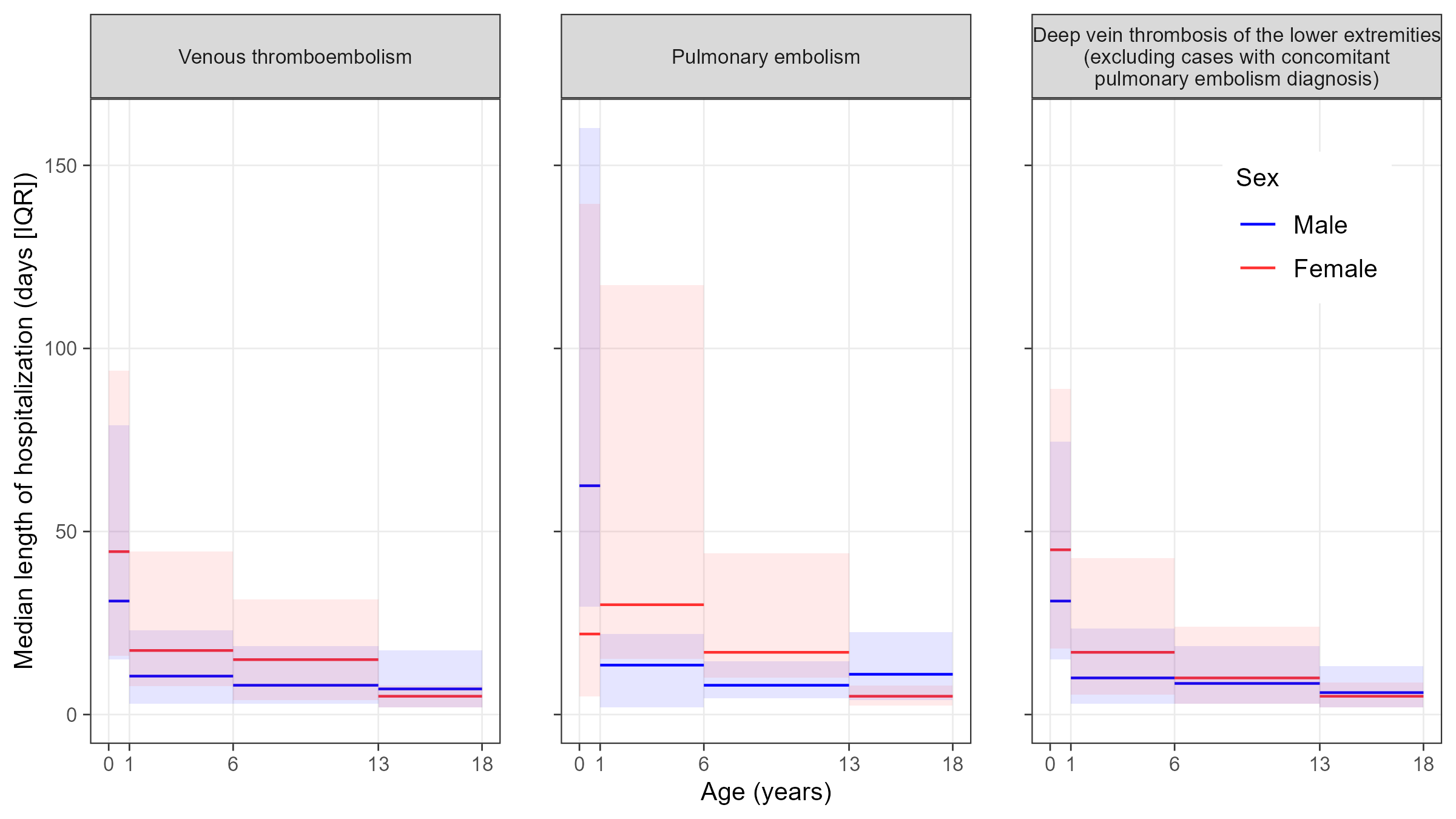
 The shaded area depicts the interquartile range.

# Figure S11. Proportion of intensive care unit (ICU) admissions for patients with venous thromboembolism, pulmonary embolism, and deep vein thrombosis across clinically selected age groups.


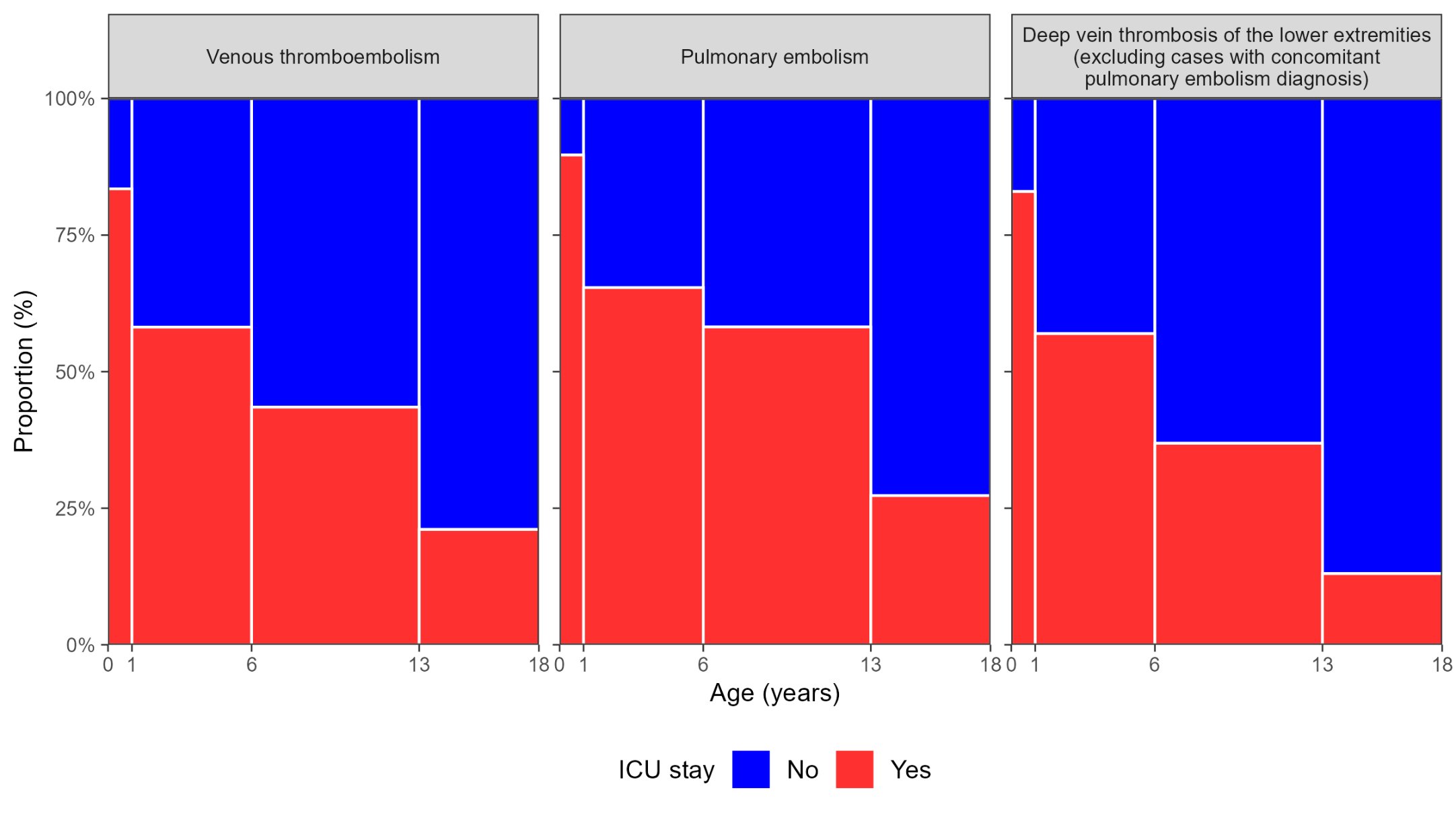


# Figure S12. Venous thromboembolism (VTE)-related, pulmonary embolism (PE)-related, and deep vein thrombosis (DVT)-related incidence rate (VTE / PE / DVT incident hospital admission per 100,000 children and adolescents per year) for VTE / PE / DVT as primary diagnosis at discharge across age groups stratified by sex.


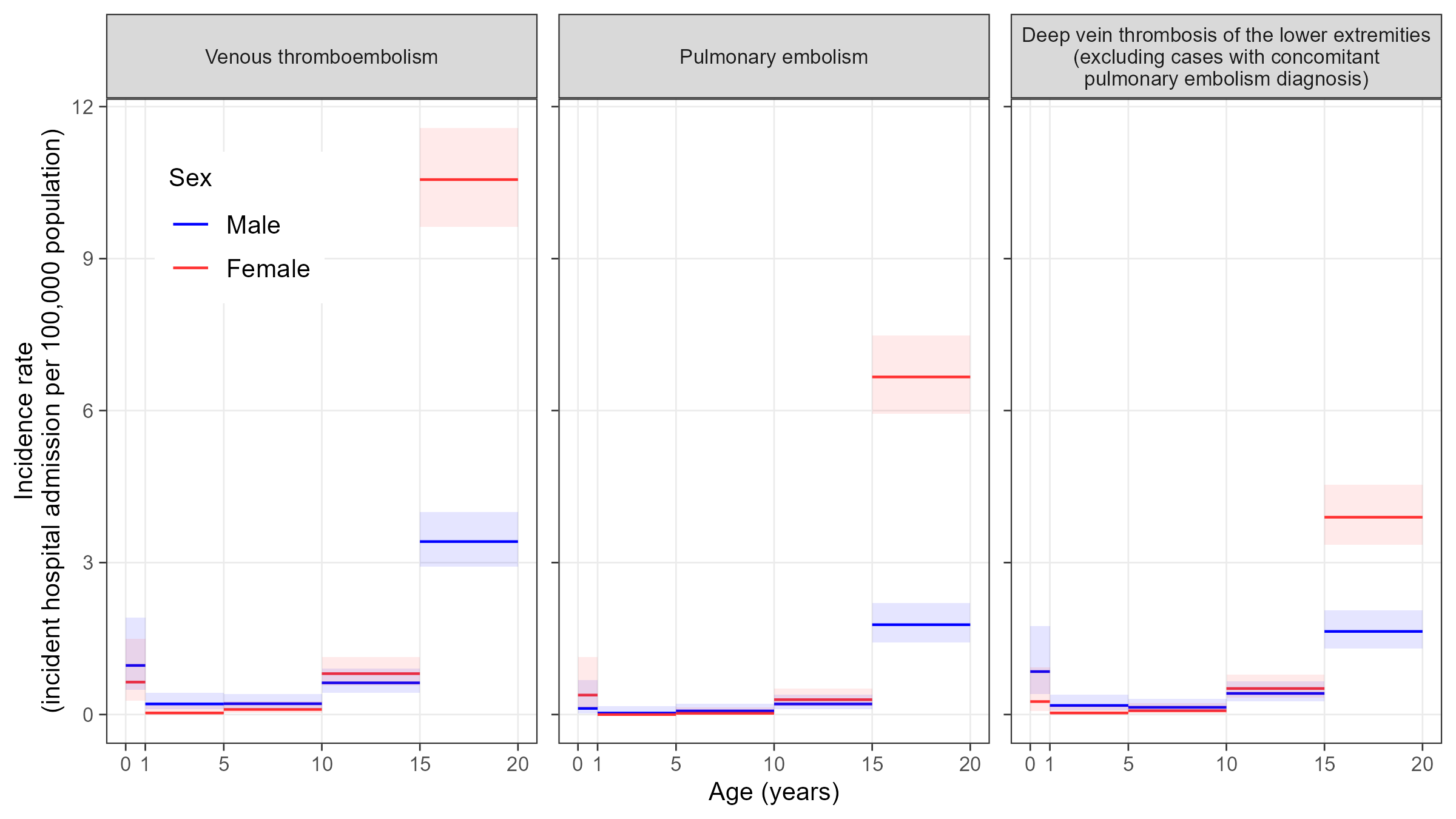


The shaded area depicts the 95% confidence interval.

# Figure S13. Proportion of venous thromboembolism (VTE)-related, pulmonary embolism (PE)-related, and deep vein thrombosis (DVT)-related hospitalizations (VTE / PE / DVT-related hospital admissions per 10,000 hospital admissions) for VTE / PE / DVT as primary diagnosis at discharge across age groups in male and female patients.


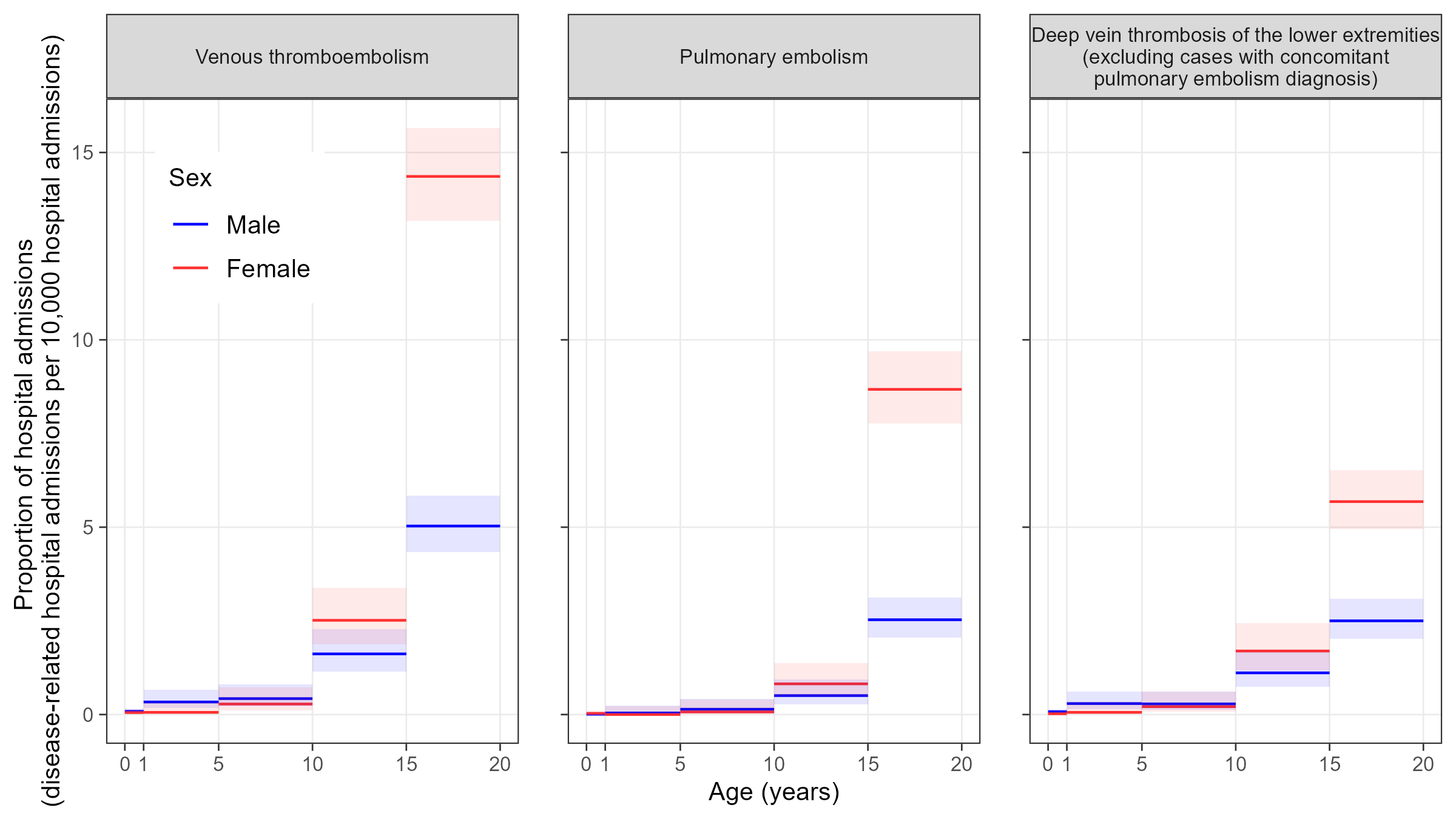
 The shaded area depicts the 95% confidence interval.
